# Extracellular vesicle-derived miRNA-182-5p educates macrophages towards an immunosuppressive phenotype in pancreatic cancer

**DOI:** 10.1038/s41392-025-02559-3

**Published:** 2026-01-16

**Authors:** Baldev Singh, Pankaj Gaur, Pritha Bose, Yanjun Zhang, Yaoxiang Li, Zihao Zhang, Jeyalakshmi Kandhavelu, William Klotzbier, Meth Jayatilake, Shivani Bansal, Mohd Farhan, Sunain Deol, Partha P. Banerjee, Keith Unger, Seema Gupta, Vivek Verma, Amrita K. Cheema

**Affiliations:** 1 Department of Oncology, Lombardi Comprehensive Cancer Center, Georgetown University Medical Center, Washington, DC USA; 2 Center for Advanced Immunotherapy Research, Lombardi Comprehensive Cancer Center, Georgetown University Medical Center, Washington, DC USA; 3 Department of Biochemistry, Molecular and Cellular Biology, Georgetown University, Georgetown University Medical Center, Washington, DC USA; 4 Department of Radiation Medicine, MedStar Georgetown University Hospital, Washington, DC USA; 5 The Hormel Institute, University of Minnesota, Austin, MN USA; 6 Center for Genome Engineering, University of Minnesota, Minneapolis, MN USA

**Keywords:** Cancer microenvironment, Gastrointestinal cancer

## Abstract

Reprogramming the immunosuppressive milieu in pancreatic cancer (PaCa) remains an important yet unmet therapeutic goal. Although tumor-associated macrophages (TAMs) are known to promote tumor growth and metastasis, little is known about the underlying mechanisms driving macrophage plasticity in PaCa. Herein, we show that extracellular vesicles (EVs) released by PaCa cells as well as circulating EVs in patient plasma, facilitate cellular crosstalk thereby promoting preferential skewing of recipient macrophages towards an M2-like TAM phenotype. PaCa-EV educated macrophages predominantly secrete anti-inflammatory cytokines, adapt an M2-like metabolic phenotype, have a higher expression of PD-L1, and suppress the proliferation of CD8^+^ T cells. An increased payload of miR-182-5p in PaCa-EV cargo causes a decrease in TLR4 expression in recipient macrophages and a concomitant upregulation of JAK/STAT3 pathway and elevated secretion of IL-10 and TGF-β, leading to increased PD-L1 expression. Most notably, targeted therapeutic delivery of antagomiR-182-5p in pancreatic tumor-bearing mice with varying immunogenic potential results in a significant decrease in tumor volume, increased survival, restoration of M1/M2 ratio, and an overall increase in CD8^+^ T cell activation in the TME. Taken together, we demonstrate a direct role of EVs in subverting the immune microenvironment and altering macrophage plasticity in a manner conducive to both tumor growth and proliferation. As such, a targeted delivery of microRNA inhibitors as drugs for altering macrophage plasticity may likely achieve better therapeutic response in pancreatic tumors.

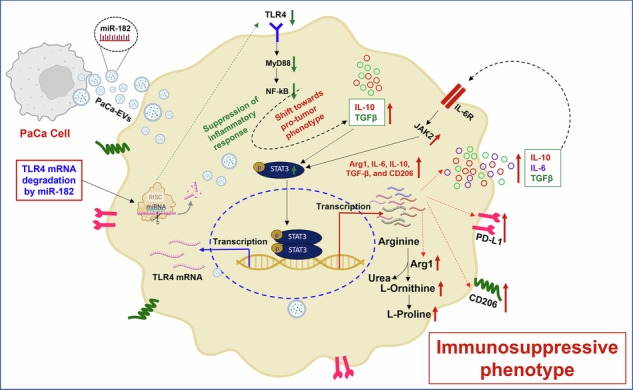

## Introduction

Pancreatic Cancer (PaCa) is one of the most difficult malignancies to treat, with an estimated 5-year survival rate of 13%.^1^ With a continuous rise in new cases and negligible improvement in overall survival rates, PaCa is predicted to be the second leading cause of cancer related death in the United States by the year 2030.^2–5^ The presence of dense stroma and diverse cell populations in the PaCa tumor microenvironment (TME), including more than 90% non-tumorigenic cells such as cancer-associated fibroblasts (CAFs), endothelial cells, and immune cells, contributes significantly towards resistance to traditional treatments like radiation and chemotherapy.^2,6–10^ Therefore, there is a strong need for the development of new therapeutic strategies against PaCa.

Macrophages are the most abundant immune cells within the PaCa stroma which play a pivotal role in shaping the immunosuppressive TME.^11–13^ Although the pancreatic TME is comprised of tumor-associated macrophages (TAMs) that originate from circulating monocyte-derived macrophages and tissue-resident macrophages, emerging evidence suggests that the latter play a significant role in contributing to tumor growth and metastasis.^14^ Macrophages are highly plastic and switch between classically activated (M1) and alternatively activated (M2) phenotypes^15^ that correlate with distinct secretory and metabolic profiles, as well as cell surface marker expression.^16–20^ For example, M1 polarized macrophages exhibit predominantly pro-inflammatory properties, anti-tumor effects, and high expression of nitric oxide synthase (NOS2). In contrast, M2 macrophages are associated with anti-inflammatory responses, promote tumor growth, and show increased expression of CD206 and arginase-1 (ARG1). TAMs largely resemble the M2 phenotype, although emerging literature suggests that they exhibit a mixed phenotype. TAMs promote the recruitment of other immunosuppressive cell populations, such as regulatory T cells and myeloid-derived suppressor cells, thereby promoting a highly immunosuppressive pancreatic TME.^21–23^ One potential mechanism of cross communication between PaCa cells and surrounding macrophages is the release of PaCa cell-derived extracellular vesicles (EVs), which act as conduits for modulating macrophage plasticity.^24^ EVs are small (40-1000 nm) membrane-bound particles that enclose a bioactive cargo comprised of proteins, nucleic acids, and metabolites. Proliferating tumor cells leverage EVs to actively communicate with surrounding cells to modulate TME towards growth, survival, and metastasis.^25–27^ The diversity of cargo packaged into EVs has the potential to induce an array of effects on recipient cells. Several studies have shown that tumor-derived EVs can be internalized by cells in the TME, including epithelial cells, fibroblasts, and endothelial cells, which resulted in altered phenotypes.^2,28,29^ EVs are also known to suppress or alter immune cell functions and are reported to facilitate the formation of a metastatic niche for pre-cancer cell infiltration.^29–31^ Although multiple studies have demonstrated the role of EVs in cellular crosstalk in tumor growth and metastasis,^32–36^ much needs to be discerned about how PaCa-derived EVs mediate pivotal crosstalk in the microenvironment to regulate the main hallmarks of tumorigenesis. This information is critical for devising novel therapeutic strategies as standalone or in combination with existing regimens for improving clinical outcomes.

We hypothesized that PaCa-derived EVs reprogram macrophages toward an M2-like TAM phenotype, upon uptake. Indeed, we found that the internalization of PaCa-EVs by bone marrow-derived macrophages (BMDMs) skews them towards an M2-like TAM phenotype, which is characterized by increased CD206 expression, elevated secretion of anti-inflammatory cytokines, and increased abundance of ornithine and proline, with a concomitant decrease in arginine levels, suggesting high Arginase1 activity.

PaCa-EV-treated macrophages suppressed CD8^+^ T cell proliferation in vitro and in vivo. Small RNA sequencing showed that the PaCa-EV cargo was highly enriched in miR-182-5p; a key driver of macrophage reprogramming. Delivery of exosomal miR-182-5p to the macrophages resulted in the downregulation of toll-like receptor 4 (TLR4) expression, upregulation of the Janus kinase 2 (JAK2), signal transducer, and activator of transcription 3 (STAT3) signaling pathway and elevated secretion of IL-10 and TGFβ. Ultimately, dysregulation of this signaling pathway in combination with alterations in secretory profiles of recipient macrophages led to increased expression of ARG1 and immunosuppressive ligand programmed cell death-ligand 1 (PD-L1) on the macrophage cell surface, thereby imparting an immunosuppressive TAM phenotype. Inhibition of miR-182-5p alleviated the TAM phenotype in vitro and a reduction in the number and size of PaCa tumor lesions in vivo. Overall, we present a paradigm for EVs that behave as “PaCa enablers” via modification of the TME. Reprogramming macrophage plasticity towards an anti-tumor phenotype by leveraging RNA-based therapeutic approaches, therefore, offers a high translational value in this difficult to treat cancer.

## Results

### PaCa-EVs reprogram macrophages to M2-like tumor-associated macrophage (TAM) phenotype

Given that cancer-derived EVs are known to interact with immune cells,^37–41^ we hypothesized that PaCa-derived EVs educate recipient macrophages towards an M2-like phenotype that helps establish an immunosuppressive pancreatic TME, supporting tumor growth and metastasis. To test this, EVs were isolated from cell culture media used to grow established human PaCa cell line (PANC-1), a PDX cell line (PPCL-68), a murine PaCa cell line (mT3-2D), and a non-tumorigenic pancreatic epithelial cell line (hTERT-HPNE) using size exclusion chromatography (SEC). The EV preparations were extensively characterized using nanoparticle tracking analysis (NTA), cryo-electron microscopy (cryo-EM), and immunoblot analysis for surface marker proteins as per Minimal Information for Studies of Extracellular Vesicles (MISEV) 2023 guidelines.^42^. We found that cancer cells secrete a higher number of EVs as compared to non-tumorigenic pancreatic epithelial cells (Supplementary Fig. 1a, b). The average particle size of EVs from all the cell lines was below 200 nm (Supplementary Fig. 1c, d). Immunoblot analysis confirmed the expression of EV markers^2,43,44^ including CD63, EpCAM, ANXA5, TSG101, FLOT1, ICAM, ALIX, and CD81 (Supplementary Fig. 1e, f). Pertinently, we observed higher expression of TSG101 and ALIX which are widely recognized as essential components of the endosomal sorting complex required for transport (ESCRT) pathway, and play a key role in exosome biogenesis.^45^ On the other hand, microvesicles generally lack these ESCRT-associated proteins since they bud directly from the plasma membrane.^45^ Higher expression of TSG101 and ALIX, thus indicates greater enrichment of exosomal fraction in our EV preparations. Further, cryo-EM was used to confirm the membrane integrity of EV preparations (Supplementary Fig. 1g). Taken together, the size distribution and surface marker expression data support the conclusion that our EV preparations are highly enriched in exosomes, with minimal contamination from microvesicles or apoptotic bodies.

**Fig. 1:**
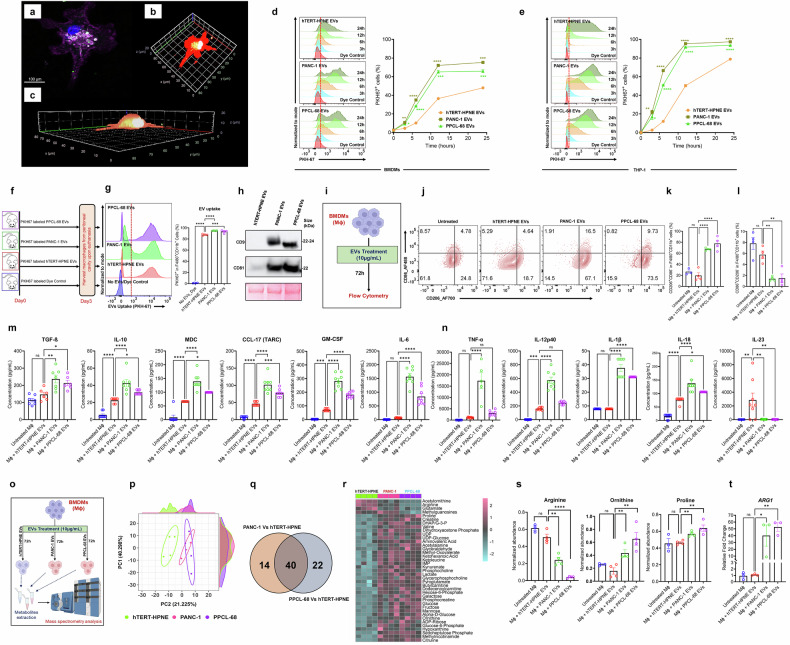
PaCa-EVs reprogram macrophages to M2-like tumor-associated macrophage (TAM) phenotype. **a–c** Representative confocal microscopy images of murine BMDMs treated with PKH67-labeled EVs (green), showing the uptake of EVs: BMDMs were stained with cell mask deep red (red), and nuclei were stained with Hoechst stain (blue). **a** A single stack of the 3D constructed image. **b** Birds-eye view. **c** Perpendicular view of macrophage. All images were captured after 24 h of EVs treatment at 63x magnification with pseudo color selection for each dye channel. PBS without any EVs mixed with PKH67 dye was used as a negative control. **d** Flow cytometry analysis shows time-dependent uptake of PKH67-labeled EVs by BMDMs at different time intervals (3, 6, 12, and 24 h post EV treatment). **e** Flow cytometry analysis shows time-dependent uptake of PKH67-labeled EVs by THP-1 macrophages at different time intervals (3, 6, 12, and 24 h post EV treatment). **f** Schematic of the strategy used to evaluate in vivo EV uptake by murine peritoneal cavity macrophages (PCMs). **g** Representative flowcytometry histogram and data showing uptake of PKH-67 labeled EVs by murine peritoneal cavity macrophages (PCMs) 72 h following intraperitoneal (I.P.) injection of PKH-67 labeled EVs. **h** Western blots of EVs, showing increased expression of CD9 and CD81 in hTERT-HPNE, PANC-1 and PPCL-68 EV cargo. **i** Schematic overview of EV treatment to BMDMs for M1 and M2 polarization analysis. **j** Representative flow cytometry plots. **k, l** Bar graphs showing CD206 and CD86 expression on EV (10 μg/mL) treated BMDMs after 72 h of treatment. **m** Concentration of anti-inflammatory (free active TGF-β1, IL-10, CCL22 (MDC), CCL17 (TARC), GM-CSF, and IL-6). **n** Pro-inflammatory cytokines (TNF-α, IL-12p40, IL-1β, IL-18, and IL-23) in the cellular supernatant of EVs treated BMDMs. **o** Schematic representation of targeted metabolomics experiment. **p** PCA analysis of metabolomic profiles of EV treated BMDMs. **q** Venn-diagram displaying the number of differentially altered metabolites in BMDMs after PANC-1 and PPCL-68 EV treatment as compared to the hTERT-HPNE EV treatment (FDR-adj. *p* ≤ 0.05 or *q* ≤ 0.05). **r** Heatmap of significantly altered metabolites of hTERT-HPNE, PANC-1, and PPCL-68 EVs treated BMDMs (*q* ≤ 0.05; *n* = 4). **s** Bar graphs highlighting the differences in intracellular abundance of selected metabolites of arginine and proline metabolic pathway in BMDMs after 72 h of EV treatment. **t** Arginase 1 (*ARG1*) gene expression in EV treated BMDMs. All data are presented as Mean ± SE, where *n* ≥ 4 per condition. ns = *p* > 0.05, * = *p* ≤ 0.05, ** = *p* ≤ 0.01, *** = *p* ≤ 0.001, and **** = *p* ≤ 0.0001 by one-way ANOVA, two-way ANOVA (EVs uptake experiment) and unpaired t-test (metabolomics data)

Next, we studied the uptake of EVs by performing an internalization experiment in which EVs were labeled with green fluorescent dye (PKH-67) and co-cultured with murine BMDMs as well as with human THP-1 cell line-derived macrophages under in vitro conditions. Confocal microscopy analysis performed 24 h after co-culture showed that all EV types (non-tumorigenic or PaCa-derived) were internalized by macrophages (Fig. 1a-c; Supplementary Fig. 1h, i; and Supplementary Fig. 2a, b). The human and murine EVs demonstrated preferential trafficking and accumulation around the perinuclear space near the endoplasmic reticulum (Supplementary Fig. 1h, i; 2a-c; and Supplementary Videos 1-4). Flow cytometry (FC) analysis showed that EV internalization by both murine and human macrophages was time-dependent, initiating as early as 3 h and peaking at approximately 12 h post-incubation with higher efficiency of uptake in PaCa-EVs compared to non-tumorigenic cell-EVs (Fig. 1d, e; Supplementary Fig. 2d-g gating strategy and EV uptake). Next, we evaluated EV uptake in vivo by intraperitoneal injection of PKH-67 labeled EVs into C57BL/6 mice (100 μg/mouse) by scoring for the presence of PKH-67^+^ peritoneal cavity resident macrophages (PCMs) by FC (Fig. 1f). PaCa-EVs demonstrated significantly higher (*p* ≤ 0.0001 in PANC1 and *p* ≤ 0.001 in PPCL-68) uptake rates compared to non-tumorigenic cell-derived EVs (Fig. 1g; gating strategies in Supplementary Fig. 3a), corroborating our in vitro findings. Since PaCa-EVs uptake rate was found to be higher than non-tumor EVs; hence, we evaluated CD9 and CD81 expression in our EVs preparations. These exosome markers have also been implicated in facilitating EV internalization by promoting membrane interactions.^46–48^ Interestingly, we observed elevated expression of CD9 and CD81 in PaCa-EVs compared to non-tumor EVs (Fig. 1h), which may, in part, facilitate their faster/increased uptake by macrophages.

To determine the effect of EV uptake on macrophage phenotypic spectrum, we used orthogonal approaches that included surface marker expression, mass spectrometry-based metabolomics as well as cytokine profiling. First, BMDMs were incubated with EVs for 72 h, followed by FC analysis to assess the expression of M1 (CD86) and M2 (CD206) markers (Fig. 1i, j; gating strategy in Supplementary Fig. 3b). We found that the uptake of PaCa-EVs by BMDMs resulted in a significant increase in CD206^+^ cell population, and a concomitant decrease in CD86^+^ cells compared to non-tumorigenic cell-derived EVs and PBS-treated BMDMs (Fig. 1k, l). These results were further confirmed using immunofluorescence analysis (Supplementary Fig. 3c). Furthermore, qRT-PCR analysis of murine PaCa-EV (mT3-2D EVs) treated BMDMs also showed higher relative gene expression of M2 marker gene, *ARG1* as compared to the untreated BMDMs. However, M1-associated markers *CD80* and *CD86* showed significant downregulation suggesting a skewing towards M2-like phenotype upon PaCa-EV uptake (Supplementary Fig. 3d). Next, we evaluated the effect of PaCa-EVs on human THP-1 macrophages by co-culturing and subsequent analysis of M2 marker expression using FC (Supplementary Fig. 3e). PaCa-EV treatment caused a significant increase in CD206 expression in THP-1 macrophages (Supplementary Fig. 3f; gating strategy in Supplementary Fig. 3g). Given that macrophage plasticity and/or polarization are characterized by changes in secretory and metabolic profiles, we evaluated cytokine profiles of cell culture supernatants from BMDMs and THP1 macrophages treated with EVs. Macrophages treated with PaCa-EVs showed a significant increase in the secretion of cytokines known to exhibit anti-inflammatory and/or tissue repair-promoting effects as compared to treatment with non-tumorigenic cell-derived EVs. These included transforming growth factor beta 1 (TGF-β1), interleukin 10 (IL-10), macrophage-derived chemokine (MDC), CCL-17, granulocyte-macrophage colony-stimulating factor (GM-CSF), and interleukin 6 (IL-6) (Fig. 1m; Supplementary Fig. 3h). Interestingly, PaCa-EVs also induced the secretion of selected pro-inflammatory cytokines, including tumor necrosis factor (TNF)-α, IL-12p40, IL-1β, and IL-18, except IL-23 (Fig. 1n; Supplementary Fig. 3h), corroborating a mixed macrophage phenotype that has been previously reported.^22,23,49^ These findings substantiate the emerging hypothesis that TAMs are highly plastic in nature and exhibit a spectrum of secretory phenotypes in the TME.^22,23^

Next, we sought to characterize the immunometabolic signature of PaCa and non-tumorigenic EV-treated BMDMs using multiple reaction monitoring mass spectrometry (MRM-MS) as described previously^19,20^ (Fig. 1o). Principal component analysis (PCA) showed a clear group separation of PaCa versus non-tumorigenic EV-treated macrophages, suggesting inherent differences in overall metabolic profiles (Fig. 1p). On the other hand, BMDMs treated with PaCa-EVs (PANC-1 and PPCL-68) clustered together, suggesting a common metabolic phenotype conferred by PaCa-EVs independent of the PaCa cell type. We identified differentially abundant metabolites (FDR-adj. *p* ≤ 0.05) in PANC-1 (*N* = 54) and PPCL-68 (N = 62) EV-treated BMDMs compared to those treated with hTERT-HPNE EVs (Fig. 1q and Supplementary Table 1). Among these, 40 metabolites were found to be commonly dysregulated in both PaCa-EV-treated groups (Fig. 1q, r). It is important to note that arginine was significantly depleted in PaCa-EV-treated BMDMs, with a concomitant increase in the intracellular abundance of ornithine and proline, which are hallmarks of M2-like metabolic phenotype^19,50–52^ (Fig. 1r, s). M2-like phenotype of TAMs is highly correlated with high ARG1 expression, which rapidly breaks down arginine to ornithine and urea.^53,54^ Not surprisingly, we found a significant elevation in *ARG1* expression in PaCa-EV-treated BMDMs (Fig. 1t; Supplementary Fig. 3d). Additionally, we performed MRM-MS based secretory metabolomics on the media supernatant used to culture BMDMs. We observed a group-based separation of PaCa and non-tumorigenic EV-treated macrophages (Supplementary Fig. 3i, Supplementary Table 2). Volcano plot visualization showed robust alterations in metabolite abundance in PaCa-EV groups as compared to the non-tumorigenic-EV treatment group (Supplementary Fig. 3j, k). Specifically, extracellular levels of arginine were reduced, whereas the relative abundance of lactate and citrulline was high (Supplementary Fig. 3l). Given that arginine is an important nutrient for T cells, deprivation of this metabolite by M2-like TAMs can lead to T cell suppression.^55,56^ Furthermore, lactate production by macrophages is known to induce PD-L1 expression, leading to the suppression of cytotoxic T cell function.^22^ These results strongly suggest that PaCa-EVs contain biomolecular cargo that confers metabolic reprogramming of macrophages, adversely impacting cytotoxic T cell function, and ultimately exacerbating an immunosuppressive TME.

### PaCa-EVs educated macrophages exert immunosuppressive functions in vitro and in vivo

**Fig. 2:**
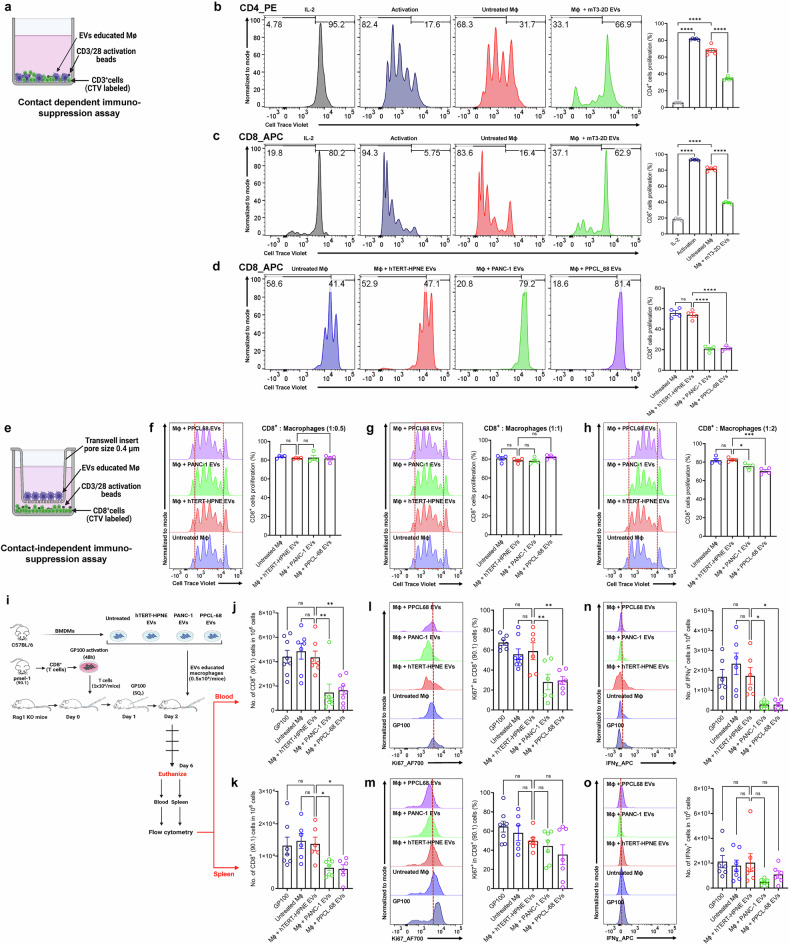
PaCa-EV educated macrophages exert immunosuppressive functions in vitro and in vivo. **a–f** PaCa cell derived EV educated macrophages suppress T cell proliferation. **a** Schematic illustration of contact dependent immuno-suppression assay. **b**, **c** Flow cytometry chart and data showing suppression of T cell (CD4^+^ and CD8^+^) proliferation after co-culturing (contact dependent manner) with mT3-2D EVs (murine PaCa-EVs) treated BMDMs at a ratio of 1:0.5. **d** Flow cytometry chart and data showing suppression of T cell (CD8^+^) proliferation after co-culturing (contact dependent manner) with PaCa-EV-treated macrophages at a ratio of 1:0.5. **e** Schematic illustration for contact independent immuno-suppression assay. **f–h** Flow cytometry plot and data showing T cell proliferation after co-culturing (contact independent manner) with PaCa-EV-treated macrophages. **i** Schematic representation of in vivo suppression assay. **j–o** *RAG1 KO* mice that received PaCa-EV educated macrophages show reduced T cell count (**j**, **k**), IFNɣ expression (**l**, **m**) and T cell proliferation (**n**, **o**), in blood and spleen cells respectively. All data is presented as Mean ± SE, where minimum *n* = 3 per treatment group. ns = *p* > 0.05, * = *p* ≤ 0.05, ** = *p* ≤ 0.01, *** = *p* ≤ 0.001, and **** = *p* ≤ 0.0001 by one-way ANOVA

Having established that PaCa-EVs drive macrophage plasticity towards an M2-like TAM phenotype, next, we sought to evaluate the effect of PaCa-EV educated macrophages on T cell proliferation. To explore this, EVs were enriched from media used to grow murine (m3T-2D) and human (PANC-1 and PPCL68) PaCa cell lines. Cell Trace™ Violet (CTV) labeled naïve (CD3^+^) T cells were co-cultured with BMDMs pretreated with or without EVs in the presence of anti-CD3/anti-CD28 beads (Fig. 2a). We found that PaCa-EV-treated BMDMs significantly suppressed CD4^+^ and CD8^+^ T cell proliferation compared to the untreated macrophages (Fig. 2b–d; gating strategies in Supplementary Fig. 4a, and respective IL-2 and activation controls for Fig. 2d are shown in Supplementary Fig. 4b). We next asked if PaCa-EVs directly impact T cell proliferation. We found that, naïve, CTV labeled T cells (CD3^+^ cells) cultured with different concentrations of murine PaCa-EVs (10, 20, and 30 µg/mL) did not show any changes in proliferation (Supplementary Fig. 4c, d) suggesting that the observed suppression effects are mediated *via* macrophages.

Given the central role of CD8^+^ T cells in mediating cytotoxic anti-tumor immunity, particularly relevant in the context of PaCa, subsequent suppression assays were conducted with CD8^+^ T cells. Furthermore, PaCa-EV-treated macrophages, when co-cultured with CD8^+^ T cells (separated by 0.4 µM transwell inserts) in different cell ratios (Fig. 2e), showed contact-independent suppression of CD8^+^ T cell proliferation, albeit at a lesser efficiency (Fig. 2f–h; respective IL-2 and activation controls in Supplementary Fig. 4e), compared to macrophages co-cultured directly with CD8^+^ T cells. These results suggest that contact-dependent immunosuppressive effects of PaCa-EV educated macrophages were more pronounced. However, increasing the macrophage:T-cell ratio resulted in contact-independent T cell suppression, suggesting that a minimum threshold of macrophage number may be needed for the secretion of immunosuppressive cytokines at a concentration that is sufficient for mediating CD8^+^ T cell suppression.

Next, to examine whether EV-treated macrophages exert their immunosuppressive activity in vivo, we used immunodeficient *Rag1* KO mice to minimize confounding variables introduced by a heterogeneous immune environment of the immunocompetent mice, enabling us to investigate the specific role of PaCa-EV educated macrophages in modulating cytotoxic CD8^+^ T cell responses. For this, *Rag1* KO mice were infused (I.V.) with 1 × 10^6^ gp100-activated pMel-1 CD8^+^ T cells at day 0.^57^ After 24 h (day 1), the mice were inoculated subcutaneously with gp100 peptide, followed by I.V. infusion of 0.5 × 10^6^ macrophages that were pretreated with or without EVs at day 2. Six days after infusion of CD8^+^ T cells, mice were euthanized for blood and spleen sample collection and analyzed for CD8^+^ T cells number, proliferation, and function using FC (schema shown in Fig. 2i; gating strategies are shown in Supplementary Fig. 4f). We found that mice that received PaCa-EV-treated macrophages had a significant decline in the numbers of viable CD8^+^ T cells in both the blood and spleen (Fig. 2j, k), coupled with significantly decreased numbers of proliferating Ki67^+^CD8^+^ T cells in the blood (Fig. 2l) compared to the control groups. A similar trend was observed in the spleen; however, the change was determined to be statistically non-significant (Fig. 2m). Further, CD8^+^ T cells from mice receiving PaCa-EV-treated macrophages also showed significantly decreased numbers of IFNγ^+^CD8^+^ T cells in the blood (Fig. 2n), with a similar representation in the spleen (Fig. 2o) as compared to mice that received non-tumorigenic-EV-treated and/or untreated macrophages, indicating that PaCa-EV-treated macrophages adversely impact the anti-tumor activity of CD8^+^ T cells.

### Immunosuppressive function of PaCa-EVs educated macrophages is PD-L1 dependent

**Fig. 3:**
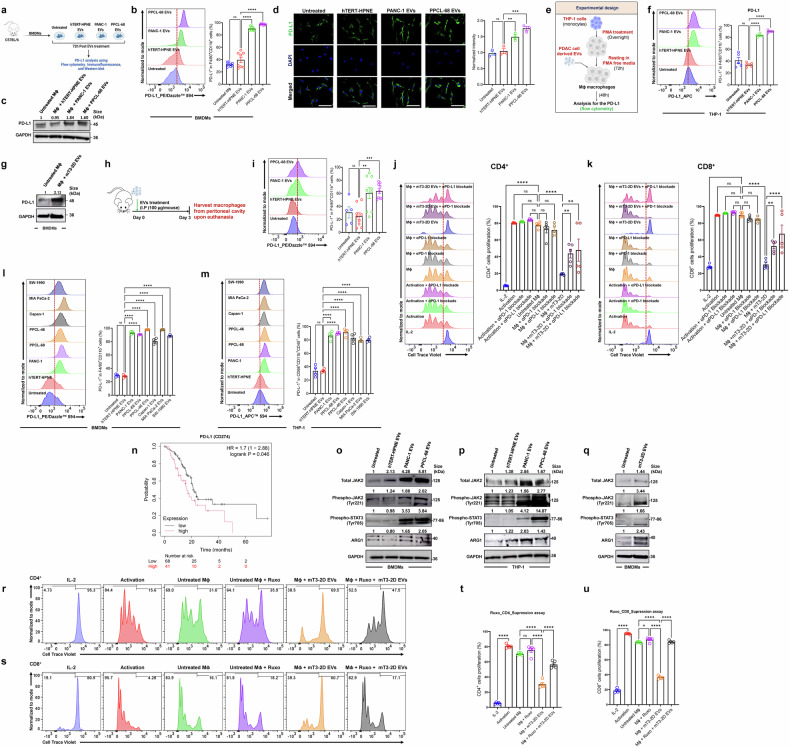
Immunosuppressive function of PaCa-EV educated macrophages is PD-L1 dependent. **a** Schematic representation of the experimental design. **b** Representative flow cytometry histogram and data showing higher expression of PD-L1 in PaCa-EV-treated BMDMs as compared to the untreated and non-tumorigenic cell line derived EV treated BMDMs. **c** Western blot analysis confirms higher expression of PD-L1 in PaCa-EV-treated BMDMs. **d** Immune fluorescence data also showing increased PD-L1 expression (higher green color fluorescence) in PaCa-EV treatment groups as compared to non-tumorigenic cell line derived EVs. **e** Schema of the experimental design used to analyze PD-L1 expression on THP-1 macrophages after EV treatment. **f** Flow cytometry analysis showing higher expression of PD-L1 on PaCa-EV-treated THP-1 macrophages. **g** Western blot analysis showing higher expression of PD-L1 in murine-PaCa-EV-treated BMDMs. **h** Schematic illustration of in vivo experiment, used to evaluate PD-L1 expression in murine peritoneal cavity macrophages (PCMs), 72 h post-EV treatment. **i** Representative flow cytometry histogram and bar graph showing a higher number of PD-L1 expressing PCMs in PaCa-EV injected mice compared to controls. **j**, **k** Flow cytometry data showing rescue of CD4^+^ and CD8^+^ suppression mediated by murine PaCa-EV educated macrophages in the presence of anti-PD-1 and anti-PD-L1 mAb. **l**, **m** Flow cytometry analysis showing higher expression of PD-L1 in BMDMs and THP-1 macrophages respectively, after different PaCa cell lines (PANC-1, PPCL-68, PPCL-46, CAPAN-1, MIA PaCa-2, and SW-1990) derived EV treatment as compared to controls. **n** Kaplan-Meier survival analysis of PD-L1 expression in patients diagnosed with pancreatic ductal adenocarcinoma (PDAC) with parameter of macrophage population. **o** Western blot analysis showing activation of proteins of the JAK/STAT3 signaling pathway, and increased arginase1 protein expression in PANC-1 and PPCL-68 EV treated BMDMs. **p** Western blots of THP-1 macrophages, showing increased expression of phsophoJAK2 and phosphoSTAT3 and ARG1 following PANC-1 and PPCL-68 EV treatment. **q** Western blots of BMDMs, showing increased expression of phsophoJAK2 and phosphoSTAT3 and ARG1 following mT3-2D EV treatment **r–u** Small molecule inhibition of JAK/STAT3 signaling in prior to PaCa (mT3-2D) EV treatment rescues T cell (CD4^+^ (**r**, **t**) and CD8^+^ (**s**, **u**)) suppression in BMDMs (*n* = 5). All data are presented as Mean ± SE, where minimum *n* = 3 per treatment group. ns = *p* > 0.05, * = *p* ≤ 0.05, ** = *p* ≤ 0.01, *** = *p* ≤ 0.001, and **** = *p* ≤ 0.0001 by one-way ANOVA

Given that PD-L1 is an important checkpoint molecule expressed on the surface of TAMs, which plays a critical role in T cell suppression,^58^ we asked if PaCa-EV treatment of BMDMs and THP-1 macrophages would result in increased PD-L1 expression (Fig. 3a). FC analysis of PaCa-EV-treated BMDMs showed significantly higher PD-L1 expression compared to both hTERT-HPNE EV-treated and untreated macrophages (Fig. 3b). These results were further confirmed by western blot and immunofluorescence (IF) analysis (Fig. 3c, d). Similarly, THP-1 macrophages treated with human PaCa-EVs (Fig. 3e, f) and BMDMs treated with murine PaCa-EVs also showed elevated PD-L1 expression (Fig. 3g). To validate these findings in vivo, EVs were intraperitoneally injected into C57BL/6 mice (100 μg/mouse) and macrophages were harvested from the peritoneal cavity after 3 days (Fig. 3h). Mice peritoneal cavity macrophages (PCMs) that received PaCa-EVs showed a significant increase in PD-L1^+^ population as compared to untreated mice or those that received hTERT-HPNE-EVs (Fig. 3i). Moreover, blocking PD-1 or PD-L1 interaction between T cells and BMDMs using anti-PD-1 and anti-PD-L1 antibodies resulted in a significant reduction in CD4^+^ and CD8^+^ T cell suppression caused by murine PaCa-EV-treated macrophages under in vitro conditions (Fig. 3j, k). Similar results were also observed when human PaCa-EV-treated BMDMs were co-cultured with CD8^+^ T cells in the presence of anti-PD-1 and anti-PD-L1 antibodies (Supplementary Fig. 5a; respective IL-2 and activation controls in Supplementary Fig. 5b). Concomitantly, PaCa-EV-treated BMDMs showed elevated secretion of IL-10 and TGF-β (Fig. 1m), both of which are known to positively regulate PD-L1 expression.^59,60^ To further confirm these observations, we blocked IL-10 and TGF-β using respective antibodies and found a significant rescue of CD8^+^ T cell suppression compared to CD8^+^ T cells cultured with untreated macrophages (Supplementary Fig. 5c). These results suggest that PaCa-EVs partially mediate their impact by promoting the secretion of immunosuppressive cytokines, such as IL-10 and TGF-β by the recipient macrophages, which results in increased PD-L1 expression.

Next, we asked whether EVs from PaCa cell lines with divergent genotypes would skew macrophage plasticity and result in increased PD-L1 expression on the cell surface. To test this, EVs were isolated from an array of PaCa cell lines, including PPCL-46, Capan-1, MIA PaCa-2, and SW-1990, and characterized using NTA and immunoblot analysis for EV marker proteins (Supplementary Fig. 5d, e). We found that BMDMs treated with EVs isolated from all the PaCa cell lines showed increased CD206-expressing macrophage population as compared to BMDMs treated with the non-tumorigenic cell line-derived EVs (Supplementary Fig. 5f). Subsequently, BMDMs and THP-1 macrophages were co-cultured with PaCa-EVs from each cell line for 72 h. We found that treatment with all PaCa cell line-derived EVs resulted in elevated PD-L1 expression in both BMDMs and THP-1 macrophages (Fig. 3l, m).

We asked if a higher percentage of PD-L1-expressing macrophages in pancreatic TME would impact overall survival in patients with a diagnosis of pancreatic ductal adenocarcinoma (PDAC). We leveraged Kaplan-Meier Plotter^61^ based analysis to determine gene expression associated with survival, wherein query parameters were restricted to macrophage-enriched based cellular content in PDAC for PD-L1 (*CD274*) gene expression. This analysis showed that a higher PD-L1 expressing pancreatic TME significantly correlated with reduced survival in PDAC patients, which lends strong translational credence to our findings (Fig. 3n). Together, these results demonstrate that PaCa-EV educated macrophages express higher level of ligands like PD-L1 on the cell surface that may exacerbate an immunosuppressive microenvironment, potentially hindering the effectiveness of immunotherapeutic approaches used for PaCa treatment.

### PaCa-EV-induced JAK2/STAT3 signaling exacerbates immunosuppressive phenotype of recipient macrophages

Next, we sought to understand the molecular mechanisms underlying PaCa-EV driven alterations in macrophage plasticity toward an immunosuppressive phenotype. For this, PaCa-EV-treated BMDMs (48 h post-treatment) were subjected to RNAseq analysis (Supplementary Fig. 5g–j). PCA plot yielded unambiguous group separation, suggesting that gene expression profiles of PaCa-EV-treated BMDMs were distinct from hTERT-HPNE-EV-treated BMDMs (Supplementary Fig. 5g). Gene set enrichment analysis (GSEA) identified 3,011 and 2,862 differentially expressed genes (FDR-adj. *p* ≤ 0.05) in PANC-1 and PPCL-68 EV-treated BMDMs respectively as compared to the hTERT-HPNE-EV-treated BMDMs. We focused on further analysis of 2,413 genes that were commonly and concordantly dysregulated in both PANC-1 and PPCL-68 EV treatment groups (Supplementary Fig. 5h). Interestingly, key anti-inflammatory pathway related gene expression (*MRC1* (*CD206*), and *IL-10*) was found to be upregulated in PaCa-EV-treated BMDMs, while pro-inflammatory pathways related genes (*CD86*, *MYD88*, and *NFKB*) were downregulated (Supplementary Fig. 5i). Gene ontology (GO) analysis of the commonly dysregulated genes using Molecular Signatures Database (MSigDB)^62^ showed alteration in interferon-γ response, TNF-α, and IL-6/JAK/STAT3 signaling pathways (Supplementary Fig. 5j). Since emerging evidence supports the role of JAK2/STAT3 signaling in the reprogramming of macrophages to an M2-like phenotype^63–68^ and the activation of STAT3 is linked to higher PD-L1 expression in macrophages,^69–73^ we questioned whether PaCa-EV driven M2-like TAM polarization and elevated PD-L1 expression are mediated by STAT3 activation. Western blot analysis of human or murine PaCa-EV-treated BMDMs and THP-1 macrophages revealed an upregulation of JAK2, phospho-JAK2 (pJAK2), phospho-STAT3 (pSTAT3), and ARG1, suggesting that JAK2/STAT3 is a likely mediator of the resultant M2-like TAM phenotype (Fig. 3o–q). Furthermore, inhibition of the JAK2/STAT3 signaling in BMDMs using ruxolitinib (RUXO)^74^ (Supplementary Fig. 5k), 2 h prior to treatment with PPCL-68-EVs significantly reduced ARG1, PD-L1, and pSTAT3 expression (downstream of JAK2) in BMDMs (Supplementary Fig. 5l), underscoring the role of JAK2/STAT3 signaling in reprogramming of PaCa-EV-treated macrophages to M2-like TAM phenotype. Subsequently, we explored whether inhibiting JAK2/STAT3 in macrophages would impact T cell suppression capability. To this end, we performed a T cell suppression assay in vitro and found that the BMDMs that received RUXO treatment, prior to treatment with EVs derived from mT3-2D and PPCL-68 cells, were less immunosuppressive as demonstrated by increased T cell proliferation compared to BMDMs that were not treated with RUXO (Fig. 3r–u and Supplementary Fig. 5m). However, RUXO treatment alone had no effect on the suppressive potential of the BMDMs (Fig. 3t, u and Supplementary Fig. 5m). Taken together, these findings highlight the important role of JAK2/STAT3 signaling cascade in PaCa-EV-mediated modulation of macrophages to TAM phenotype.

### miR-182-5p enriched in PaCa-EVs is a key driver for inducing M2-like TAM phenotype

**Fig. 4:**
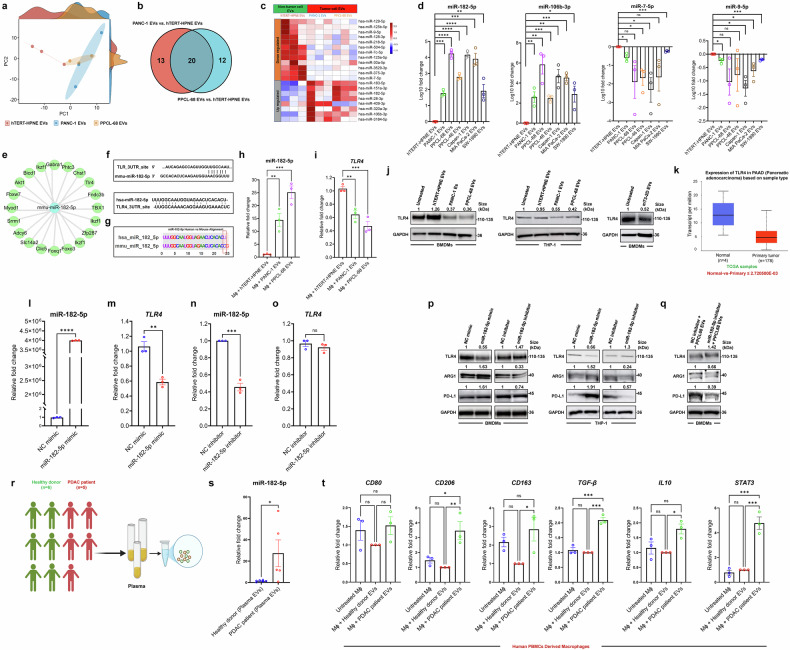
miR-182-5p enriched in PaCa-EVs drives M2-like TAM phenotype in BMDMs and THP-1 macrophages. **a** PCA of microRNAs (miRNAs) expression profiling of EV-cargo derived from hTERT-HPNE, PANC-1 and PPCL-68 cell lines. **b** Venn-diagram showing the number of differentially expressed microRNAs in PANC-1 and PPCL-68 EVs as compared to the hTERT-HPNE EVs (q ≤ 0.05). **c** Heatmap of significantly up and downregulated miRNAs in EV cargo of hTERT-HPNE, PANC-1 and PPCL-68 EVs (*n* = 3). **d** qRT-PCR validation of two significantly upregulated (miR-182-5p and miR-106b-5p) and two significantly down regulated (miR-7-5p and miR-9-5p) miRNAs in different PaCa cell derived EVs (PANC-1, PPCL-68, PPCL-46, CAPAN-1, MIA PaCa-2, and SW-1990) as compared to the non-tumorigenic pancreatic epithelial cell line (hTERT-HPNE) (*n* = 3). **e** Putative target of miR-182-5p **f** miR-182-5p aligned with TLR4 to show the putative binding site corresponding to the 3’ region of the TLR4 mRNA. **g** Homology modelling of murine and human miR-182-5p showing only one base pair difference. **h**, **i** Expression profile of the miR182-5p and TLR4 in BMDMs after EV treatment. **j** Western blot analysis of TLR4 in BMDMs and THP-1 macrophages after EV treatment. **k** TCGA analysis shows decreased TLR4 expression in PDAC tumors in comparison to the normal tissue controls. **l** Expression profile of miR-182-5p and (**m**) mRNA expression of TLR4 in BMDMs treated with miR-182-5p mimic as compared to the negative control (NC) mimic (*n* = 3). **n** Expression profile of miR-182-5p and (**o**) TLR4 in BMDMs treated with miR-182-5p inhibitor as compared to the negative control (NC) inhibitor (*n* = 3). **p** Western blots showing ARG1, PD-L-1, and TLR4 expression in miR-182-5p mimic and inhibitor transfected BMDMs and THP-1 macrophages. **q** Western blots showing restoration of TLR4 expression with a concomitant decrease in ARG1, and PD-L-1 expression in miR-182-5p inhibitor transfected BMDMs (24 h), followed by 48 h of PPCL-68 EVs treatment. **r** Schematic representation of the experiment performed with PaCa patient cohort. **s** qRT-PCR analysis of miR-182-5p in healthy donors and PaCa patient plasma derived EVs. **t** qRT-PCR analysis of *CD80*, *CD206, CD163*, *TGF-β IL-10* and *STAT3* mRNA expression in human PBMCs derived macrophages after PaCa patient plasma derived EVs treatment as compared to healthy donors and untreated controls. Data are presented as Mean ± SE, where *n* = 3 per condition, unless specified. ns = *p* > 0.05, * = *p* ≤ 0.05, ** = *p* ≤ 0.01, *** = *p* ≤ 0.001 and **** = *p* ≤ 0.0001by unpaired t test

Recently, cancer-derived exosomal small RNAs have gained increasing traction in the modulation of cellular function and behavior of recipient cells.^12,75,76^ We performed small/micro-RNA sequencing of EVs derived from PaCa cell lines (PANC-1 and PPCL-68) and non-tumorigenic pancreatic epithelial cell line, hTERT-HPNE. PCA analysis revealed clear group separation, indicating distinct microRNA (miRNA) expression profiles of PaCa and non-tumorigenic EV-cargo (Fig. 4a). We focused on 20 commonly differentially expressed miRNAs across the two PaCa cell lines (Fig. 4b and Supplementary Table 3), of which 12 miRNAs were downregulated and 7 were upregulated in PaCa-EVs derived from both the cell lines (with 1 differentially regulated in PaCa-EVs derived from the two PaCa cell lines) compared to non-tumorigenic EVs (Fig. 4c). We selected a subset of two upregulated (miR-182-5p and miR-106b-3p) and two downregulated (miR-7-5p and miR-9-5p) miRNAs for qRT-PCR based validation in an array of pancreatic cancer cell line derived EVs, including from PPCL-46, Capan-1, MIA PaCa-2, and SW-1990. The expression and abundance of all four miRNA targets are corroborated with small RNA sequencing data from PANC-1 and PPCL-68 EVs (Fig. 4d). Given the previously reported role of miR-182-5p in driving immunosuppression,^77^ we decided to investigate this further since it was significantly enriched in PaCa-EV cargo. Target prediction analysis using miRTargetLink 2.0^78^ revealed toll-like receptor-4 (TLR4) as one of the direct targets for miR-182-5p (Fig. 4e). Subsequent target site prediction analysis using TargetScan^79^ and RNA22^80^ as the target site for miR-182-5p in human and mice respectively also confirmed TLR4 as a target for miR-182-5p (Fig. 4f). The human and mouse miR-182-5p exhibit a high sequence homology of 96% (Fig. 4g). Given that TLR4 is known to promote inflammatory responses in macrophages,^81^ we posited that miR-182-5p enriched in PaCa-EVs drives macrophage reprogramming, in part, by downregulation of TLR4. Indeed, we found that PaCa-EV-treated BMDMs showed elevated levels of miR-182-5p and reduced *TLR4* expression in comparison to non-tumorigenic-EV treated samples (Fig. 4h, i). TLR4 downregulation in PaCa-EV-treated BMDMs and THP-1 macrophages was further confirmed at the protein level by western blot analysis (Fig. 4j). Pertinently, TCGA analysis using UALCAN^82,83^ showed decreased expression of *TLR4* in PDAC tumor tissues compared to the healthy pancreatic tissue samples (Fig. 4k). Taken together, these findings strongly implicate the miR-182-5p/TLR4 axis in altering macrophage plasticity towards M2-like TAMs in PaCa.

To further evaluate the role of exosomal miR-182-5p in skewing macrophage plasticity towards an M2-like TAM phenotype, BMDMs and THP-1 macrophages were transfected with a corresponding murine and human miR-182-5p mimic and inhibitor, along with respective negative controls (NC). qRT-PCR analysis of transfected cells (24 h after transfection) showed higher expression of miR-182-5p and reduced *TLR4* mRNA expression in miR-182-5p mimic-transfected samples when compared to the NC (Fig. 4l, m). Inversely, miR-182-5p inhibitor transfection led to reduced miR-182-5p expression as compared to the NC inhibitor transfection and rescue of *TLR4* expression (Fig. 4n, o). Furthermore, western blot analysis of macrophages transfected with miR-182-5p mimic revealed an upregulation of ARG1 and PD-L1 and downregulation of TLR4 expression (Fig. 4p). Reciprocally, miR-182-5p inhibitor treatment resulted in the downregulation of ARG1 and PD-L1 as compared to their respective NC controls (Fig. 4p). FC analysis of miR-182-5p mimic transfected BMDMs also showed elevated levels of CD206 and PD-L1 expression (Supplementary Fig. 6a), while BMDMs transfected with the inhibitor of miR-182-5p showed decreased expression of these markers (Supplementary Fig. 6b). Inhibition of miR-182-5p in BMDMs increased TLR4 expression and reduced both ARG1 and PD-L1 expression, which was not amenable to alleviation by subsequent PPCL-68-EV treatment (Fig. 4q). Interestingly, inhibition of miR-182-5p in BMDMs also rescued IL-4 + IL-13 mediated M2 polarization, as shown by expression of TLR4 and ARG1 (Supplementary Fig. 6c). These data corroborate our hypothesis that exosomal miR-182-5p from PaCa-EVs potentially mediates preferential skewing of macrophages towards an M2-like TAM phenotype via the TLR4 pathway. To our knowledge, this has not been reported in the context of PaCa.

We then built upon these novel findings and evaluated CD45^+^CD11b^+^ and F4/80^+^ macrophage infiltration, expression of M1/M2 markers, PD-L1 expression, and miR-182-5p in vivo in the pancreas of tumor-bearing and healthy mice. For this, we implanted Pan02_luciferase orthotopic tumors in C57BL/6 mice (*n* = 4). Tumor growth was monitored by IVIS (In Vivo Imaging System) and ultrasound imaging (Supplementary Fig. 6d (i-iv)). Pancreas were harvested from healthy and tumor-bearing mice on day 42 (Supplementary Fig. 6d (v and vi)), followed by FC analysis. We found an increased infiltration of CD45^+^CD11b^+^ and F4/80^+^ macrophages into the pancreas of tumor-bearing mice as compared to healthy controls (Supplementary Fig. 6e; gating strategies in Supplementary Fig. 6f). Tumor-infiltrating macrophages also showed higher CD206 (M2 marker) and PD-L1 expression, with a concomitant reduction in CD86 (M1 marker) expression (Supplementary Fig. 6e). miR-182-5p expression was also found to be upregulated in the pancreas of tumor-bearing mice (Supplementary Fig. 6g). Further, to validate these findings in humans, we isolated EVs from blood plasma samples of PaCa patients as well as from healthy donors and characterized using NTA and immune marker analysis (Fig. 4r and Supplementary Fig. 6h, i) (cohort details are provided in Supplementary Fig. 6j) and evaluated miR-182 expression. We found significantly higher abundance of miR-182-5p in PaCa patient plasma-derived EVs compared to the healthy donor plasma-derived EVs (Fig. 4s). Next, to test the functional impact of EVs on macrophages, we treated human PBMCs-derived macrophages with blood plasma-derived EVs. We observed a significant increase in the levels of *CD206*, *CD163*, *TGF-β, IL-10, and STAT3* mRNA in macrophages treated with PaCa patient-derived EVs as compared to the healthy donor EVs, as well as untreated macrophages (Fig. 4t), whereas CD80 (M1 marker) did not show any significant difference (Fig. 4t). Together, these data highlight the role of PaCa-derived exosomal miR-182-5p in macrophage programming towards an immunosuppressive phenotype in PaCa.

### Inhibition of miR182-5p in PaCa cells impairs the ability of PaCa-EVs to polarize macrophages to M2-like TAM phenotype

**Fig. 5:**
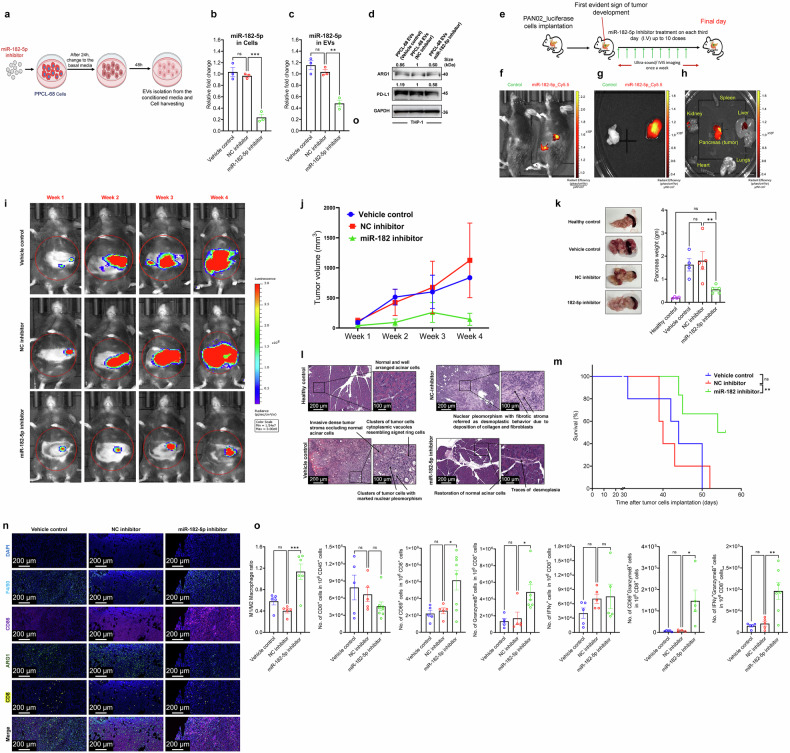
Inhibition of miR182-5p in PaCa cells impairs the ability to polarization of macrophages to M2-like TAM phenotype **a** Schematic of the experiment used to generate PaCa-EVs with reduced miR-182-5p cargo. **b** qRT-PCR data showing reduced miR-182-5p expression in PPCL-68 cells after miR-182-5p inhibitor treatment. **c** qRT-PCR data showing reduced miR-182-5p EV-cargo payload derived from miR-182-5p inhibitor-treated PPCL-68 cells. **d** Western blot analysis showing reduced ARG1 and PD-L1 expression in THP-1 macrophages treated with PPCL-68 EV having reduced miR-182-5p EV-cargo payload. **e** Schematic representation of in vivo experiment conducted to evaluate the effect of miR-182-5p antagomir on tumor growth. **f–h** Representative IVIS images, in vivo (**f**) and ex vivo (**g**, **h**) experiments, show targeted delivery of the miR-182-5p antagomir to the pancreas. **i** Representative IVIS images from different treatment groups of Pan02 tumor bearing mice injected with vehicle, NC inhibitor, or miR-182-5p encapsulated in a targeted liposomal delivery system targeted to the pancreas. **j** Ultrasound imaging analysis shows significant reduction in tumor volume in mice that received miR-182-5p antagomir treatment as compared to control treatment groups. **k** Pancreatic tumor weight (g) from different treatment groups upon euthanasia. **l** H&E staining of pancreas tissue from tumor-bearing mice from different treatment groups, shows that treatment with miR-182-5p antagomir significantly restored cellular morphology. Non-tumor bearing mouse pancreas was used as healthy control group. **m** Kaplan-Meier survival plots show increased survival in Pan02 tumor bearing mice (*n* = 7 per treatment group) that received miR-182-5p antagomir as compared to vehicle control, NC inhibitor treated. **n** Immunofluorescence staining of pancreas tissue derived from different treatment groups of Pan02 tumor-bearing mice. **o** Flow cytometry data of tumor digests show increased M1/M2 ratio, and CD8^+^ T cell activation markers in 182-5p antagomir treatment group. Data are presented as Mean ± SE, where *n* = 3 per condition, unless specified. ns = *p* > 0.05, * = *p* ≤ 0.05, ** = *p* ≤ 0.01, *** = *p* ≤ 0.001 and **** = *p* ≤ 0.0001 by one-way ANOVA

We asked whether altering the payload of miR182-5p in PaCa-EV cargo would compromise the ability of EVs to skew macrophage plasticity to a pro-tumor phenotype. For this, PPCL-68 cells were treated with miR-182-5p inhibitor and NC inhibitor separately. After 24 h of transfection, cells were washed with PBS, and the media was replaced with basal medium containing 10% (v/v) exosome-depleted FBS. Cells were then allowed to grow for 48 h at the end of which cells and media were collected separately (Fig. 5a). EVs were enriched using SEC from cell culture media (EVs characterization data Supplementary Fig. 7a–d), and miR-182-5p expression was evaluated in cells and in the EVs cargo using qRT-PCR analysis. It was observed that miR-182-5p inhibitor-treated PPCL-68 cells and respective EVs had lower levels of miR-182-5p than NC inhibitor-treated and vehicle-treated cells (Fig. 5b, c). To evaluate potential cytotoxic effects of alterations in miR-182-5p levels in EV cargo on recipient macrophages, THP-1 macrophages were treated with PPCL-68 EVs isolated from different treatment groups (vehicle control, NC inhibitor, and miR-182-5p inhibitor) at a dose of 10 µg/mL. Cytotoxicity was determined by evaluating cell viability using CyQuant cell proliferation (Invitrogen, #C7027), PrestoBlue (Invitrogen, #P50201), as well as XTT assays. All three assays did not show any cytotoxicity (Supplementary Fig. 7e–h). Next, FC analysis of THP-1 macrophages treated with PKH67-labeled PPCL-68 EVs isolated from different treatment groups showed no differences in EV uptake (Supplementary Fig. 7i–k). These data suggest that miR-182-5p reduction in EV cargo does not cause toxicity to macrophages and/or compromise EV uptake by macrophages.

Further, treatment of THP-1 macrophages with PPCL-68 EVs (with a reduced payload of miR-182-5p), resulted in decreased ARG1 and PD-L1 expression as compared to the EVs from NC inhibitor treated or untreated PPCL-68 cells (Fig. 5d). These data demonstrate that reducing the payload of miR-182-5p in PaCa-EV cargo inhibits reprogramming of macrophages to M2-like TAM phenotype, underscoring the translational potential of selectively targeting small RNAs.

### Inhibition of miR-182-5p reduces tumor growth in immunocompetent mice

Based on these compelling data, we posited that targeting miR-182-5p in vivo may alleviate M2-like TAM phenotype, ultimately leading to decreased tumor proliferation. To validate this, Pan02_luciferase cells were orthotopically implanted in C57BL/6 mice, and tumor development was monitored using ultrasound and IVIS imaging. After the first evident signs of tumor development, mice (*n* = 7) were dosed with 75 µg of miR-182-5p inhibitor or the negative control (NC) inhibitor, intravenously, every third day for a total of 30 days (Fig. 5e). A separate cohort of tumor-bearing mice was also dosed with the same volume of transfection reagent as the vehicle control. Initially, we confirmed targeted delivery of miR-182-5p to the mouse pancreas using Cy5.5-labeled miR-182-5p inhibitor mixed with a pancreas-targeted liposomal delivery system (antagomiR-182-5p) injected intravenously into tumor-bearing mice. Employing IVIS imaging at 24 h, we observed miR-182-5p_Cy5.5 signal in the mouse pancreas in vivo (Fig. 5f) and ex vivo (Fig. 5g); however, vehicle-injected mice (control) lacked any detectable signal (Fig. 5f, g). Further, ex vivo analysis of the vital organs in miR-182-5p_Cy5.5 injected mice showed predominant signal from the pancreas, indicating successful delivery to the mouse pancreas and minimal off-target delivery (Fig. 5h). Tumor growth was measured once a week using IVIS imaging and ultrasound; pancreata were weighed at the end of the treatment period. We observed that therapeutic delivery of antagomiR-182-5p resulted in a significant reduction in tumor size, volume, and weight compared to the NC inhibitor and vehicle control groups (Fig. 5i–k). Histopathological analysis of the pancreatic tumor tissue showed increased normal tissue regions in the inhibitor-treated group as compared to the NC inhibitor and vehicle treatment groups (Fig. 5l). Moreover, mice treated with antagomiR-182-5p also showed prolonged survival compared to the mice treated with NC inhibitor and vehicle control (Fig. 5m).

Next, we analyzed tumor samples from all treatment groups for M1 and M2 macrophage markers (CD86 and CD206 respectively), as well as for CD8^+^ T cell infiltration and activation using immunofluorescence and FC analysis (gating strategies in Supplementary Fig. 7l). We found a significant increase in the M1/M2 macrophage ratio in the antagomiR-182-5p treatment group compared to the NC inhibitor and vehicle treatment groups (Fig. 5n, o). These results are of potential clinical significance as numerous studies have shown that an increased M1/M2 ratio is associated with reduced tumor growth and better clinical outcomes across different tumor types.^84–87^ Given that an increased M1/M2 ratio is also associated with the recruitment/activation of CD8^+^ T cells,^88^ we evaluated the total number of CD8^+^ T cells in the pancreatic tumor samples upon antagomiR-182-5p treatment; however, we did not find any significant difference in CD8^+^ T cell numbers among different groups (Fig. 5n, o). Interestingly, the total number of CD8^+^ T cells were found to be significantly higher in the antagomiR-182-5p treatment group when normalized to tumor weight (Supplementary Fig. 7m). Additionally, the number of activated (CD69^+^) and functional (Granzyme B and IFNɣ positive) CD8^+^ T cells were found to be significantly higher in the antagomiR-182-5p treatment group (Fig. 5o). Further, the number of Granzyme B and CD69 double positive CD8^+^ T cells as well as Granzyme B and IFNɣ double positive CD8^+^ T cells were found to be significantly higher in the antagomiR-182-5p treated group (Fig. 5o), suggesting that reprogramming macrophage plasticity by inhibition of exosomal miR-182-5p may serve to activate resident CD8^+^ T cells, thereby enabling efficient tumor cell killing. This is a novel discovery that may have significant therapeutic implications in PaCa research and warrants further in-depth investigation.

**Fig. 6:**
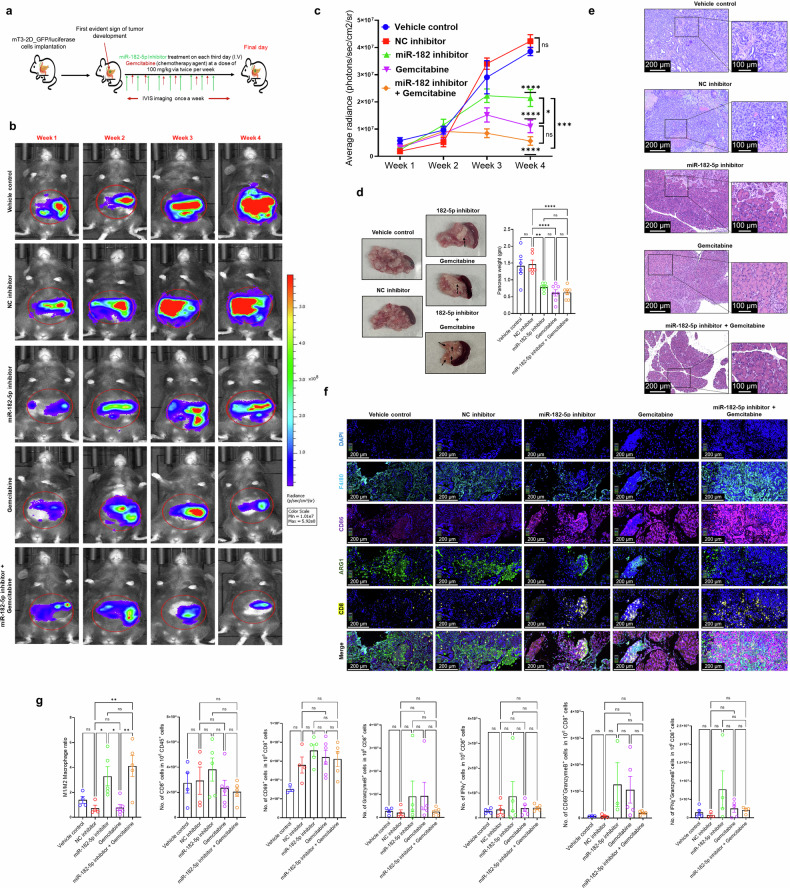
Combinational treatment (miR-182-5p inhibitor + gemcitabine) alleviates mT3-2D tumor burden in immunocompetent mice. **a** Schematic representation of in vivo experiment with mice. **b** Representative IVIS images from different treatment groups of mT3-2D tumor bearing mice injected with vehicle, NC inhibitor, or miR-182-5p inhibitor, gemcitabine and miR-182-5p inhibitor + gemcitabine. **c** ROI analysis of IVIS showing significant reduction in tumor volume in mice that received miR-182-5p antagomir, gemcitabine and miR-182-5p antagomir + gemcitabine treatment as compared to control treatment groups (*n* = 7 per treatment group). **d** Pancreatic tumor weight (g) from different treatment groups upon euthanasia. **e** H&E staining of pancreas tissue from tumor bearing mice from different treatment groups. **f** Immunofluorescence staining of pancreas tissue derived from different treatment groups of mT3-2D tumor-bearing mice. **g** Flow cytometry data of tumor digests showing increased M1/M2 ratio in 182-5p antagomir and miR-182-5p inhibitor + gemcitabine treatment groups. Data are presented as Mean ± SE, where *n* = 3 per condition, unless specified. ns = *p* > 0.05, * = *p* ≤ 0.05, ** = *p* ≤ 0.01, *** = *p* ≤ 0.001 and **** = *p* ≤ 0.0001 by one-way ANOVA

To test the broader applicability of our findings, we conducted an independent study using an aggressive and more clinically relevant mT3-2D tumor model (isolated from the KPC model),^89,90^ which closely recapitulates a human PaCa tumor (Fig. 6a). mT3-2D_GFP/luciferase cells were orthotopically implanted in C57BL/6 mice, and tumor development was monitored using ultrasound and IVIS imaging. After the first evident signs of tumor development, mice (*n* = 7/group) received miR-182-5p inhibitor (75 µg, i.p., every third day), gemcitabine (100 mg/kg, twice weekly for 3 weeks), or both (miR-182-5p inhibitor + gemcitabine); NC inhibitor and vehicle groups served as controls. Tumor growth was quantified weekly by IVIS and pancreatic weights were recorded. Longitudinal IVIS imaging demonstrated progressive reduction in average radiance across all treatment groups as compared to the vehicle control group (Fig. 6b, c). Additionally, IVIS data showed a significantly enhanced anti-tumor response in the cohort that received combination therapy (miR-182-5p inhibitor + gemcitabine) as compared to mice that received miR-182-5p inhibitor or gemcitabine alone (Fig. 6b, c). These findings were consistent with the pancreatic weight as the dissected pancreatic tissue showed decreased tumor burden (weight) in animals receiving miR-182-5p inhibitor, gemcitabine, and combination therapy as compared to vehicle control/NC inhibitor treated groups (Fig. 6d). Histopathological analysis of tumor tissue samples (Fig. 6e) showed that both vehicle control and NC inhibitor-treated groups exhibited completely effaced typical pancreatic microarchitecture characterized by dense malignant epithelial cells with nuclear pleomorphism, mitotic indices, focal necrosis and intense stromal desmoplasia all of which are hallmarks of aggressive PaCa. In contrast, mice from miR-182-5p inhibitor treatment only and gemcitabine treatment only groups showed clear tumor regression characteristics. miR-182-5p inhibitor and gemcitabine treatments alone caused progressive reduction in cellular density with parallel attenuation of nuclear atypia and scattered residual tumor cell nests within fibrotic stroma. Strikingly, combination therapy (miR-182-5p inhibitor + gemcitabine) nearly restored normal parenchymal integrity with well-organized acini and no discernible viable carcinoma. However, some mild stromal fibrosis was still visible. Next, we also evaluated tumor samples from all treatment groups for M1 and M2 macrophage markers, as well as for CD8^+^ T cell infiltration and activation using immunofluorescence and FC analysis. We found a significant increase in the M1/M2 macrophage ratio in the antagomiR-182-5p treatment group and combination (miR-182-5p inhibitor + gemcitabine) therapy group compared to the NC inhibitor and vehicle treatment groups (Fig. 6f, g; gating strategies in Supplementary Fig. 8a, b). Although, we did not find any significant change in CD8^+^ T cell infiltration, we observed an increased trend in CD8^+^ T cell activation markers. Importantly, miR-182-5p inhibition mediated macrophage reprogramming as shown by increased M1/M2 ratio in this aggressive tumor model (Fig. 6g) aligned with our observations with the less aggressive Pan02 tumor model (Fig. 5o). Collectively, these results highlight the efficacy of miR-182-5p inhibition as a viable treatment option for modulating PaCa-TME. The findings also provide a rationale for exploring novel immuno-modulating molecules/drugs alone or in combination with traditional chemotherapeutics for PaCa treatment.

## Discussion

Pancreatic cancer is notoriously resistant to therapy, ultimately leading to the worst prognosis of all solid tumors. TAMs play a pivotal role in creating an immunosuppressive TME, hindering the effectiveness of immunotherapeutic approaches in PaCa. Therefore, strategies aimed at modulating TAM plasticity, including inhibiting their recruitment to the tumor site, reprogramming them towards an anti-tumoral phenotype, and disrupting their interactions with other cell types within the TME, have gained traction for improved therapeutic outcomes in PaCa.^91^ Proliferating tumor cells can utilize various mechanisms of cellular crosstalk to subvert the microenvironment. One of these mechanisms includes cancer-derived EVs that are taken up by surrounding cells, including macrophages, and serve as key mediators of intercellular crosstalk and signal transduction.^2,13^ Previously, we demonstrated that PaCa-EVs induce an unfolded protein response, elevated proliferation, and invasive capabilities in recipient normal pancreatic epithelial cells, underscoring their role in modifying cell function and behavior.^2^ Building on these findings, herein we investigated the effect of PaCa-EVs on phenotype, function, and behavior of macrophages: the most abundant immune cell type in PaCa-TME. We found that the internalization of PaCa-EVs by BMDMs and THP-1 macrophages increased the expression of cell surface markers associated with the M2-like TAM phenotype. Furthermore, we found that PaCa-EV treatment induced secretion of not only anti-inflammatory cytokines such as TGF-β and IL-10 but also pro-inflammatory cytokines, including IL-1β and TNF-α, in BMDMs and THP-1 macrophages, suggesting a mixed phenotype, corroborating previous reports.^22,23,49^

Somewhat unexpectedly, we found that BMDMs and THP-1 macrophages treated with EVs from established and PDX PaCa cell lines showed increased PD-L1 expression. Accordingly, blockade of PD-1/PD-L1 axis using PD-1 and PD-L1 antibodies rescued T cell suppression. These data suggest the potential of targeting TAMs using PD-1/PD-L1 inhibitors to improve anti-tumor therapeutic efficacy. While recent findings in other cancer types have highlighted the induction of PD-L1 expression on macrophages after cancer cell-derived EV treatment, this is a novel finding in PaCa with significant clinical implications.^22,92,93^ Studies in ovarian cancer, colorectal cancer, and melanoma have also revealed that macrophages and other antigen-presenting cells in the TME exhibit significant levels of functional PD-L1.^94,95^

Small RNA sequencing-based characterization of PaCa-EVs derived from established and PDX PaCa cell lines allowed us to identify the crucial role of miR-182-5p as a regulatory mediator of macrophage phenotype in PaCa. Previous studies suggest that miR-182 may contribute to tumor growth, invasion, and metastasis in various cancer types including PaCa.^96–99^ However, this study is among the first to conduct an in-depth investigation into the immunomodulatory effects of miR-182-5p, which is enriched in PaCa-EVs. Furthermore, we observed a significant elevation of miR-182-5p in PaCa-patient plasma EV-cargo, corroborating our findings with established and PDX PaCa cell lines. We also confirmed that human PBMC-derived macrophages treated with patient plasma-derived EVs showed higher expression of TAM markers like *CD206, CD163*, *IL-10*, *TGF-β*, and *STAT3*. Target prediction analysis, as well as subsequent validation studies, suggested that miR-182-5p targets TLR4 at the mRNA and protein levels in PaCa-EV-treated BMDMs. It is well recognized that TLR4 enables macrophages to adopt a pro-inflammatory state by triggering MYD88-dependent NF-κB signaling.^100^ Hence, miR-182-5p-mediated TLR4 downregulation would augment a preferential skewing of macrophages towards an M2-like TAM phenotype through upregulation of an anti-inflammatory state and concomitant activation of JAK/STAT3 signaling. Indeed, we found that treatment of BMDMs and THP-1 macrophages with PaCa-EVs leads to the activation of the JAK2/STAT3 signaling pathway (phosphorylation of JAK2 and STAT3), thereby skewing them towards a pro-tumorigenic M2-like phenotype and enhanced PD-L1 expression on cell surface. Many studies have shown a direct correlation between STAT3 phosphorylation/activation and elevated PD-L1 expression in cells,^71,101,102^ which could, in part, explain the observed increase in PD-L1 expression on macrophages. Separately, we found that PaCa-EV-treated macrophages suppress CD8^+^ T cell proliferation under in vitro and in vivo conditions. Interestingly, pharmacological inhibition of JAK/STAT3 signaling in BMDMs prior to PaCa-EV treatment showed a significant alleviation of CD4^+^ and CD8^+^ T cell suppression. Furthermore, the activation of the JAK/STAT pathway in PaCa-EV educated BMDMs was associated with decreased expression of TLR4, which is known to induce an inflammatory response.^103,104^ While the establishment of precise underlying signaling mechanisms will require further investigations, these findings nonetheless suggest a strong correlation between the JAK/STAT3 signaling pathway and the immunosuppressive phenotype of PaCa-EV-treated macrophages.

Notably, we found that delivery of miR-182-5p to macrophages by PaCa-EVs induced PD-L1 and ARG1 expression, while its inhibition alleviated the M2-like TAM phenotype. Recently, Ma et al. 2022.^77^ have demonstrated the utility of cationized mannan-modified EVs to target miR-182 in macrophages to prevent TAM polarization in breast cancer. The work presented here is among the first to elucidate the role of PaCa-EVs-derived miRNAs that facilitate macrophage programming towards a highly suppressive immune environment in PaCa. Further, using orthotopic, immunocompetent mouse models with varying immunogenic potential, we found that altering the payload of PaCa-derived exosomal miR-182-5p alleviates tumor burden in Pan02 and/or m3T-2D tumor-bearing mice, promotes CD8^+^ T cell activation, and restores M1/M2 ratio within the PaCa TME, thereby improving overall survival. Taken together, these findings conclusively demonstrate that inhibiting miR-182-5p enables macrophage programming towards an anti-tumor phenotype. Regardless, these data do not exclude the direct effect of miR-182-5p inhibition on tumor cells, which warrants an independent investigation. Recently, some groups have reported alleviation of TAM polarization by inhibiting cancer cell EV release in a bladder cancer model, which may cause cellular toxicity.^11^ We present an alternative paradigm that relies on specifically targeting components of PaCa-EV cargo to abrogate crosstalk with surrounding macrophages, thus alleviating immunosuppressive TME. While these studies unequivocally establish the role of cancer cell-derived exosomal miR-182-5p in potentiating an M2-like TAM phenotype in PaCa, further studies will be crucial to define the reciprocal role of TAM-EVs in promoting cellular crosstalk with tumor cells and CAFs within the TME. Future studies also need to delineate the synergistic interaction of other components of EV cargo, including miRNAs, proteins, and small molecules that can then be targeted for abrogating cellular crosstalk conducive to tumor growth.

## Methods

### Cell lines and cell culture

All established human pancreatic cancer (PaCa) cell lines: PANC-1 (CRL-1469), Capan-1 (HTB-79), MIA PaCa-2 (CRM-CRL-1420), and SW-1990 (CRL-2172) were procured from American Type Culture Collection (ATCC) and maintained/cultured in modified minimum essential medium (MEM) (Gibco, #A1048801) containing 10% (v/v) HI-FBS (Gibco, #10082147) and 1% Penicillin-Streptomycin (Gibco, #15140122). Similarly, the non-tumorigenic pancreatic epithelial cell line hTERT-HPNE was also purchased from ATCC and cultured in keratinocyte serum-free medium supplemented with bovine pituitary extract, human recombinant epidermal growth factor (Gibco, #37010022), and 1% (v/v) Penicillin-Streptomycin (Gibco, #15140122). The patient-derived xenograft PDAC cell lines (PPCL-46 and PPCL-68) were a gift from Dr. Trevino (School of Medicine Surgery, Virginia Commonwealth University) and were grown in Advanced MEM medium containing 10% (v/v) HI-FBS, 5 mM L-Glutamine (Gibco, #25030081), and 1% Penicillin-Streptomycin. The human monocytic cell line THP-1 was procured from the ATCC and cultured in RPMI-1640 (ATCC, #30-2001) containing 10% (v/v) FBS (Gibco, #10082147) and 1% Penicillin-Streptomycin (Gibco, #15140122). The murine pancreatic cancer cell line Pan02_luciferase was procured from the Vitro Biotech (VLU5B084) and cultured in RPMI-1640 medium (Corning, #100-041-CV) containing 10% (v/v) HI-FBS (Gibco, #10082147), 1% Penicillin-Streptomycin (Gibco, #15140122), and 100 ng/ml Puromycin (Gibco, #A11138-03). The murine PaCa cell line, mT3-2D was a gift from Dr. David A Tuveson (Cold Spring Harbor Laboratory, Cold Spring Harbor, NY), the GFP/luciferase-expressing mT3-2D cells were provided by Dr. Chunling Yi (Georgetown University) and mT3-2D was grown in DMEM medium containing 10% (v/v) HI-FBS, 5 mM L-Glutamine (Gibco, #25030081), and 1% Penicillin-Streptomycin. All the cell lines were maintained at 37 °C with 5% CO_2_.

### Mice

Wild-type (WT) C57BL/6, *Rag1 KO*, and pmel-1 female mice (4-6 weeks old) were purchased from The Jackson Laboratory and housed under a pathogen-free environment at Georgetown University Medical Center animal facility. All animal experiments were approved by the Institutional Animal Care and Use Committee (IACUC), Georgetown University, under animal protocol numbers 2022-0042 and 2023-0027.

For in vivo EVs uptake experiments for analyzing the effect of PaCa-EVs on the PD-L1 expression in peritoneal cavity resident macrophages (PCMs); C57BL/6 mice were intraperitoneally injected with EVs at a dose of 100 µg/mice in a total volume of 100 µL. At 72 h post EV injections, the PCMs were harvested. The mice were euthanized using CO_2_ confirmed via cervical dislocation and placed on their backs on a paper towel in a biosafety cabinet. The abdomen was disinfected with 70% alcohol, and a small incision was made in the skin to expose the peritoneal wall. The skin was gently pulled back to expose the peritoneal membrane, and a needle was carefully inserted, avoiding internal organs. Nearly 5 ml of ice-cold PBS was injected into the peritoneal cavity, and the abdomen was massaged for 10–15 s before withdrawing the needle. Next, a fresh 20 G needle was inserted into the peritoneal cavity, and maximum amount of fluid was withdrawn. The fluid was collected into a 15 mL centrifuge tube, kept on ice, and centrifuged at 1,800 rpm for 5 min to collect the cell pellet. The supernatant was discarded, and the harvested cells were processed for the flow cytometry analysis.

### Adoptive cell Transfer (ACT) experiments

Murine splenic CD8^+^ T cells were isolated from the spleens of the pMel-1 mice using MojoSort Mouse CD8^+^ T cell Isolation Kits (BioLegend, # 480008) following the manufacturer’s instructions. The cells were cultured in 6-well plates at a concentration of 1 × 10^6^ cells/mL in complete RPMI-1640 medium supplemented with recombinant murine IL-2 [20 ng/mL] and activated using gp100 peptide (KVPRNQDWL; 1 µM/10^6^ cells/mL). After 48 h, cells were harvested, washed with ice-cold PBS, and resuspended in fresh PBS to a concentration of 10 × 10^6^ cells/mL. For ACT experiments, these cells were infused (day 0) into the *Rag1 KO* mice (1 × 10^6 ^T cells/mice) using tail vein injections. Next day (day 1), the recipient mice were injected with a subcutaneous injection of gp100 peptide. At day 2, the mice were infused with 0.5 × 10^6^ macrophages that had been pretreated with or without EVs. Six days after infusion of CD8^+^ T cells; blood and spleen were collected upon euthanasia and analyzed for CD8^+^ T cell number, proliferation, and function via flow cytometry.

### Orthotopic pancreatic cancer model development and studies

6-8 weeks old female C57BL/6 mice were used for orthotopic pancreatic tumor model development as per the previously established protocol with slight modifications.^105,106^ Briefly, the animals under isoflurane anesthesia were positioned on the VEVO 3100 Imaging Station. Next, Pan02_luciferase cells (1 × 10^6^) or mT3-2D_GFP/luciferase cells (1 × 10^6^) suspended in 1:1 Matrigel (Corning, #356237) and PBS (total volume of 50 µL) were directly injected into the mice pancreas using high-resolution ultrasound-guided injections. Tumorigenesis was assessed weekly post-implantation using both IVIS analysis (IVIS Lumina III Imaging System, PerkinElmer) and ultrasound imaging. For the in vivo bioluminescence analysis, the mice were intraperitoneally injected with IVISbrite D-Luciferin (Revvity, #122799) 15 min before imaging (as per the luciferin kinetic curve analysis). At the first evident signs of tumor growth (day 28), the mice were randomly divided into different treatment groups. Vehicle, negative control (NC) inhibitor, and miR-182-5p inhibitor (Horizon Discovery) were administered every third day via tail vein at doses suggested by the PANCREAS-targeted In Vivo Transfection Reagent (Altogen Biosystems, #5051) manufacturer’s recommendation (details given under method details**)**. A group of negative control mice without any cancer cell inoculation were also maintained which received only PBS I.V. injections. Both IVIS and ultrasound imaging were continued regularly for tracking tumor growth. Following the IACUC guidelines, individual animals were euthanized once the tumor volume reached a maximum of 1.5 cm^3^ or the animal lost more than 20% of its body weight. On the day of sacrifice, the tumor tissues as well as normal pancreas were carefully isolated, weighed, and washed. Tissue sections were fixed in 4% formalin (Fisher Brand, #245-684) as well as dissociated for flow cytometry analysis as per the method details.

### In vivo targeting and biodistribution of miR-182-5p-lipoplexes

At day 28 post-implantation of luciferase expressing Pan02 (1 × 10^6^) cells, the pancreas targeted liposomes loaded Cy5.5 labeled miR182-5p inhibitor (Horizon Discovery) were administered in tumor-bearing animals via tail vein injection. 24 h later, the mice were subjected to IVIS imaging for biodistribution analysis. Subsequently, the mice were euthanized, and major organs were excised to record the corresponding fluorescence signal.

### EV isolation and characterization

EVs were isolated from the conditioned media using size exclusion chromatography (SEC).^2^ When the cells reached 50-60% confluency, the media was discarded and the cells were washed with PBS. The media for each cancer cell line was replenished with their respective basal media containing 10% (v/v) exosome-depleted FBS (Gibco, A2720801) and 1% (v/v) Penicillin-Streptomycin (Gibco, #15140122). For hTERT-HPNE cells, the medium was replaced with fresh serum-free medium (K-SFM) supplemented with bovine pituitary extract, human recombinant epidermal growth factor (Gibco, #37010022), and 1% (v/v) Penicillin-Streptomycin (Gibco, #15140122). Cells were grown for 48 h. Subsequently, the media was collected and centrifuged twice at 2,500 × g for 10 min at 25 °C to remove any debris or cells. The supernatants were then concentrated using 100 kDa Centricon Plus-70 filters (Millipore, #UFC710008) by spinning at 3500 × g for 20 min at 25 °C. Filtered flow-through was discarded, and the concentrate was collected as per the manufacturer’s instructions. The process was repeated until all the supernatant was concentrated. The EVs were isolated from the concentrate via SEC by utilizing a qEV2/70 nm column (Izon Science, IC270) with an automated fraction collector, according to the manufacturer’s protocol. The first three fractions were pooled, lyophilized, and resuspended in DPBS and stored in −80 °C until further characterization and experiments.

### Human plasma sample collection and Isolation of plasma EVs

The biospecimen collection study was approved by Georgetown University-MedStar Health Institutional Review board under protocol IRB# 2007-345. Patient and healthy donors signed the information consent. Plasma EVs were isolated using SEC. Briefly, plasma samples were thawed on ice, and 100 µL of plasma was diluted in 500 µL of 1X PBS. Samples were centrifuged at 14,000 rpm for 20 min at 4 °C to remove any particulates. The supernatant was then transferred to a 100 kDa Amicon Ultra-0.5 centrifugal filter unit (UFC5100) and centrifuged at 14,000 rpm for 5 min at room temperature. Concentrate is collected and loaded on Izon qEV single SEC column (Izon qEV Single: SP2, 70 nm, 150 µL capacity) on a fraction collector as per the manufacturer’s protocol. The EV fractions were collected, combined, freeze-dried, and reconstituted in DPBS and stored in −80 °C until further used.

### Nanoparticle tracking analysis (NTA)

NTA was conducted using a NanoSight NS300 system (Malvern Panalytical) outfitted with a high-precision sCMOS camera, a 531 nm laser, and an automated syringe pump. Each sample was diluted 1:1000 in HPLC-grade water before being loaded into the automated syringe injector. The camera level was set to 12, the detection threshold was set to 10, and the syringe pump was set to a speed of 20. The videos were captured (30 s time frame x 3) and analyzed using NTA 3.3 Dev Build 3.3.10 software to determine the size distribution and particle concentration.

### Protein concentration estimation

The protein concentration was estimated using the bicinchoninic acid (BCA) protein assay kit (ThermoFisher Scientific, #23225) as per the manufacturer’s instructions. Bovine serum albumin (BSA) was used as a standard, and absorbance was measured at 562 nm to generate a standard curve. The protein concentration of the samples was then estimated from the standard curve.

### Immunoblot analysis for EV marker proteins

To analyze the expression level of EV-associated proteins, immunoblots were performed using Exo-Check Exosome Antibody Array (System Biosciences, #EXORAY210A or #EXORAY210B) kit as per the manufacturer’s instructions. The protein concentration of the EVs was measured using a (BCA) protein assay kit (Thermo Fisher, #23225). The blots were developed using SuperSignal West Pico PLUS Chemiluminescent Substrate (ThermoFisher Scientific, #34577) and then imaged with the Amersham Imager 600 (GE Healthcare Life Sciences). (Note: For healthy donor and PDAC patient plasma-derived EVs, 5 patient samples were pooled to perform Immunoblot analysis for EV marker proteins).

### Cryogenic transmission electron microscopy (cryoTEM)

EV samples ( ~ 3.5 μL) were loaded onto glow-discharged, perforated carbon-coated grids (2/1-3C C-Flat; Protochips, Raleigh, NC), blotted with filter paper, and rapidly plunged into liquid ethane. These grids were stored in liquid nitrogen. Before imaging, the grids were placed on cryo-specimen holder (626 cryo-specimen holder, Gatan, Warrendale, PA) and maintained at −180 °C. The digital micrographs were captured using a Tecnai F20 Twin transmission electron microscope (ThermoFisher Scientific, Hillsboro, OR) equipped with a Gatan US4000 CCD or a Teitz XF416 camera.

### Labeling of EV by PKH67

EVs were labeled with the green fluorescent dye PKH67 (Sigma, #PKH67-GL-1KT) as per the manufacturer’s instructions. A 2x dye solution was prepared in diluent C (1 ml) by adding 4 µL of the PKH67 dye solution. Equal volumes of EV stock solution and 2x dye were mixed to get a final dye concentration of 1x and incubated at room temperature for 5 min in the dark, followed by the addition of FBS to neutralize the dye. Unbound dye was removed using 3 kDa MWCO centrifugal filters (Invitrogen, #448449) according to the manufacturer’s protocol. For the negative control, 1x DPBS (without EVs) was mixed with PKH67 dye and processed in the same way.

### Generation of murine bone marrow derived macrophages (BMDMs)

BMDMs were generated according to previously described methods.^107^ Briefly, bone marrow cells from the mice femur and tibia were cultured in RPMI-1640 (Corning, #100-041-CV), supplemented with 10% HI-FBS, 1% Penicillin-Streptomycin, and 25 ng/mL of recombinant murine macrophage-colony stimulating factor (M-CSF) (BioLegend, #576404). Nearly 7 × 10^6^ cells were seeded into 100 mm petri dishes (10 mL medium). On day 3, the same amount of medium (10 mL) with 25 ng/mL M-CSF was added to the culture plates. On day 5, the culture medium was discarded, and the attached cells were washed with DPBS and allowed to rest in RPMI-1640. BMDMs were harvested on day 6 and seeded for subsequent experiments.

### Generation of THP-1 macrophages

THP-1 monocytes were differentiated into macrophages according to the reported protocol^108^ with some modifications. THP-1 monocytes (1 × 10^6^ cells/mL) were cultured in complete growth media containing 100 nM phorbol myristate acetate (PMA) (Millipore, #52440010MG) overnight, followed by PBS wash. After resting 72 h in fresh complete growth media, THP-1 macrophages were treated with respective EVs for 72 h and subjected to downstream experiments.

### Generation of human PBMCs-derived macrophages

Upon receipt, buffy coat bags (Oklahoma Blood Institute (https://ourbloodinstitute.org/) were removed from the shipping container and gently rocked at room temperature for 30 minutes to 1 h. During this time, the centrifuge was pre-warmed to room temperature. After rocking, each bag was carefully opened, and approximately 40–45 mL of buffy coat was drained into a 50 mL conical tube. The sample was then split evenly into two 50 mL conical tubes (~20–22 mL each). For PBMC isolation, 15 mL of density gradient medium was added to each SepMate™ tube by gently pipetting through the central hole of the insert. The buffy coat was diluted 1:1 with PBS supplemented with 2% FBS, mixed thoroughly, and 35 mL of the diluted sample was carefully added down the side of each SepMate™ tube. A serological pipette was used at a low speed to avoid disturbing the gradient. Tubes were centrifuged at 1200 × g for 20 min at room temperature. The upper layer, containing the enriched PBMCs, appeared cloudy and yellowish-white and was carefully aspirated into a new tube. Cells were pelleted by centrifugation at 300 × g for 8 minutes at room temperature, then washed with PBS + 2% FBS. This wash step was repeated once. The final pellet was resuspended either in PBS + 2% FBS + 1 mM EDTA for monocyte isolation or in high-serum FBS (Hi FBS) for cryopreservation. Cell counting was performed prior to downstream applications. Further, for monocyte isolation, PBMCs were diluted to a concentration of 5 × 10⁷ cells/mL, ensuring the total volume did not exceed 2 mL. The diluted sample was transferred into a 5 mL polystyrene round-bottom tube. An isolation cocktail was added at a volume of 50 µL per mL of sample, followed by the addition of a platelet removal cocktail at the same ratio (50 µL/mL). The mixture was gently pipetted to mix and incubated at room temperature for 5 minutes without a cap. Magnetic particles were vortexed for 30 seconds and then added to the sample at 50 µL per mL. The mixture was again gently pipetted and incubated for 5 minutes at room temperature, uncapped. The sample was then diluted to a final volume of 2.5 mL using PBS supplemented with 2% FBS and 1 mM EDTA. After gentle mixing, the tube was placed in a magnetic separator and incubated at room temperature for 2.5 minutes, uncapped. The enriched monocyte fraction was then collected by inverting the magnet and tube into a fresh 5 mL or 15 mL tube. The enriched cells were centrifuged at 200 × g for 10 min at 4 °C. The monocyte pellet was resuspended in ImmunoCult™-SF macrophage medium (Stem cell technologies #10961) and differentiated into macrophages as using human M-CSF as per the manufacturer’s recommendations.

### EV uptake analysis by confocal microscopy

Nearly 10,000 BMDMs / THP-1 macrophages were seeded on a 13 mm glass-bottom petri dish and allowed to grow overnight. The next day, the EVs were labeled with PKH67 as mentioned above (Labeling of EVs by PKH67) and co-cultured with the BMDMs at a concentration of 10 µg/mL for 24 h. Prior to imaging, cell membranes were stained with CellMask Deep Red (ThermoFisher Scientific, #C10046), followed by endoplasmic reticulum staining with ER-Tracker Red (ThermoFisher Scientific, #E34250) as per the manufacturer’s instructions, respectively. The nuclei were stained with Hoechst stain (Invitrogen, #33342) as per manufacturer’s instructions. BMDMs that were treated with a mixture of DPBS and dye solution served as a negative control. All images were captured at 63x using a confocal laser microscope (Zeiss Axio Observer 7, LCM700) and processed using the Zen 3.5 software (Zeiss corporation). IMARIS (Oxford Instruments) was used to make 3D-rendered images and videos of acquired Z-stacks.

### EV uptake estimation by flow cytometry

Nearly 100,000 macrophages (murine BMDMs) were seeded in a 24-well cell culture plate and allowed to grow overnight (for THP-1 macrophages, 200,000-300,000 cells were seeded in a 12-well cell culture plate and allowed to grow overnight). Next day, the EVs were labeled with PKH67 as mentioned above (Labeling of EVs by PKH67) and used to treat the macrophages at a concentration of 10 µg/mL. The cells were harvested at 3 h, 6 h, 12 h, and 24 h post-EV treatment. After DPBS wash, the cells were transferred to flow cytometry tubes and stained for live/dead staining (SYTOX™ Blue Dead Cell Stain, ThermoFisher Scientific, #S34857). Flow cytometry data was acquired using the BD LSR Fortessa platform (BD Biosciences). The results were analyzed using FlowJo software versions 9 and 10 (FlowJo, LLC).

### Flow cytometry analysis

The cells were harvested, stained with Live/Dead (Fixable near-IR cell) staining dye (Invitrogen, #34976 A), and then fixed using fixation buffer (BD Pharmingen, #51-9006124) as per the manufacturer’s protocol. For surface markers, the cells were stained with antibodies (reagent table) diluted in 1% BSA solution prepared in PBS for 45 mins at 4 °C in the dark, then washed twice with PBS. For assessment of intracellular proteins, cells were fixed using Mouse Foxp3 Fixation Buffer (BD Pharmingen, #51-9006124), permeabilized using BD Perm/Wash buffer (BD Biosciences, #51-2091KG) for 30 mins at 37 °C, and incubated with antibodies for 1 h at room temperature. After a PBS wash, cells were resuspended in PBS, and data was acquired using the BD LSR Fortessa platform (BD Biosciences). The results were analyzed using FlowJo software versions 9 and 10 (FlowJo, LLC) and FCS Express 7 (https://denovosoftware.com/.)The cells treated with PBS served as the negative control for the EV treated samples.

### Immunofluorescence (IF)

Approximately 5000 BMDMs were seeded in a 96-well glass-bottom plate and incubated overnight. The next day, the old media was replaced with fresh RPMI-1640 medium (Corning, #100-041-CV) containing 10% (v/v) exosome-depleted FBS (Gibco, A2720801) and 1% Penicillin-Streptomycin (Gibco, #15140122) and treated with EV at a dose of 10 µg/mL. After 72 h, media was removed, cells were washed with PBS, fixed with methanol, and permeabilized with 0.1% Triton-X100 for 15 mins. Afterwards, cells were blocked in 3% BSA for 15 mins and were incubated with appropriate primary antibodies (CD206, Proteintech, #60143-1 g, and PD-L1, Proteintech, #66248-1 g) overnight at 4 °C. The next day, the cells were gently washed with DPBS and incubated with Alexa Fluor 647 (Invitrogen., A32733) or Alexa Fluor 488 (Invitrogen., A32731TR) secondary antibodies, respectively, for 1 h at room temperature. Cells were then stained with DAPI, and images were captured using the Keyence BZ-X710 inverted fluorescent microscope (Keyence corporation).

### Cytokine estimation

Macrophages (BMDMs and THP-1) were seeded in 12-well plates, followed by 10 µg of EV treatment for 72 h. The supernatant was collected, centrifuged at 10,000 rpm for 15 mins at 4 °C, and the supernatant was collected and subjected to the cytokine estimation by using LEGENDplex™ Mouse/Human Macrophage/Microglia Panel (13-plex) (BioLegend, # 740845 and #740930) as per the manufacturer’s instructions. The data was acquired using BD LSR Fortessa platform (BD Biosciences). The data were analyzed using LEGENDplex™ data analysis software.

### Western blot analysis

Total protein was collected by lysing cells (BMDMs or THP-1 macrophages) with Mammalian Protein Extraction Reagent (M-PER) (ThermoFisher, #7850). The protein quantity in lysate was estimated using a BCA protein assay kit (Thermo Fisher, #23225), and 20-25 μg of protein per sample was subjected to electrophoresis on Bolt™ Bis-Tris Plus 4-12% Mini Protein Gels (Thermo Fisher, #NW04120BOX) at 150 V and was blotted to PVDF membranes using an iBlot transfer device (Invitrogen). The membrane was blocked with 3% bovine serum albumin (BSA) in 1x tris buffer saline (TBS) for 1 h, followed by the addition of primary antibody (all antibody details are provided in the reagent table), and membranes were incubated at 4 °C overnight with constant shaking. The next day, membranes were washed 3 times (5 mins each) with tris buffer saline containing 0.1% (v/v) Tween-20 (TBST) and incubated with secondary antibodies for 1 h at room temperature. Then the membranes were washed 3 times with TBST. The blots were developed using SuperSignal West Pico PLUS Chemiluminescent Substrate (ThermoFisher Scientific, #34577), and images were captured using an Amersham Imager 600 (GE Healthcare Life Sciences).

### Targeted metabolomic profiling

Cell samples (200,000 BMDMS/well) were mixed with 300 µL of methanol/water (1:1) containing internal standards (200 ng/mL of debrisoquine for positive mode and 200 ng/mL of 4-nitrobenzoic acid for negative mode), followed by gentle scraping for 20 s. The samples were transferred to 2 mL Eppendorf tubes. To the 24-well plate, 200 µL of PBS was added, mixed well, and then transferred to the cell lysate collected previously. The samples collected were then freeze dried (lyophilized). The lyophilized powdered samples were suspended in 25 μL of PBS and vortexed gently for 30 s. Samples were plunged into dry ice for 30 s and heat shocked by plunging into a 37 °C water bath for 90 s. This was repeated a total of three times. Samples were then sonicated for 30 s. Next, 100 μL of methanol/water (1:1) was added to the samples. Tubes were vortexed for 30 s, incubated on ice for 20 mins, followed by the addition of 100 μL of chilled ACN. The samples were incubated at −20 °C for 20 mins. Finally, samples were centrifuged at 13,000 rpm for 20 mins at 4 °C. Supernatant was transferred to an MS vials for LC-MS analysis. Mass spectrometry data acquisition were performed using QTRAP® 7500 LC-MS/MS System (Sciex, MA, USA). Details are provided in the Supplementary Document.

### Mass spectrometry sample preparation for media

500 µL of media collected from each sample was freeze-dried (lyophilized) and suspended in 50 µL of PBS, followed by vortexing for 30 s. To the above mixture, 100 μL of extraction buffer (methanol/water 50/50) containing 200 ng/mL of debrisoquine (DBQ) as internal standard for positive mode and 200 ng/mL of 4-nitrobenzoic acid as internal standard for negative mode, was added. The sample was vortexed for 30 s and incubated on ice for 20 mins, followed by the addition of 100 μL of acetonitrile and incubation at -20 °C for 20 mins. Samples were centrifuged at 13,000 rpm for 20 mins at 4 °C. The supernatant was transferred to MS vial for LC-MS analysis. Mass spectrometry data acquisition were performed using QTRAP® 7500 LC-MS/MS System (Sciex, MA, USA). Details are given in the Supplementary Document.

### In vitro T cell proliferation/suppression assay

C57BL/6 splenic CD3^+^ T cells or CD8^+^ T cells were isolated by using MojoSort Mouse CD3^+^ T (BioLegend, #480024) and CD8^+^ T cells (BioLegend, #480008) Isolation Kits, respectively. After cell counting, the cells were labeled with Cell Trace Violet (CTV) dye (Invitrogen, #C34557) according to the manufacturer’s protocol. Labelled CD3^+^ T or CD8^+^ T cells were plated as 0.8×105 cells per 100 µL/well in RPMI-1640 media (Corning, #100-041-CV) supplemented with 10% HI-FBS, 10 mM sodium pyruvate, 1% Penicillin/Streptomycin, 0.1% and interleukin-2 (IL-2, 100 U/mL) (Gibco, # 21212100UG) in a U-bottom 96-well plate with Dynabeads Mouse T-Activator CD3/CD28 beads (Invitrogen, #11453D) for T cell expansion and activation. Subsequently, EV-educated macrophages and untreated macrophages were resuspended in complete medium and co-cultured with CD3^+^ T or CD8^+^ T cells to get 1:0.25, 1:0.5, 1:1, and 1:2 T cells to macrophage ratios in a final volume of 200 µL/well. Labelled CD8^+^ T cells, cultured without mouse CD3/CD28 beads, were also seeded as a no proliferation control (only IL-2 treatment group without T-Activator CD3/CD28 beads). After 72 h, the cells were harvested, stained with Live/Dead Fixable near-IR cell staining dye (Invitrogen, Cat No. 34976 A), anti-CD4 and anti-CD8 antibodies. The data was acquired using BD LSR Fortessa platform (BD Biosciences). The results were analyzed using FlowJo software versions 9 and 10 (FlowJo, LLC).

Contact-independent suppression assay was also performed in the same way except that a 0.4 µm size transwell was placed between CD8^+^ T cells and macrophages to inhibit their contact. For anti-PD-1 (invivoMAb, #BE0033-2), anti-PD-L1 (Invitrogen, #16-5982-85), anti-TGF-β (Invitrogen, #16-9243-85), and anti-IL-10 (Invitrogen, # MA5-23796) blockade experiments, these antibody blockades were added at a concentration of 25 μg/mL in each well. For the JAK/STAT3 inhibition experiments, BMDMs were treated with 10 nM of ruxolitinib (RUXO) (Selleck Chemicals, S1378) 2 h before EV treatment.

### MicroRNA sequencing and data analysis

MicroRNA was isolated from hTERT-HPNE, PANC-1, and PPCL-68 cell line-derived EVs using Invitrogen™ *mir*Vana™ miRNA Isolation Kit (Invitrogen, #AM1561), according to the manufacturer’s instructions. Library preparation and sequencing were performed by Novogene Corporation (Sacramento, CA) using the NEBNext^®^ Small RNA Library Prep Set for Illumina^®^ (New England Biolabs). 3’ and 5’ adaptors were ligated to the 3’ and 5’ ends of small RNA, respectively. The first strand cDNA was then synthesized after hybridization with a reverse transcription primer. The double-stranded cDNA library was generated through PCR enrichment. After purification and size selection, libraries with insertions between 18 ~ 40 bp were ready for sequencing with SE50. The library was checked with Qubit and real-time PCR for quantification and a bioanalyzer for size distribution detection. Quantified libraries were pooled and sequenced using NovaSeq 6000. The data provided in.bam files were used for subsequent analysis using Partek Genomics Suite 6.6 (PGS, Partek Genomics Suite software, version 6.6 beta, Partek Inc., St. Louis, MO, USA). To determine the differences among non-tumorigenic cell line (hTERT-HPNE) derived and PaCa (PANC-1 and PPCL-68) derived EVs, ANOVA was performed with the threshold of *p* ≤ 0.05 to filter out significant differences in the differential expression of miRNA.

### qRT-PCR analysis for miRNAs expression

Total RNA was extracted using the Invitrogen™ *mir*Vana™ miRNA Isolation Kit (Invitrogen, #AM1561) according to the manufacturer’s protocol. In all cases, samples were spiked with 100 nmol of artificial *Caenorhabditis elegans* (*C. elegans*) cel-miR-54 (5’-UACCCGUAAUCUUCAUAAUCCGAG-3’) (Integrated DNA Technologies (IDT, #408324284) during RNA extraction to serve as an exogenous control. RNA quantity was determined by NanoDrop spectrophotometry. Reverse transcription of cDNA was performed using TaqMan Advanced miRNA cDNA (Invitrogen, # A280007) synthesis kit according to the manufacturer’s instructions. qRT-PCR was run on StepOne Plus and QuantStudio 7 Flex instruments (Applied Biosystems) by using TaqMan assays as per the manufacturer’s protocol (ThermoFisher Scientific). Relative quantification was used, and the levels of targeted microRNAs (miR-182-5p, miR-106b-3p, miR-7-5p, and miR-9-5p) were normalized to the level of reference cel-miR-54.

### qRT-PCR analysis for mRNA expression

Total RNA was extracted using the Norgen Total RNA Isolation Kit (Norgen Biotek, #17200) according to the manufacturer’s protocol. Total cDNA synthesis was performed using TaqMan reverse transcription kit (Invitrogen, # N8080234) and TaqMan assays, as per manufacturer’s protocols (ThermoFisher Scientific). GAPDH was used as a housekeeping gene, and qRT-PCR was run on StepOne Plus or QuantStudio7 Flex instruments (Applied Biosystems).

### Cell viability or cytotoxicity analysis

Approximately 5000 THP-1 macrophages were seeded in 96 well plate for overnight, and the next day treated with PPCL-68 EVs isolated from different treatment groups (vehicle control, NC inhibitor, and miR-182-5p inhibitor) at a dose of 10 µg/mL. EVs cytotoxicity is analyzed by evaluating the cell viability using CyQuant cell proliferation (Invitrogen, #C7027), PrestoBlue (Invitrogen, #P50201), as well as XTT assays (Invitrogen, #X‘12223) as per the manufacturer’s instructions. The Untreated cells were used as the normal proliferation control.

### Transfection of miR-182-5p mimic/inhibitor

BMDMs, THP-1 macrophages, and PPCL-68 cells were seeded in their respective culture media to attain a confluency of 60–70%. The miR-182-5p mimic and inhibitor (human miR-182-5p mimic and inhibitor were used for human cell lines, and murine miR-182-5p mimic and inhibitors were used for murine cells) were transfected at a concentration of 30 pmol, along with their respective negative controls (NC), using Lipofectamine RNAiMAX (Invitrogen, #13778075) as per the manufacturer’s protocols. After 24 h and 48 h, cells were harvested for RNA and protein extractions. The transfection was confirmed by estimating miR182-5p expression using qRT-PCR or by analyzing the target protein expression using western blot.

### RNA-sequencing

BMDMs were treated with hTERT-HPNE, PANC-1, and PPCL-68 cell line-derived EVs for 48 h. Total RNA was isolated using the Norgen Total RNA Isolation Kit (Norgen Biotek, #17200) according to the manufacturers protocol. RNA quality control (QC) was assessed using Qubit (Thermo Fisher) and Bioanalyzer (Agilent) analysis. Next, the total RNA was analyzed using high through put RNA- Seq Illumina Platform as per the manufactures’ instructions. The differential gene expression was identified with respect to the control treatment group. The targets of interest were validated by using qRT-PCR.

### Loading of murine miR-182-5p inhibitor in liposomes for in vivo studies

The synthetic miRNA inhibitors (NC and miR-182-5p inhibitor (Horizon Discovery) were loaded into pancreas-targeted liposomes **(**Pancreas In Vivo Transfection Reagent, Altogen Biosystems, #5052). 100 µL of diluted RNA (1 µg/µL RNase-free water) was mixed with 50 µL transfection reagent and incubated for 15 min. Subsequently, 10 µL of transfection enhancer reagent was added and kept for 5 min at room temperature (RT). Glucose (5%, w/v) isotonic solution was used to finalize the injection volume to 0.2 mL per mouse. The miRNA inhibitor doses were administered as per the manufacturer’s instructions using tail vein and intraperitoneal injections.

### Histology

On the day of euthanasia, the tumor tissues, as well as normal pancreas, were carefully isolated, weighed, washed, and fixed in 4% formalin (Fisher HealthCare, #23-305510) for 48 h. The tissue sections were then transferred to 70% ethanol and dehydrated using gradient ethanol concentrations, followed by paraffin embedding. Tissue-embedded blocks were processed in 2 μm sections and stained with eosin and hematoxylin according to standard protocols for histopathological analysis.

### Multiplex immunofluorescence staining (MIFs)

MIFs were performed on the formalin-fixed, paraffin-embedded tissue samples. The sections were sequentially stained for pan-CK (Agilent, #M3515), CD11b (Abcam, #ab209970), F4/80 (eBioscience, #14-481-85), CD86 (Abcam, #ab220188), ARG1 (ThermoFisher Scientific, #PA5-29645), and CD8 (Cell Signaling Technology, #98941) using Opal fluorescent dyes (Akoya) following the manufacturer’s instructions. All slides were deparaffinized, rehydrated, and then boiled in antigen retrieval solution consisting of either citrate buffer (pH 6.0) or EDTA buffer for the respective primary antibodies. The sections were blocked using antibody diluent/blocking buffer (Akoya, #ARD1001EA), incubated with the primary antibodies at room temperature, and then incubated with Opal Polymer HRP anti–mouse/rabbit IgGs before incubation with the corresponding Opal fluorophores (OPAL 480, 520, 570, 620, 690, and 780). The slides were counterstained using DAPI and sealed in Prolong Gold (ThermoFisher Scientific). The whole sections were scanned using Phenofusion Imager (Akoya). Representative fields from the single-color slides were analyzed using InForm software v2.2.0 (Akoya) to generate a spectral library. Index cases stained using the multiplex method were then imaged. Channels were unmixed using the spectral library, and the whole tissue sections were segmented, quantified, and normalized to the tissue area.

### Tumor dissociation for flow cytometry analysis

After harvesting, tissue samples were weighed and mechanically dissociated in ice-cold PBS. Cells were isolated after filtration through a 70 nm cell strainer, and centrifuged at 1800 rpm for 5 min at 4 °C. Isolated cells were then frozen for later use in FBS containing 10% DMSO. At the time of use, cells were thawed and stained for flow cytometry, and data were acquired using the BD LSR Fortessa platform (BD Biosciences) or Cytek (cytekbio.com). The results were analyzed using FlowJo software versions 9 and 10 (FlowJo, LLC).

### Reagents used

Details of all the regents and resources used in the present investigation are provided under Supplementary Table 4.

### Statistical analysis and software

GraphPad Prism was used to prepare all the graphs and perform the statistical analysis. All the data are represented as mean ± standard error of the mean (SEM). Information for the statistical test used for each analysis is provided in the figure legends. ImageJ was used to quantify Western blots and immunofluorescence signals. R (version 4.2.2) (https://cran.r-project.org/) was used to generate the heatmap and PCA plots. BioRender (https://www.biorender.com/) was used to draw the images of all schematics. Kaplan-Meier Plotter (https://kmplot.com/analysis/) was used to plot the survival curve analysis for pancreatic ductal adenocarcinoma (PDAC) patients with PD-L1-expressing macrophages. UALCAN (https://ualcan.path.uab.edu/) was used for the TCGA analysis. miRNA target prediction analysis was done using miRTargetLink 2.0 (https://ccb-compute.cs.uni-saarland.de/mirtargetlink2), TargetScan (https://www.targetscan.org/vert_80/) and RNA22 (https://cm.jefferson.edu/rna22/) was used for the target site predictions in TLR4.

## Supplementary information

Supplementary File

Supplementary Table S1

Supplementary Table S2

Supplementary Table S3

Supplementary Table S4

No EVs control for EVs Internalization

hTERT-HPNE EVs internalization by BMDMs

PANC-1 EVs internalization by BMDMs

PPCL-68 EVs internalization by BMDMs

Original and uncropped western blots

## Data Availability

Data generated in this study are available upon request. The raw metabolomics data files were uploaded on Dryad database (10.5061/dryad.hdr7sqvqr) and the small RNA and the mRNA sequencing data are available in GEO database (GSE285193). The cryoTEM images were uploaded on EMPIAR database^109^ (https://www.ebi.ac.uk/empiar/) with accession number (EMPIAR-13172).
