# Supplementary material for: Extracellular vesicle-derived miRNA-182-5p educates macrophages towards an immunosuppressive phenotype in pancreatic cancer
Source: Signal Transduct Target Ther. 2026 Jan 16;11:31. doi: 10.1038/s41392-025-02559-3 (PMC12811262; doi:10.1038/s41392-025-02559-3)
Supplement: Supplementary file 1 — Supplementary File [file 41392_2025_2559_MOESM1_ESM.docx]

**Supplementary Materials for**

**Extracellular vesicle-derived miRNA-182-5p educates macrophages towards an immunosuppressive phenotype in pancreatic cancer**

Baldev Singh^1^, Pankaj Gaur^1,2^, Pritha Bose^1^, Yanjun Zhang^3^, Yaoxiang Li^1^, Zihao Zhang^1^, Jeyalakshmi Kandhavelu^1^, William Klotzbier^1^, Meth Jayatilake^1^, Shivani Bansal^1^, Mohd Farhan^1^, Sunain Deol^1^, Partha P Banerjee^3^, Keith Unger^4^, Seema Gupta^1,2^, Vivek Verma^5,6^ and Amrita K Cheema^1,3^ **^*^**

Correspondence to: **Amrita K Cheema**, Email: [akc27@georgetown.edu](mailto:akc27@georgetown.edu)

**This file includes:**

Materials and Methods for metabolomics study

Supplementary Figures. S1 to S8

Captions for videos S1 to S4

**Other Supplementary Materials for this manuscript include the following:**

Videos S1 to S4

Table S1. Significantly altered metabolites in PaCa-treated macrophages (intracellular)

Table S2. Significantly altered metabolites in PaCa-treated macrophages (cellular supernatant)

Table S3. Differentially expressed microRNAs in PANC-1 and PPCL-68 EVs as compared to the hTERT-HPNE EVs

Table S4. Details of the reagents and resources used in the study

Graphical Abstract

Original and uncropped images of the western blots

**Materials and Methods for metabolomics study**

**Targeted metabolomic profiling** **mass spectrometry data acquisition**

The targeted MRM-MS based metabolomics method, developed in-house, was used to quantitate >450 metabolites (and two internal standards) with 263 transitions in the positive ion mode and 224 transitions in the negative ion mode using QTRAP® 7500 LC-MS/MS System (Sciex, MA, USA). Five microliters of the prepared sample were injected onto a Kinetex F5, 2.6 μm 100 Å 150 × 2.1 mm (Phenomenex, CA, USA) using a SIL-30 AC auto sampler (Shimadzu) connected to a high flow LC-30AD solvent delivery unit (Shimadzu) and Exion 30AD communication bus module (Shimadzu) online with QTRAP 7500 (Sciex, MA, USA) operating in MRM mode with positive and negative polarity switching method. The details of mobile phase compositions, LC conditions, and column and sampling parameters used are provided in Table 1. Also, the details of MS source and acquisition parameters used for LC-MRM-MS based targeted metabolomics method are provided in Table 2.

The quality and reproducibility of LC-MS data was ensured using several measures. The column was conditioned using the pooled QC samples initially and were also injected periodically to monitor shifts in signal intensities and retention time as measures of reproducibility and data quality of the LC-MS data. We also ran NIST plasma periodically (after every 10 samples) prepared in the same manner to check the instrument variance. Solvent blanks were also run between sets of samples to check for carry-over effects.

**Table 1. Details of LC parameters for LC-MRM-MS based targeted metabolomics method.**

|  | | | | | |
| --- | --- | --- | --- | --- | --- |
| Mobile Phase Compositions | Solvent Flow and Gradient | | | LC Column and Sampling Parameters | |
| A: Water + 0.1% formic acid | Total Flow | 0.200 mL/min | | Type | Kinetex F5 |
|  | Time (mins) | % A | % B | Length | 150 mm |
|  | 0 | 100 | 0.0 | Internal Diameter | 2.1 mm |
| B: Acetonitrile + 0.1% formic acid | 2.1 | 100 | 0.0 | Particle Size | 2.6 µm |
|  | 3.1 | 100 | 0.0 | Temperature | 30 °C |
|  | 14.0 | 5 | 95.0 | Autosampler Temp. | 15 °C |
| Rinse Solvent: 1:1:1:1 water/IPA/MeOH/ACN | 16.0 | 5 | 95.0 | Injection Volume | 2 µL |
|  | 16.1 | 100 | 0 | Needle Wash Time | 2 s |
|  | 20.0 | 100 | 0 | Pressure Limit (Max) | 15,000 psi |
|  | Stop Time | 20.0 mins | |  |  |

**Table 2. Details of MS source and acquisition parameters for LC-MRM-MS based targeted metabolomics method**

|  | | | | |
| --- | --- | --- | --- | --- |
| Turbo Spray Source Settings | Value | | Turbo Spray Source Settings | Value |
| Heater Gas Temperature (°C) | 500 | | Heater Gas (psi) | 60 |
| Curtain Gas Pressure (psi) | 45 | | Collision Gas | 10 |
| Capillary Voltage (+) | 2000 | | Ion Source Gas 1 | 45 |
| Capillary Voltage (-) | -4500 | | Ion Source Gas 2 | 70 |
| Constant MRM Settings | | | | |
| Parameter | Value | Parameter | | Value |
| MRM Detection Window (s) | 90 | Pause Time (ms) | | 2.00 |
| Q1 Resolution | Unit | Settling Time (ms) | | 18.788 |
| Q3 Resolution | Unit | Target Cycle Time (ms) | | 650 |
| Polarity | Positive and Negative | Minimum Dwell Time (ms) | | 3.0 |
|  |  | Maximum Dwell Time (ms) | | 80.0 |

**Mass spectrometry data processing and analysis**

The LC-MRM-MS data were processed using MultiQuant 3.0.3 (Sciex), peak picking parameters used are detailed in Table 3. The processed data were filtered using a signal/noise ratio >20:1 and retention time (RT) tolerance of 5 s, after manually checking of metabolites peak by experts to find the reliable features. The data were normalized to the intensity of internal standards (debrisoquine was used as positive mode internal standard and 4-nitrobenzoic acid was used as negative mode internal standard). We also use 20% of missing values in each feature as filter out criteria. Missing values were imputed by half of the minimum positive value in the original data. Thereafter, we used 20% of the coefficient of variation (CV) as our filter criteria to remove any possible noises before data normalization. Analytical drifts (if any) were corrected by quality control based robust LOESS signal correction (QC-RLSC).^100^ The high-quality filtered data were then used for the statistical analyses. All the statistical analyses for LC-MRM data were performed using MetaboAnalyst (V5.0) which involved pre-processing techniques like log transformation and Pareto scaling.

**Table 3. Details of peak picking parameters for LC-MRM-MS based targeted metabolomics processing method**

|  | Peak picking for parameters | Details |
| --- | --- | --- |
|  | Integration Algorithm | MQ4 (MultiQuant 3.0.3) |
|  | RT Half Window | 30.0 s |
|  | Gaussian Smooth Width | 0.0 points |
|  | Update Expected RT | No |
|  | Report Largest Peak | Yes |
|  | Min. Peak Width | 3 points |
|  | Min. Peak Height | 0.00 |
| Integration Parameters | Noise Percentage | 40.0 % |
|  | Baseline Sub. Window | 2.00 mins |
|  | Peak Splitting | 3 points |








**Supplementary Fig. 1. Isolation, characterization and internalization of EVs by BMDMs (supporting data for Fig. 1).**

**(a-g)** Characterization of extracellular vesicles (EVs) isolated using size exclusion chromatography (SEC). **(a)** Nanoparticle tracking analysis (NTA) data showing size distribution for EVs preparations; (**b)** EVs concentration data obtained through NTA shows higher EVs yields from PaCa-derived EVs as compared to non-tumorigenic cell line hTERT-HPNE; **(c)** NTA based mean particle size of hTERT-HPNE, PANC-1 and PPCL-68 EVs shows uniform size distribution across cell lines; **(d)** NTA characterization of mT3-2D EVs isolated using SEC. **(e-f)** Representative immunoblots showing expression of EV associated markers. **(g)** Cryogenic Transmission Electron Microscopy (Cryo-TEM) imaging show membrane integrity of EV. **(h-i)** Representative confocal microscopy images of murine BMDMs treated with PKH67-labeled EVs (green), showing accumulation of EVs near endoplasmic reticulum (ER); ER were stained with ER-Tracker™ Red dye (yellow), BMDMs were stained with cell mask deep red (red), and nucleus was stained with Hoechst stain (blue). **(h)** A single stack of the 3-D constructed image; **(i)** Birds-eye and perpendicular view of macrophages**.** All images were captured after 24 h of EVs treatment at 63x magnification with pseudo color selection for each dye channel and PBS without any EVs mixed with PKH67 dye was used as a negative control. All data are presented as Mean ± SE, where minimum n = 3 per treatment group. ns = p > 0.05, * = p ≤ 0.05, ** = p ≤ 0.01, *** = p ≤ 0.001 and **** = p ≤ 0.0001 by unpaired *t* test.








**Supplementary Fig. 2. Internalization of EVs by THP-1 macrophages and BMDMs (supporting data for Fig. 1).**

**(a-b)** Representative confocal microscopy images of THP-1 macrophages (human) treated with PKH67-labeled EVs (green), showing internalization of EVs; THP-1 macrophages were stained with cell mask deep red (red), and nucleus was stained with Hoechst stain (blue); **(a)** A single stack of the 3-D constructed image; **(b)** Birds-eye and 3D horizontal view of a macrophage. All images were captured after 24 h of EVs treatment at 63x magnification with pseudo color selection for each dye channel. PBS without any EVs mixed with PKH67 dye was used as a negative control. **(c)** Representative confocal microscopy images of GFP expressing murine BMDMs treated with PKH26-labeled mT3-2D (murine PDAC cell line) EVs (Red), showing internalization of EVs, BMDMs (green), and nucleus was stained with Hoechst stain (blue). **(d)** Gating strategies used to analyze EV uptake by BMDMs. **(e)** Flow cytometric analysis showing time-dependent uptake (MFI) of PKH67-labeled EVs by BMDMs at different time intervals (3, 6, 12 and 24 h post EV treatment). **(f)** Gating strategies used to analyze EVs uptake by THP-1 macrophages. **(g)** Flow cytometric analysis shows time-dependent uptake (MFI) of PKH67-labeled EVs by THP-1 macrophages (human) at different time intervals (3, 6, 12 and 24 h post EV treatment). All data are presented as Mean ± SE, where minimum n = 3 per treatment group. ns = p > 0.05, * = p ≤ 0.05, ** = p ≤ 0.01, *** = p ≤ 0.001 and **** = p ≤ 0.0001 by unpaired *t* test.








**Supplementary Fig. 3. PaCa-EVs skew macrophages towards M2-like TAMs (supporting data for Fig. 1).**

**(a)** Flow cytometry gating strategies used to analyze peritoneal cavity resident macrophages (PCMs), after EV treatments. **(b)** Flow cytometry gating strategies used to analyze CD86 and CD206 expression in mice BMDMs after EV treatments. **(c)** Representative immunofluorescence images showing higher CD206 expression in PANC-1 and PPCL-68 EV-treated BMDMs as compared to untreated and hTERT-HPNE-treated BMDMs. **(d)** Gene expression analysis of *CD80*, *CD86, and Arginase1* in BMDMs treated with mT3-2D EVs (20 μg/ml) for 48 h. **(e)** Schematic representation of the experiment used to determine the effect of PaCa cell-derived EVs on THP-1 macrophages polarization. **(f)** Representative flow cytometry plots and bar graphs showing CD206 expression in THP-1 macrophages after 72 h of EVs (10 μg/ml) treatment. **(g)** Flow cytometry gating strategies used to analyze CD206 expression in THP-1 macrophages, 72 h after EV treatments. **(h)** The concentration of anti-inflammatory (IL-6, IL-10, CCL17 (TARC), and IL-1RA) and pro-inflammatory cytokines (TNF-α and IL-1β) in the cellular supernatant of EV-treated THP-1 macrophages. **(i)** PCA analysis of secretory metabolomic profiles of EV-treated BMDMs. **(j and k)** Volcano plots showing robust alterations in metabolite abundance in cellular supernatant from PANC-1 and PPCL-68 EV-treated BMDMs as compared to hTERT-HPNE EV-treated BMDMs (q ≤ 0.05). **(l)** Bar graphs showing differential abundance of some key secretory metabolites in BMDMs after 72 h of EV treatment. All data are presented as Mean ± SE, where n ≥ 3 per condition. ns = p > 0.05, * = p ≤ 0.05, ** = p ≤ 0.01, *** = p ≤ 0.001 and **** = p ≤ 0.0001 by one-way ANOVA and unpaired *t* test (selected metabolites).








**Supplementary Fig. 4. Gating strategies used to analyze T cell proliferation (supporting data for Fig. 2).**

**(a)** Flow cytometry gating strategy used to analyze CD3^+^ T cell proliferation for evaluating CD4^+^ and CD8^+^ T cell immune suppression by mT3-2D EVs (murine PDAC cell line EVs)-treated macrophages under *in vitro* conditions. **(b)** IL-2 and activation controls for contact dependent *in vitro* immune suppression (shown in Fig. 2d) by hTERT-HPNE, PANC-1 and PPCL-68 EV-treated macrophages. **(c-d)** Effect of mT3-2D EVs on CD4^+^ and CD8^+^ T cell proliferation respectively. **(e)** IL-2 and activation controls for the contact independent *in vitro* immune suppression (given in Fig. 2f-h) by hTERT-HPNE, PANC-1 and PPCL-68 EV-treated macrophages. **(f)** Flow cytometry gating strategies used to analyze blood and spleen samples for evaluating CD8^+^ T cell proliferation and activation in adoptive cell transfer experiment. All data are presented as Mean ± SE, where n ≥ 3 per condition. ns = p > 0.05, * = p ≤ 0.05, ** = p ≤ 0.01, *** = p ≤ 0.001 and **** = p ≤ 0.0001 by one-way ANOVA and unpaired *t* test (selected metabolites).











**Supplementary Fig. 5. Immunosuppressive function of PaCa-EVs educated macrophages depends on PD-L1 (supporting data for Fig. 3).**

**(a)** Flow cytometry data showing rescue of T cell (CD8^+^) suppression mediated by PANC-1 and PPCL-68 EV (PaCa-EV) educated macrophages in the presence of anti-PD-1 and anti-PD-L1 mAb. **(b)** Respective IL-2 (non-proliferation) and activation (proliferation) controls for the above experiment. **(c)** Flow cytometry data shows rescue of PaCa-EV educated macrophages-mediated T cell suppression in the presence of anti-IL-10 and anti-TGF-β mAb. **(d-e)** Characterization of PPCL-46, Capan-1, MIA PaCa-2 and SW-1990 cell line-derived EVs; **(d)** Nanoparticle tracking analysis (NTA) data showing uniform size distribution across all EV preparations; **(e)** Immunoblot analysis showing EV-associated marker expression across different cell lines. **(f**) Flow cytometry data showing the M1 and M2 markers expression on BMDMs, after different PaCa cell lines (PANC-1, PPCL-68, PPCL-46, CAPAN-1, MIA PaCa-2, and SW-1990) derived EV treatment as compared to hTERT-HPNE EVs treatment. **(g**) PCA of mRNA expression profiling of EV-treated BMDMs. **(h)** Venn-diagram showing the number of differentially expressed genes in PANC-1 and PPCL-68 EV-treated macrophages (BMDMs) as compared to hTERT-HPNE EVs (q ≤ 0.05) (n = 3). **(i)** Heatmap of significantly up and down-regulated pro- and anti-inflammatory pathway related genes in macrophages (n = 3). (**j)** Pathways analysis of differentially expressed genes. **(k)** Inhibition of JAK/STAT3 signaling in BMDMs using ruxolitinib (RUXO) treatment, shows reduced phospho-STAT3 expression. **(l)** Inhibition of JAK/STAT3 signaling in BMDMs using ruxolitinib (RUXO) prior to PaCa-EV treatment shows reduced pJAK2, pSTAT3, ARG1 and PD-L1 expression upon western blot analysis. **(m)** Inhibition of JAK/STAT3 signaling prior to PaCa-(PPCL-68) EV treatment rescues T cell suppression in BMDMs (n = 5)**.** Data are presented as Mean ± SE, where n = 4 per condition, unless specified. ns = p > 0.05, * = p ≤ 0.05, ** = p ≤ 0.01, *** = p ≤ 0.001 and **** = p ≤ 0.0001 by one-way ANOVA and unpaired *t* test.








**Supplementary Fig. 6. PaCa-EVs enriched miR-182-5p polarize macrophages towards M2-like phenotype by down-regulating TLR4 (supporting data for Fig. 4).**

**(a)** Flow cytometry analysis based bar graph shows changes in CD206 and PD-L1 expression following miR-182-5p mimic and NC mimic transfection in BMDMs. **(b)** Flow cytometry analysis based bar graph showing alterations in CD206 and PD-L1 following miR-182-5p inhibitor and NC inhibitor transfection in BMDMs, **(c)** Western blot analysis showing changes in TLR4, and ARG1 expression in miR-182-5p inhibitor transfected BMDMs (24 h), followed by 48 h of IL-4 + IL-13 treatments (20 ng/mL each). **(d)** Representative IVIS (i, ii), ultrasound (iii, iv) and isolated pancreas (after euthanasia) (v, vi) images for the Pan02 tumor bearing and healthy mice. **(e)** Flow cytometry analysis of pancreatic tissue digest showing higher CD206^+^ and PD-L1^+^ macrophages in Pan02 tumor bearing mice as compared to the non-tumor controls (n = 3), whereas CD86^+^ cells were reduced. **(f)** Gating strategies used to analyze macrophage population in pancreatic tumor digests of mice. **(g)** qRT-PCR data shows higher miR182-5p expression in pancreas from tumor bearing mice as compared to the normal pancreas. **(h)** Nanoparticle tracking analysis (NTA) data of plasma-derived EV preparations from healthy donors and PDAC patients. **(i)** Immunoblot analysis shows EV-associated marker expression in healthy donors’ and PDAC patients’ plasma-derived EV preparations. **(j)** Characteristics of the patient cohort used in the study. Data are presented as Mean ± SE, where minimum n = 3 per condition, unless specified. ns = p > 0.05, * = p ≤ 0.05, ** = p ≤ 0.01, *** = p ≤ 0.001 and **** = p ≤ 0.0001 by unpaired *t* test.








**Supplementary Fig. 7. Inhibition of miR182-5p in PaCa cells impairs the ability to polarize macrophages to M2-like TAM phenotype (supporting data for Fig. 5).**

**(a)** Nanoparticle tracking analysis (NTA) data showing uniform size distribution among PPCL-68 EVs isolated from different treatment groups (vehicle control, NC inhibitor, and miR-182-5p inhibitor). **(b-d)** Immunoblot analysis shows EV-associated marker expression across PPCL-68 EVs isolated from different treatment groups; **(b)** vehicle control, **(b)** NC inhibitor, and **(d)** miR-182-5p inhibitor. **(e)** CyQuant proliferation assay of THP-1 macrophages treated with PPCL-68 EVs isolated from different treatment groups (vehicle control, NC inhibitor, and miR-182-5p inhibitor) shows no cytotoxic effect at different time intervals (24, 48 and 72 h after EV treatment). **(f-h)** CyQuant, PrestoBlue, and XTT assays analysis of THP-1 macrophages treated with PPCL-68 EVs isolated from different treatment groups (vehicle control, NC inhibitor, and miR-182-5p inhibitor), showing no cytotoxic effect (48 h after EV treatment). **(i-k)** Flow cytometry analysis of THP-1 macrophages (24 h after treatment) showing no EV uptake differences among PKH67-labeled PPCL-68 EVs isolated from different treatment groups (vehicle control, NC inhibitor, and miR-182-5p inhibitor). **(l)** Gating strategies showing the flow cytometry analysis of Pan02 tumor digest for macrophages and CD8^+^ T cells. **(m)** Bar graph shows increase in the number of CD8^+^ T cells in antagomiR-182-5p treatment group as compared to the vehicle control and NC inhibitor treatment groups, normalized to tumor burden. Data are presented as Mean ± SE, where n = 4 per condition, unless specified. ns = p > 0.05, * = p ≤ 0.05, ** = p ≤ 0.01, *** = p ≤ 0.001 and **** = p ≤ 0.0001 by one-way ANOVA.





**Supplementary Fig. 8. Evaluating the combinational effect of miR-182-5p inhibitor and gemcitabine treatments on mT3-2D tumor growth in immunocompetent mice (supporting data for Fig. 6).**

**(a)** Gating strategies showing the flow cytometry analysis of mT3-2D tumors digest for macrophages and **(b)** CD8^+^ T cells.

**Videos**

**S1.** No EVs control for EVs Internalization

**S2.** hTERT-HPNE EVs internalization by BMDMs

**S3.** PANC-1 EVs internalization by BMDMs

**S4.** PPCL-68 EVs internalization by BMDMs
