# Supplementary figures and images for: Extracellular vesicle-derived miRNA-182-5p educates macrophages towards an immunosuppressive phenotype in pancreatic cancer
Source: Signal Transduct Target Ther. 2026 Jan 16;11:31. doi: 10.1038/s41392-025-02559-3 (PMC12811262; doi:10.1038/s41392-025-02559-3)

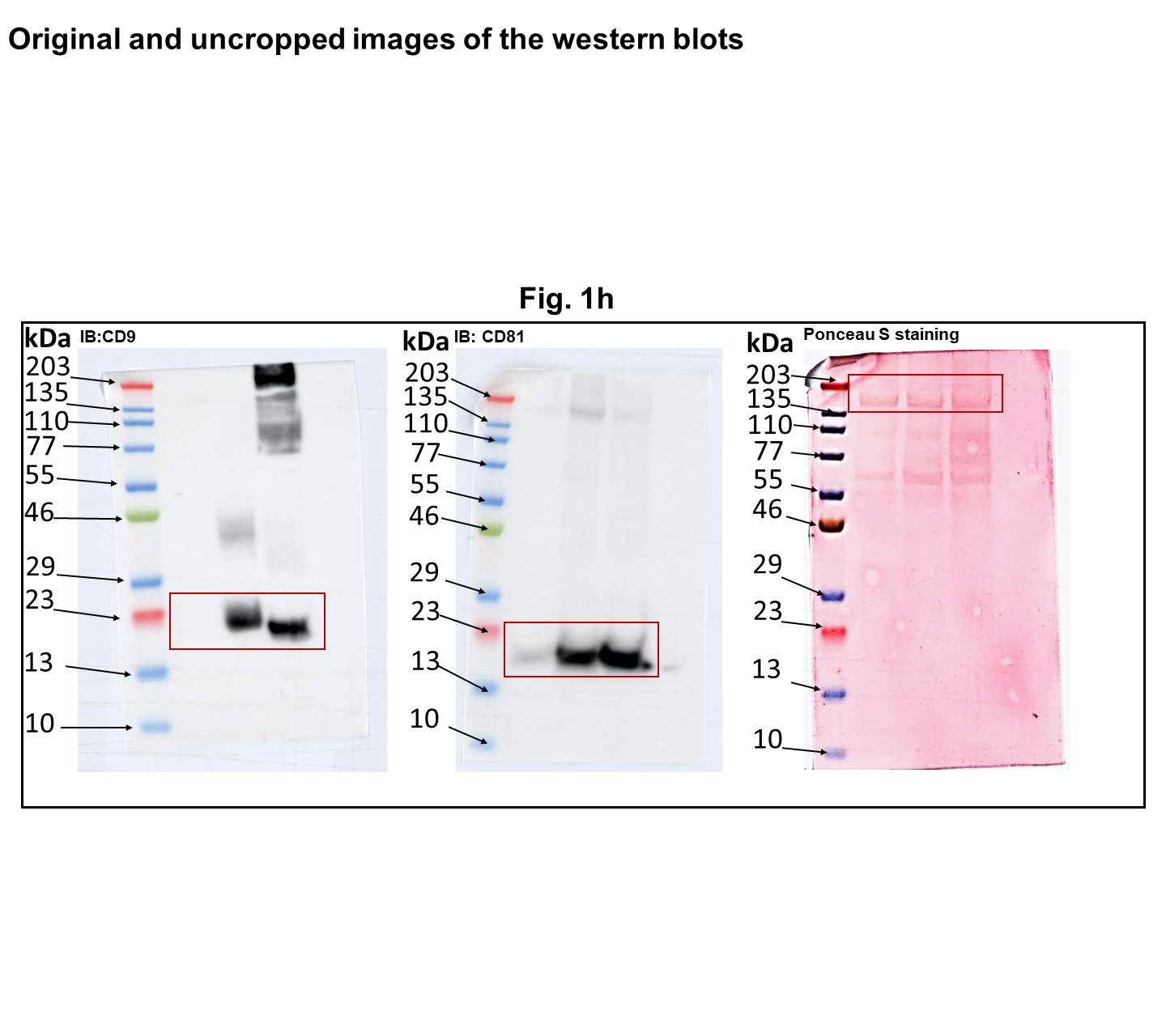

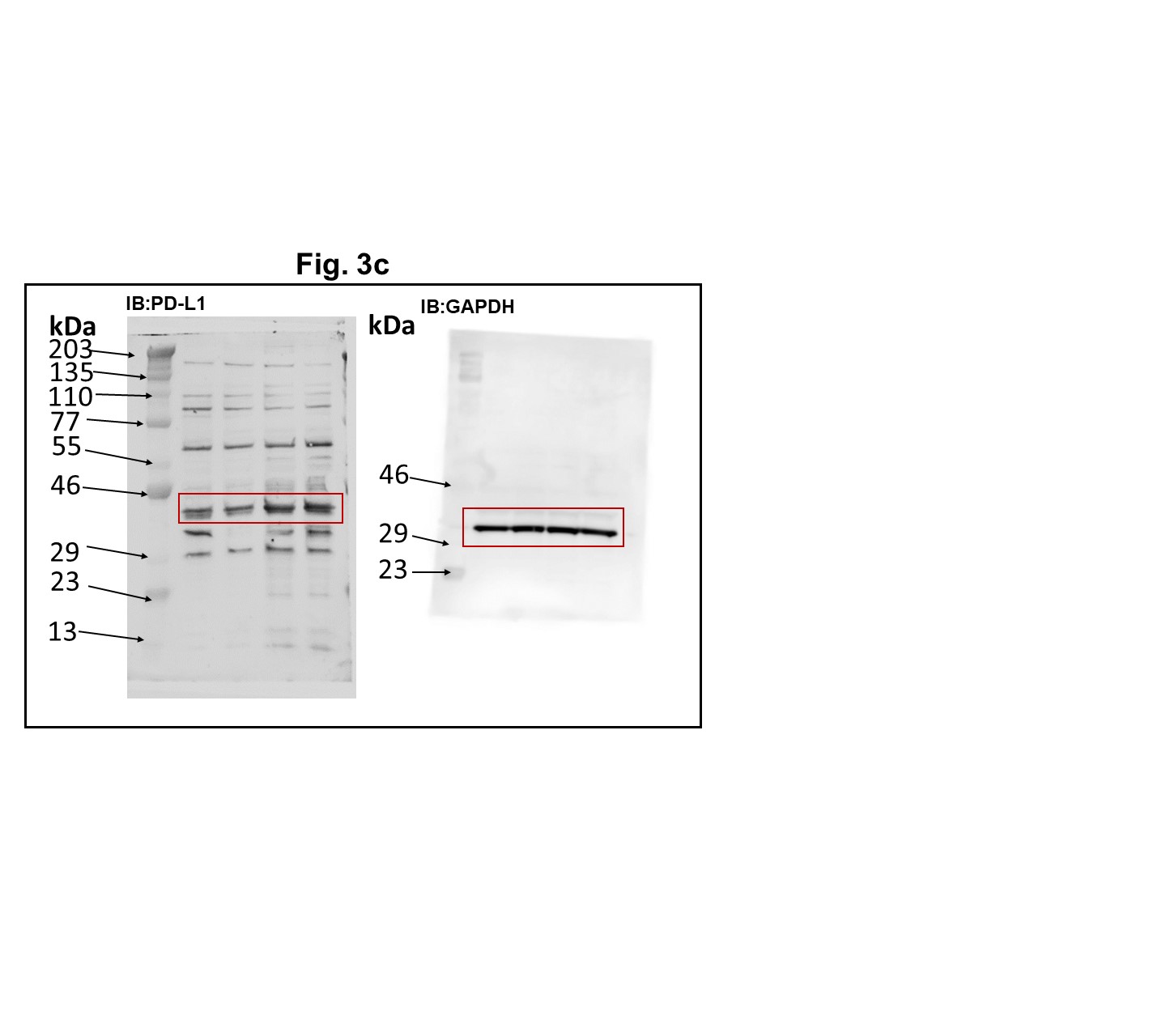

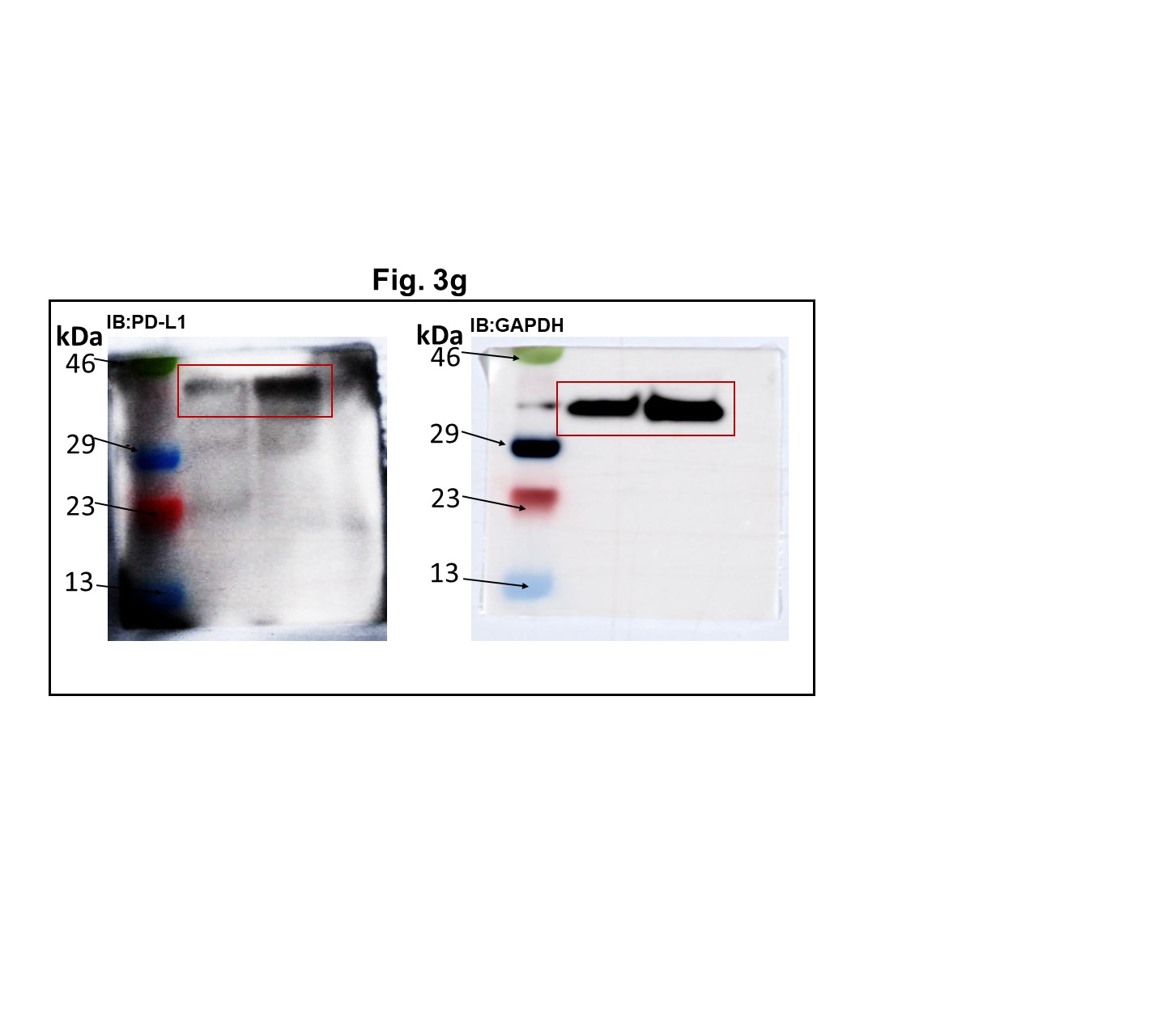

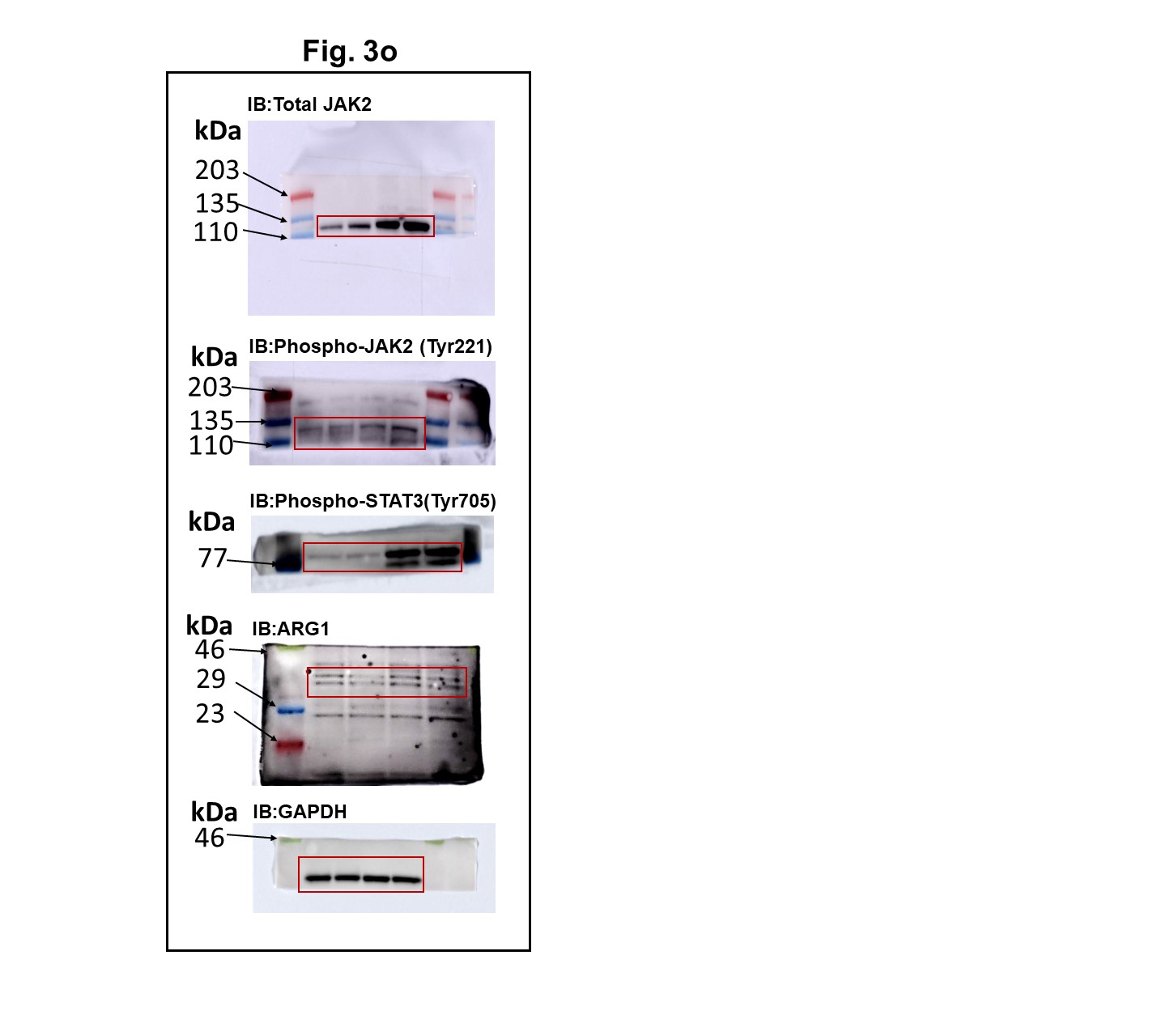

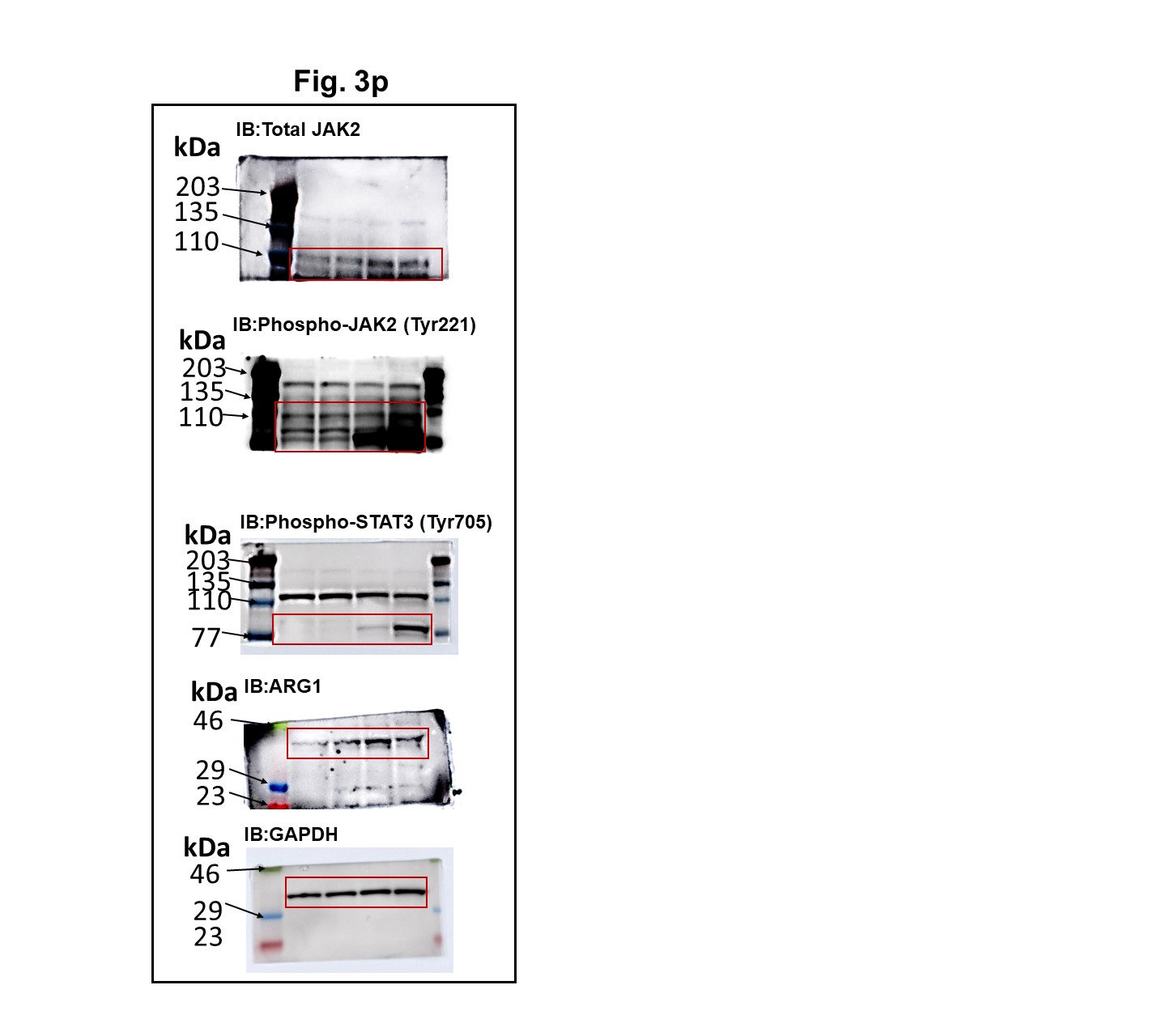

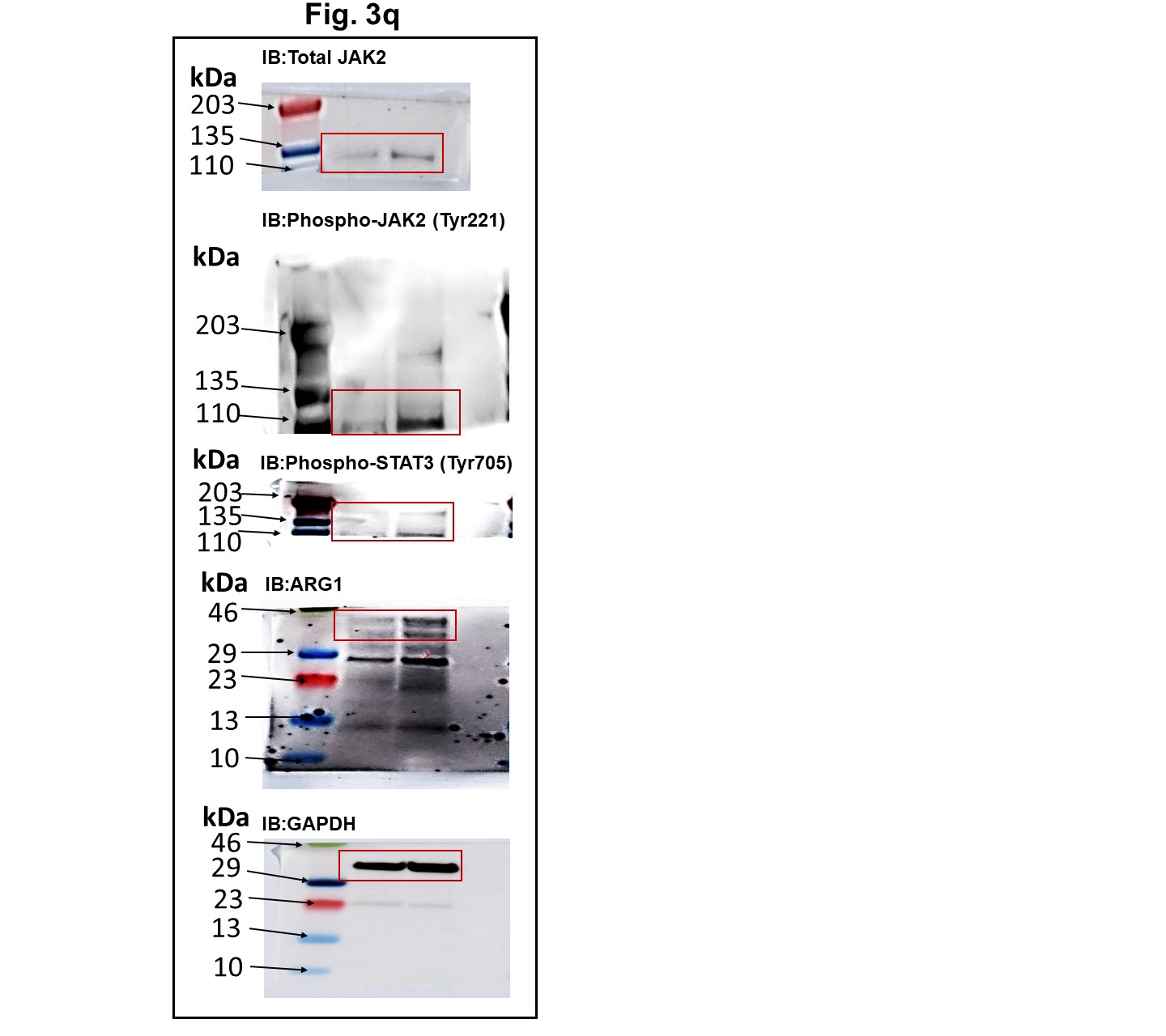


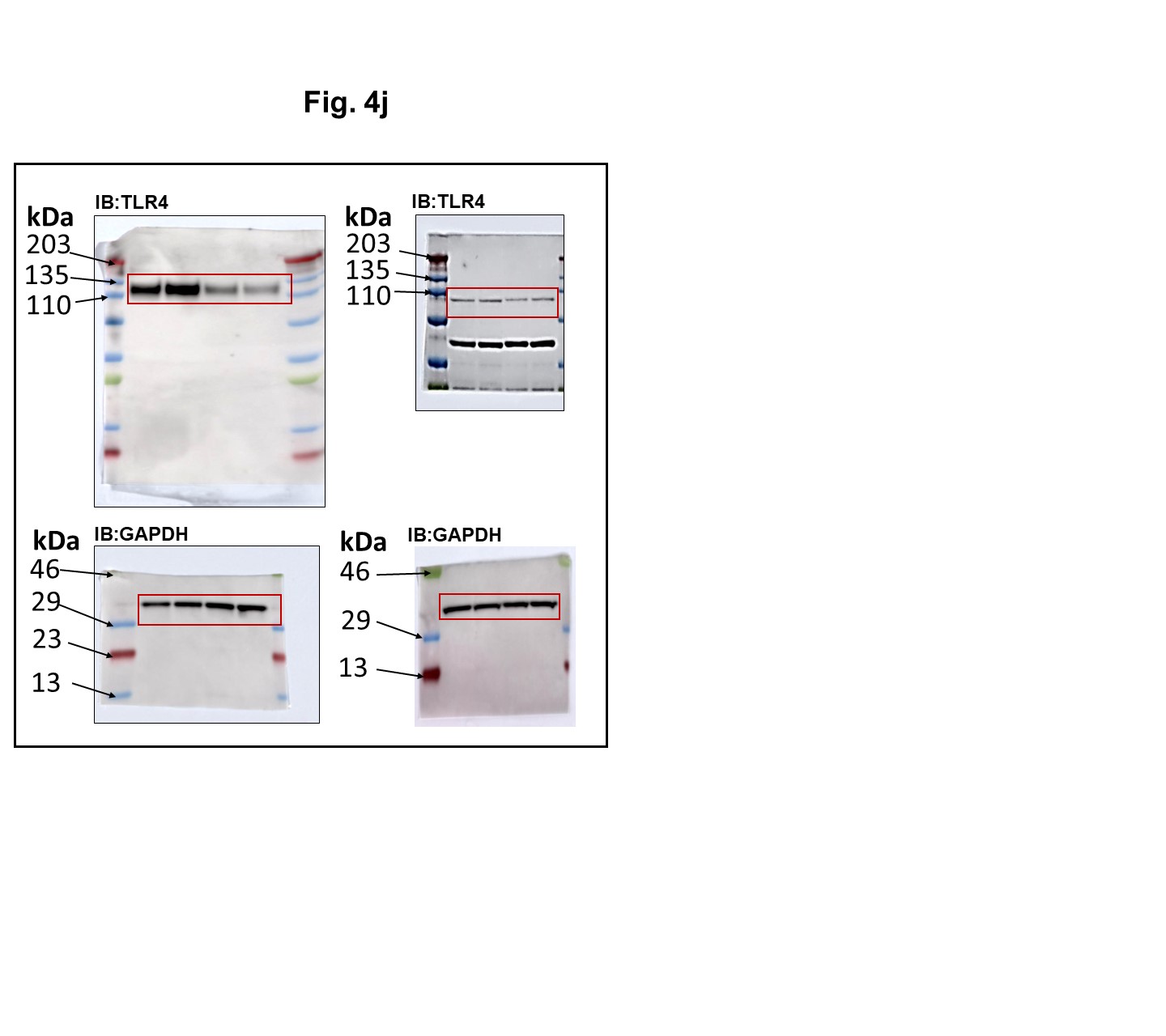

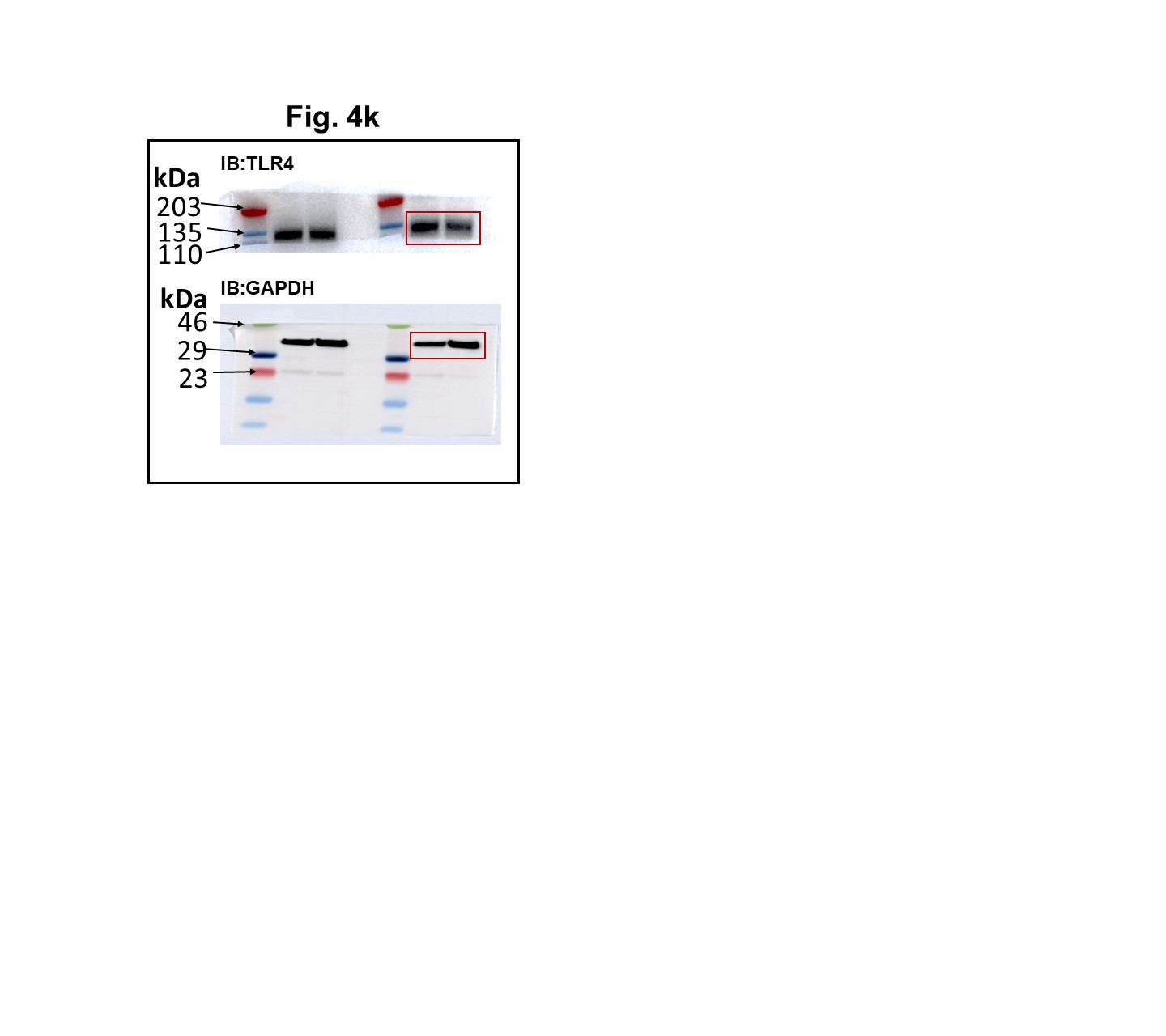

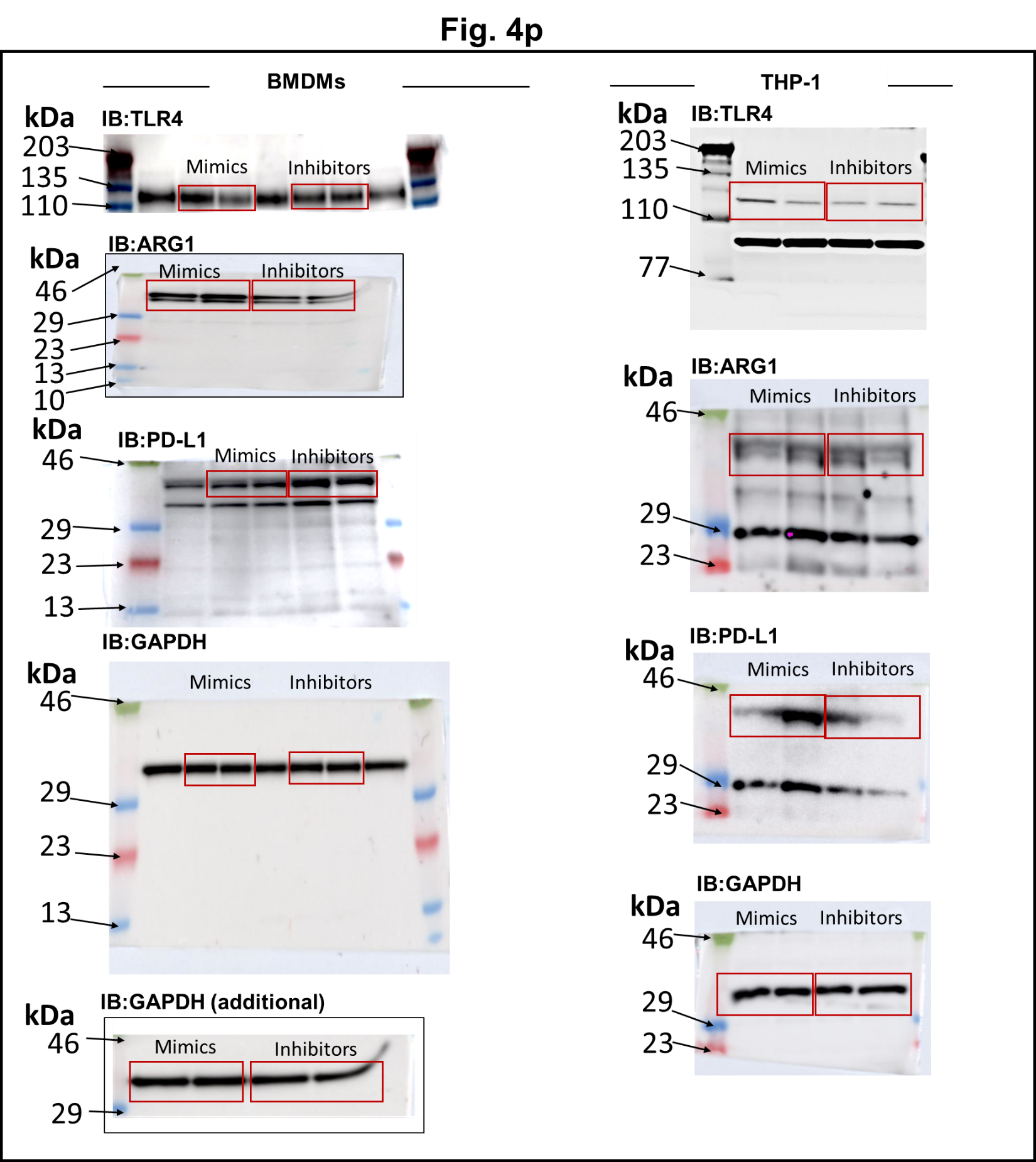


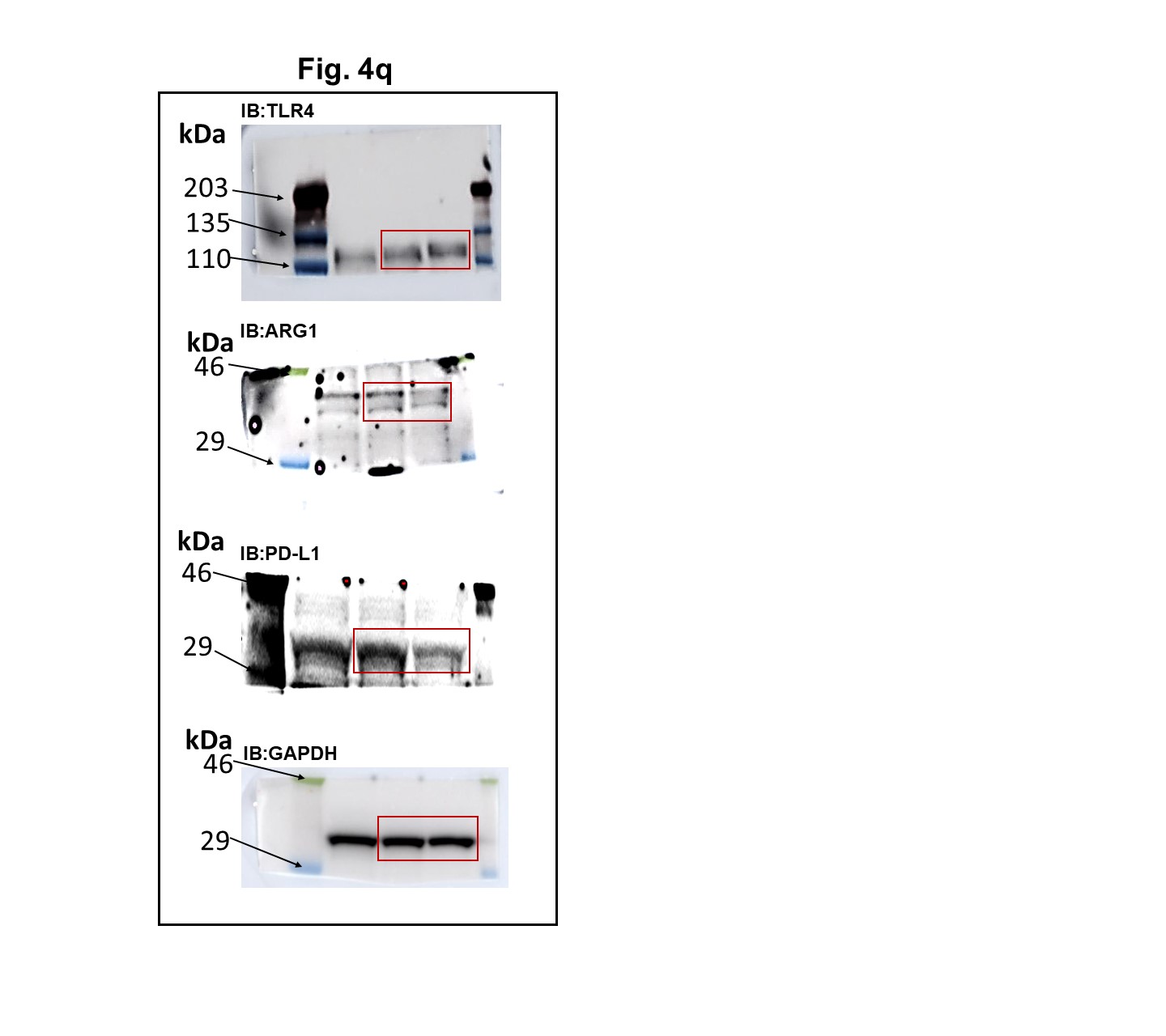

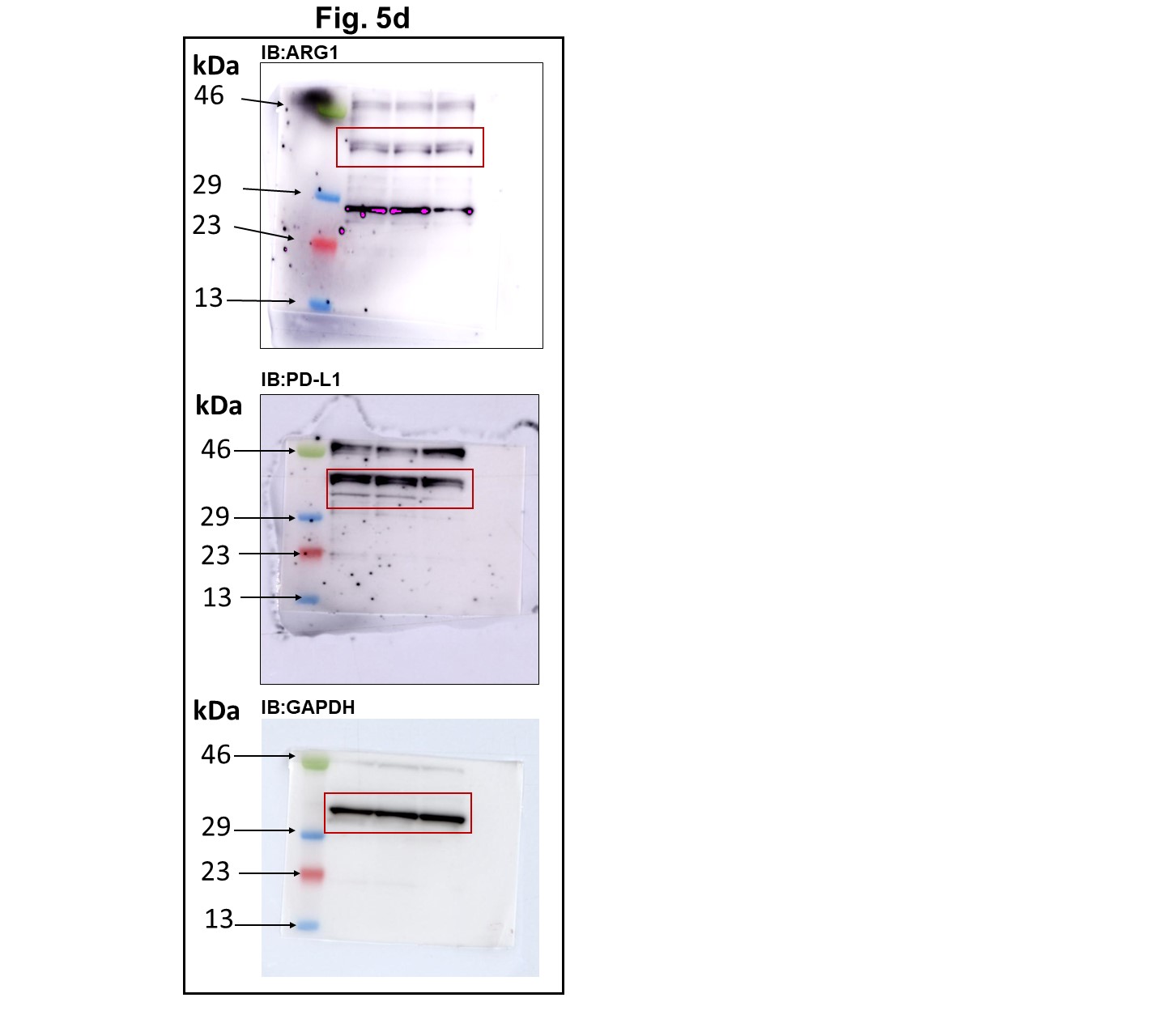


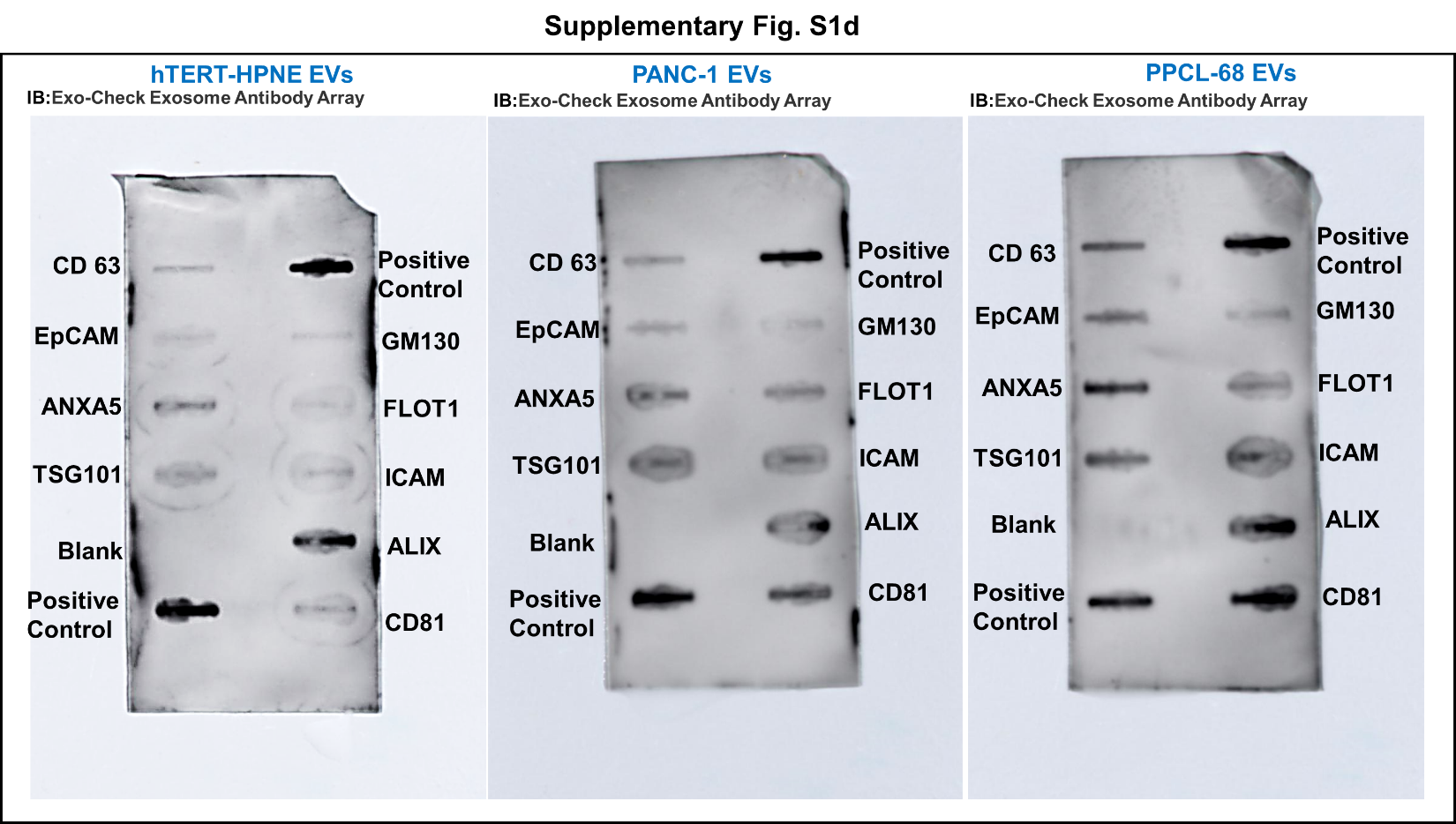

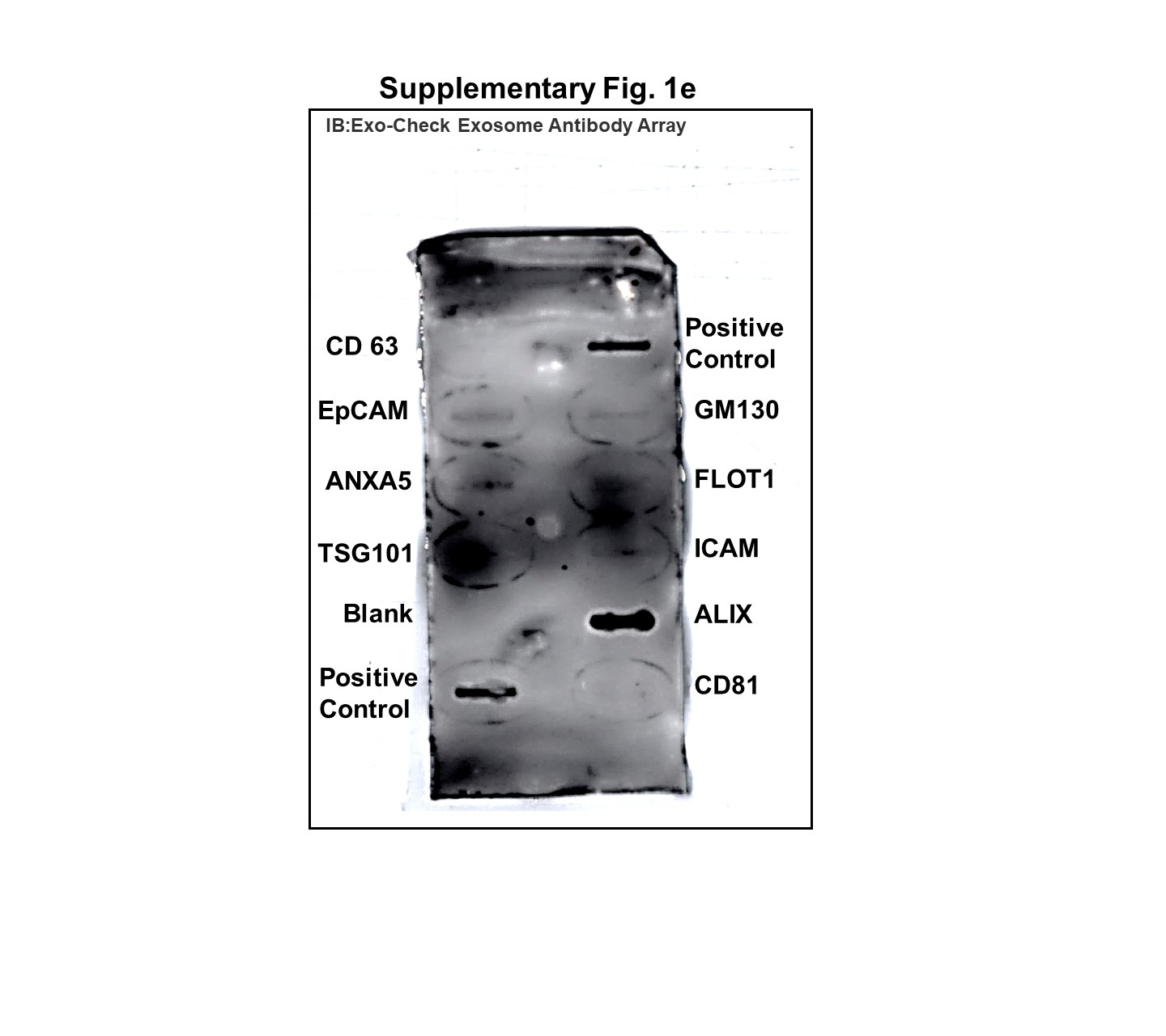

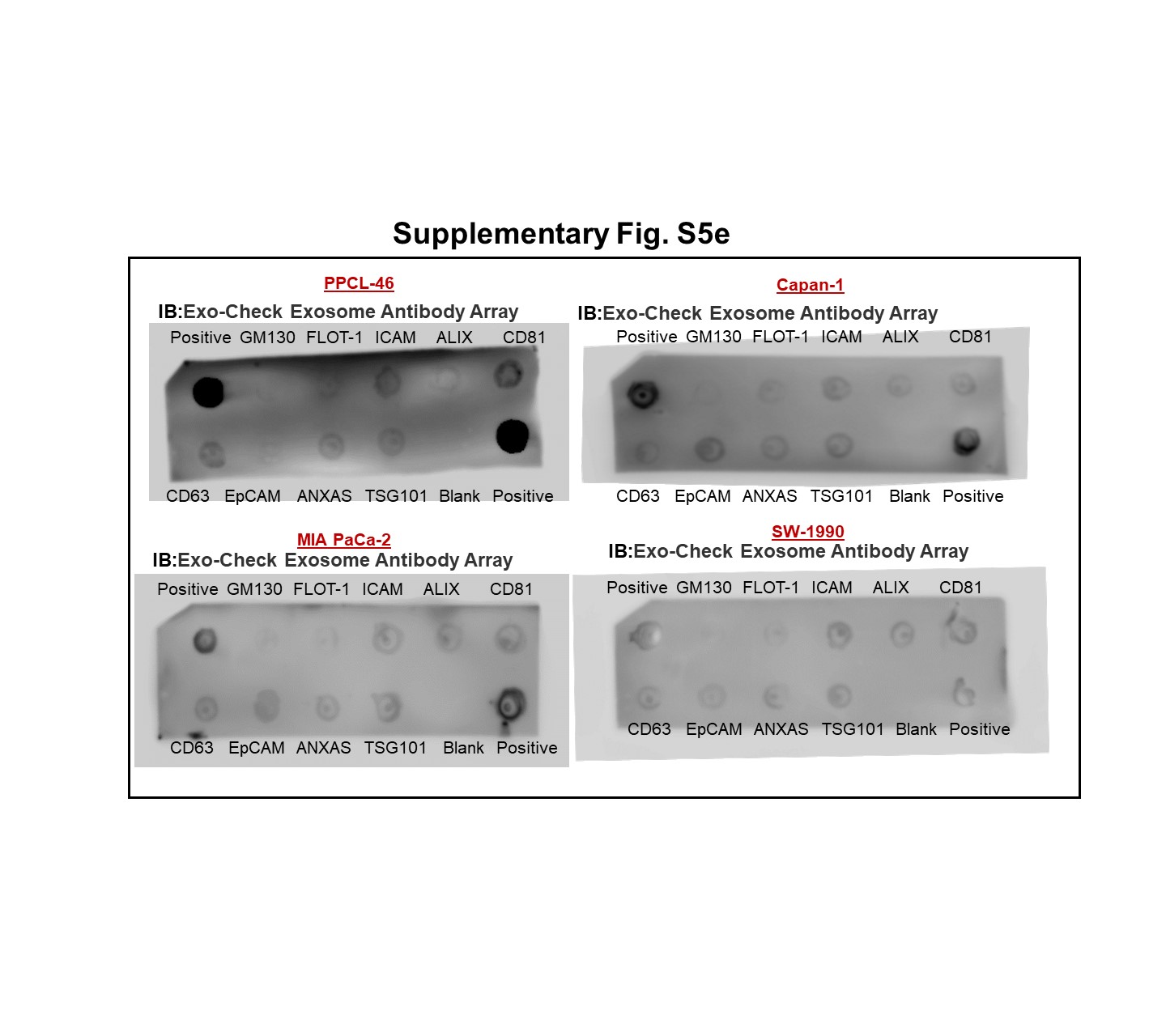

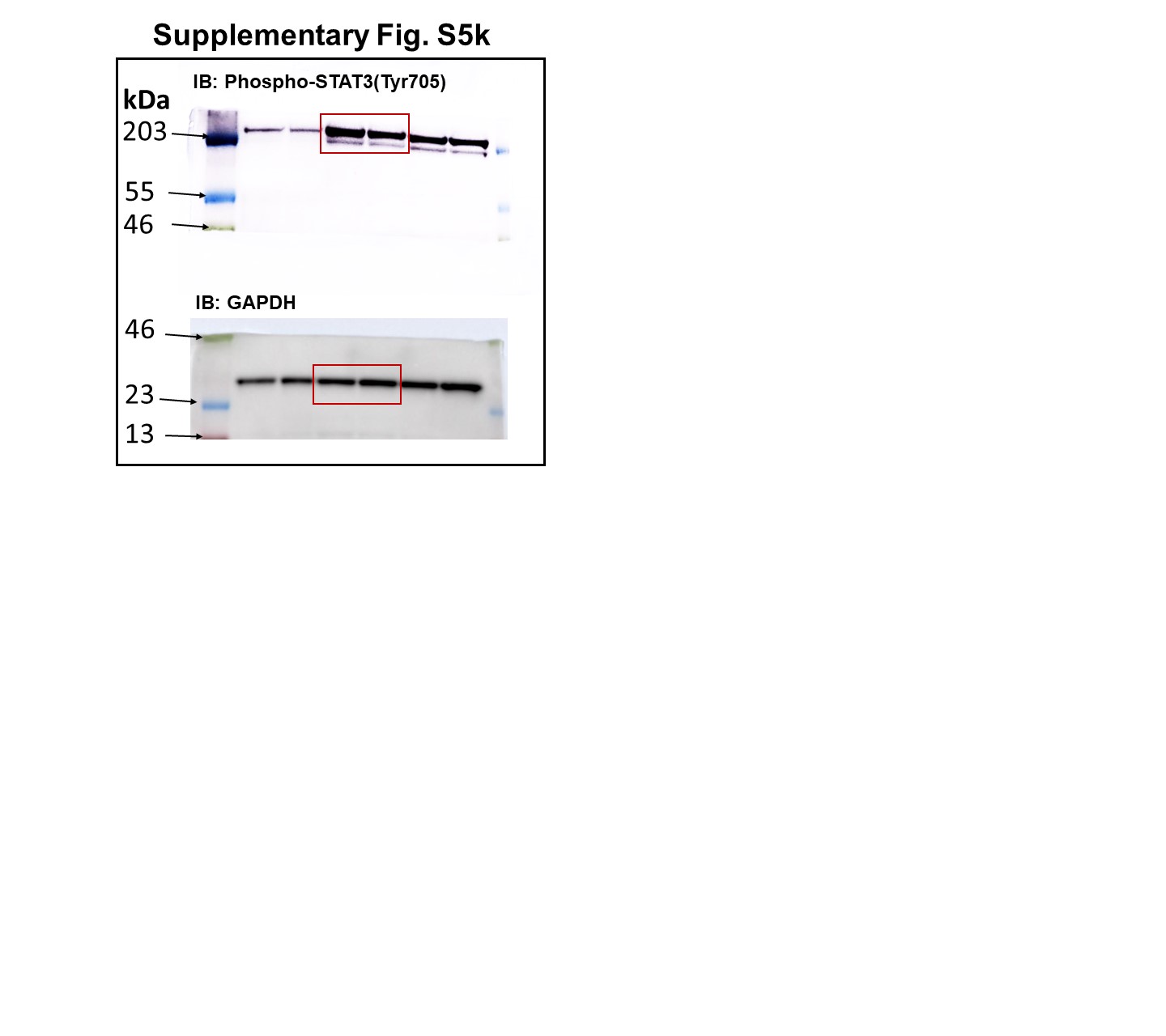

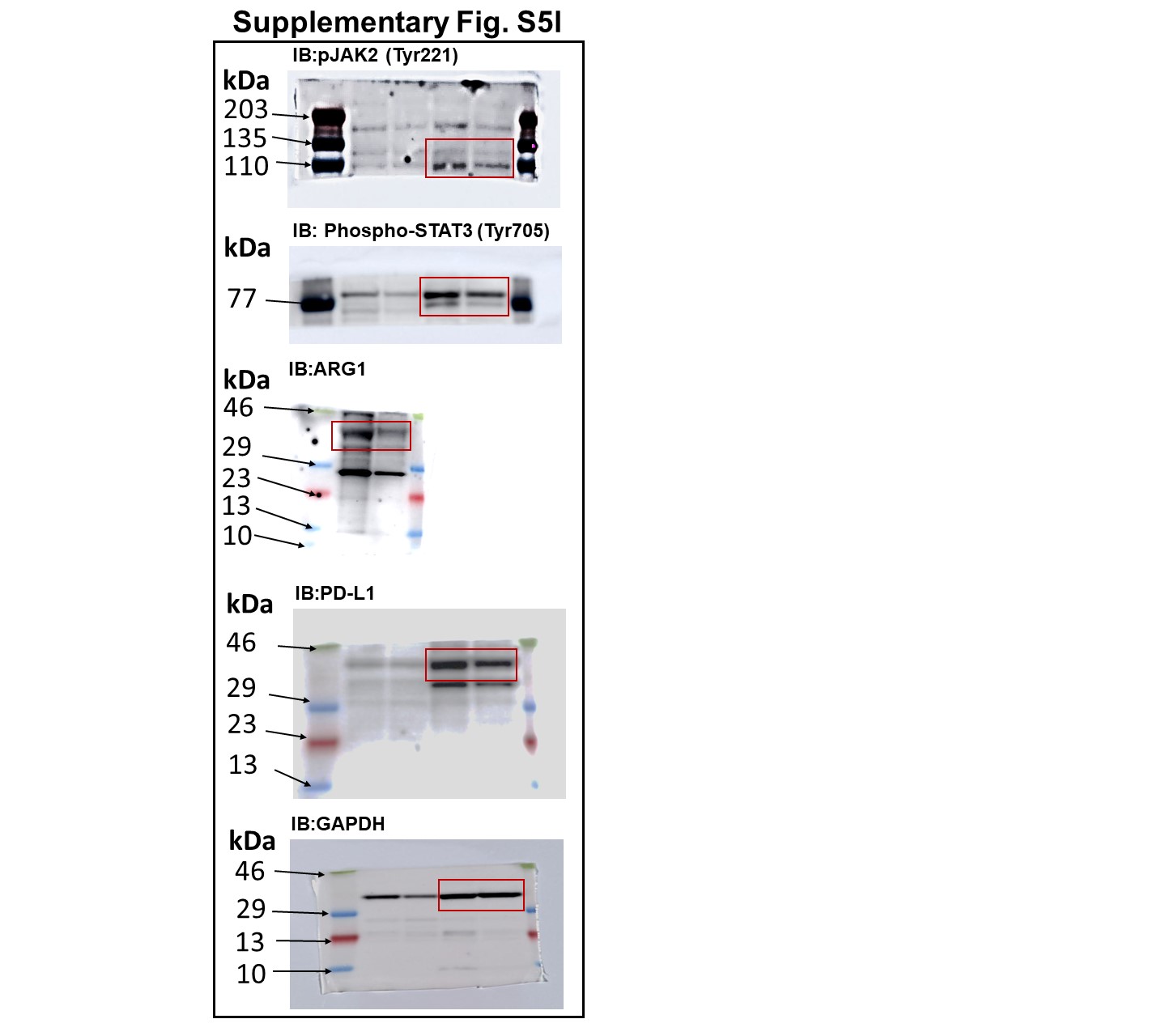

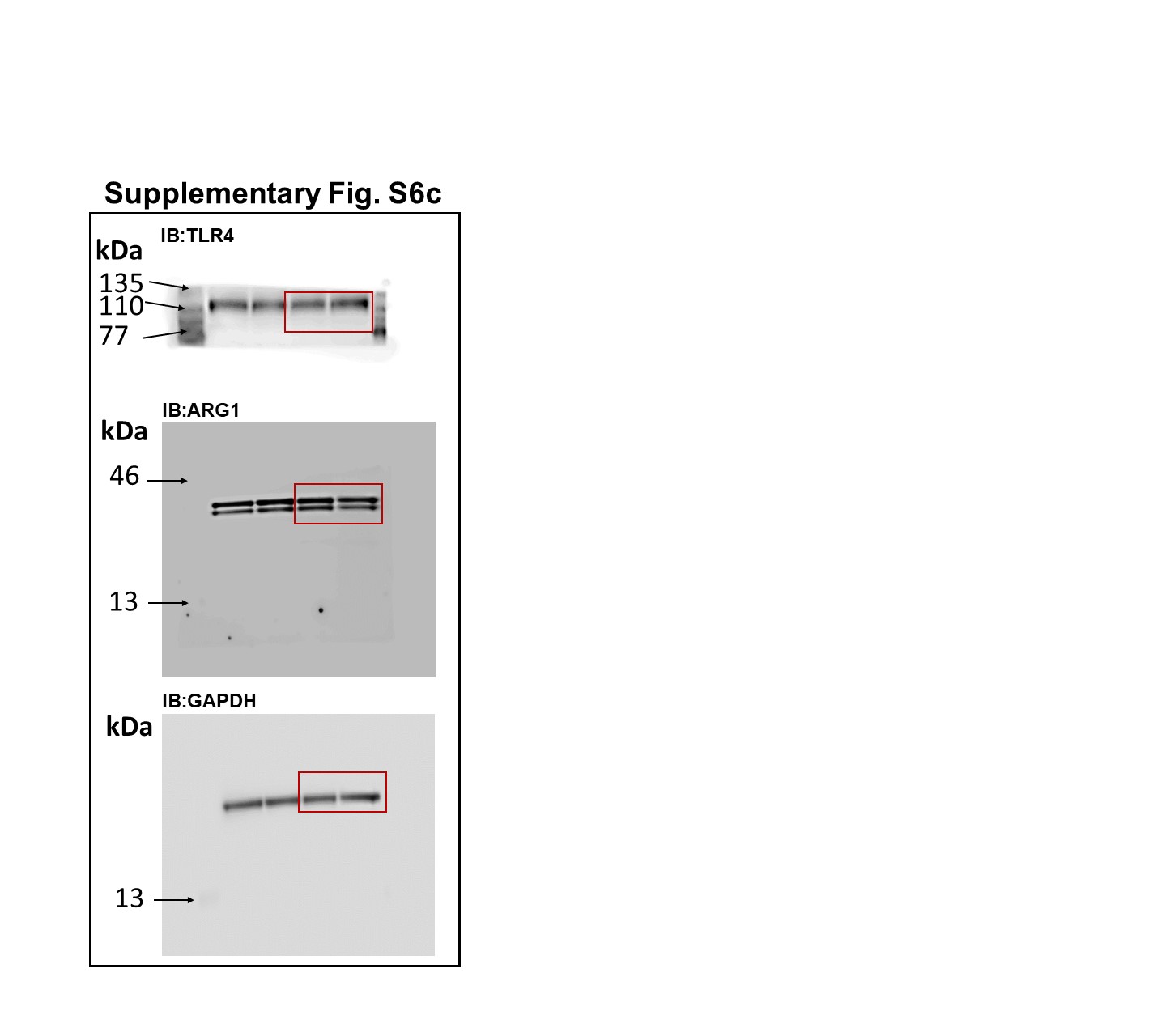

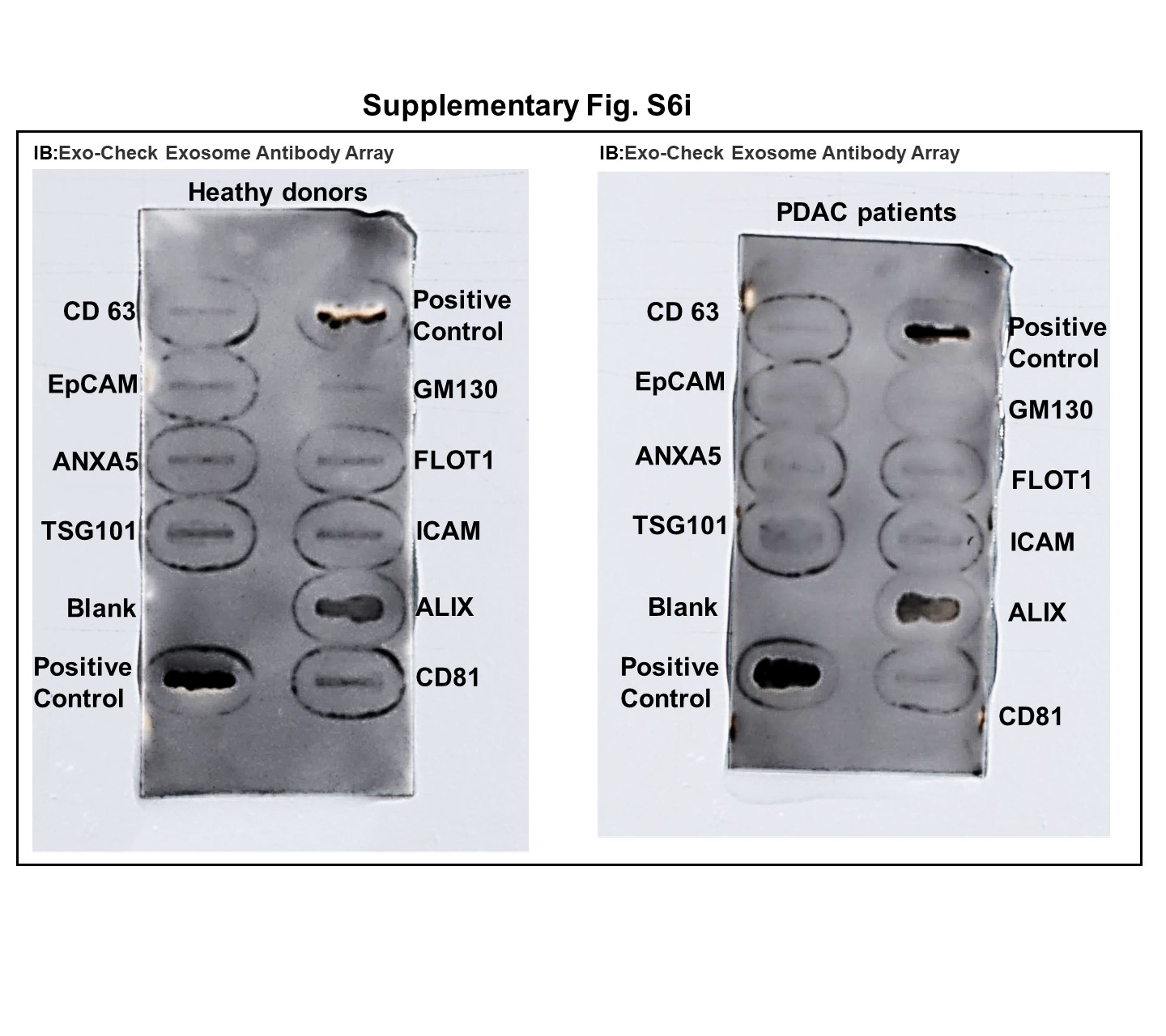

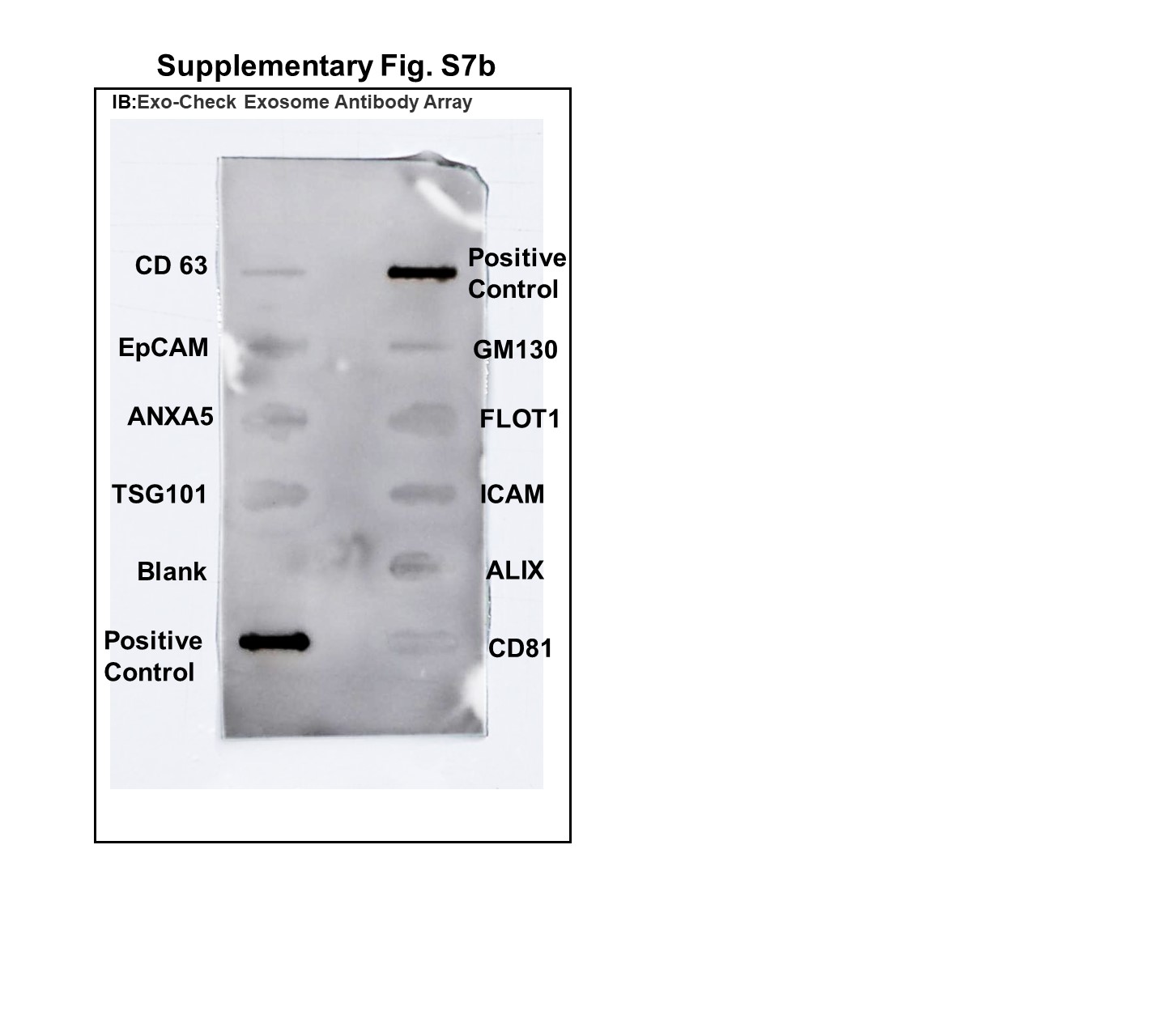

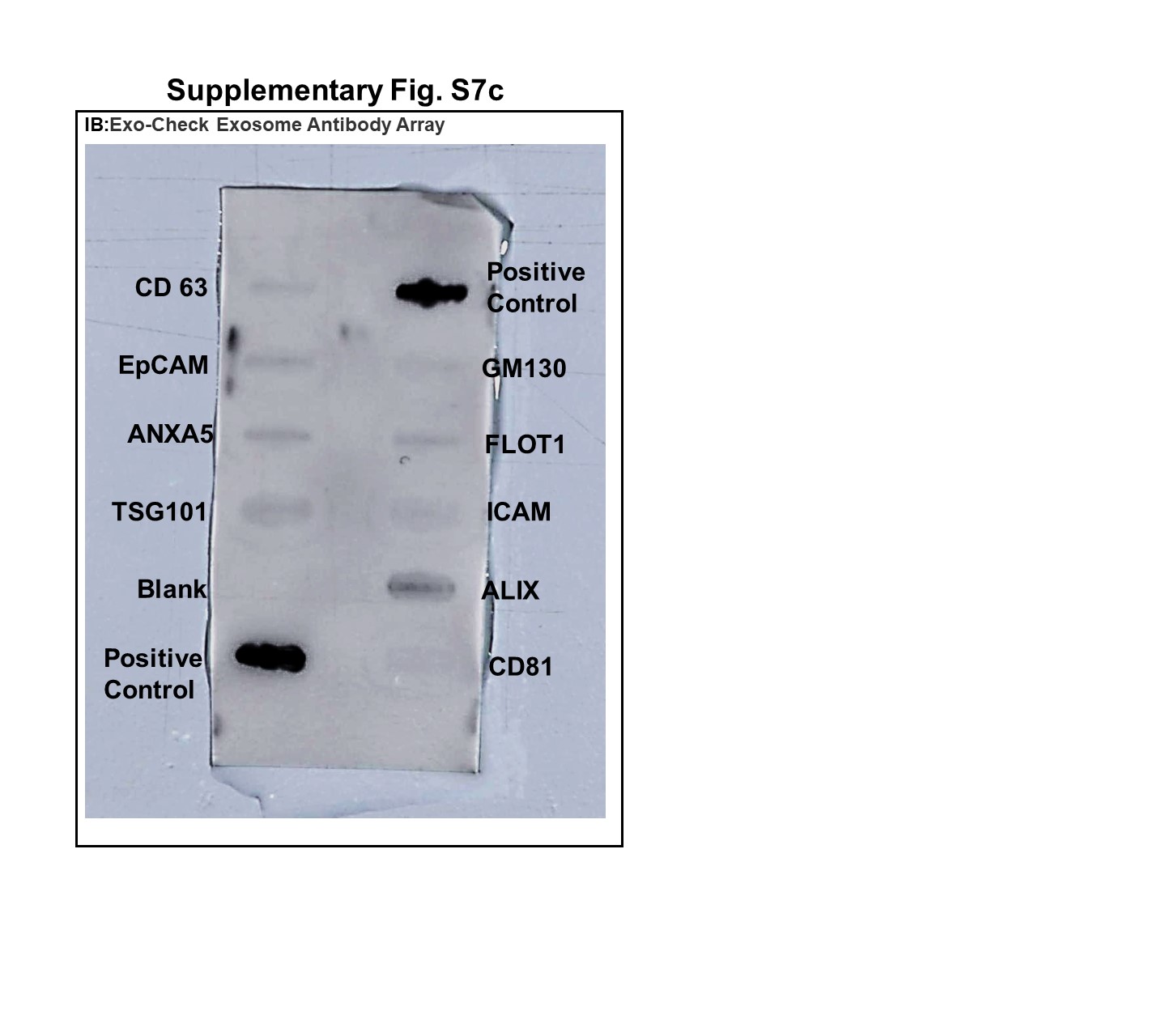

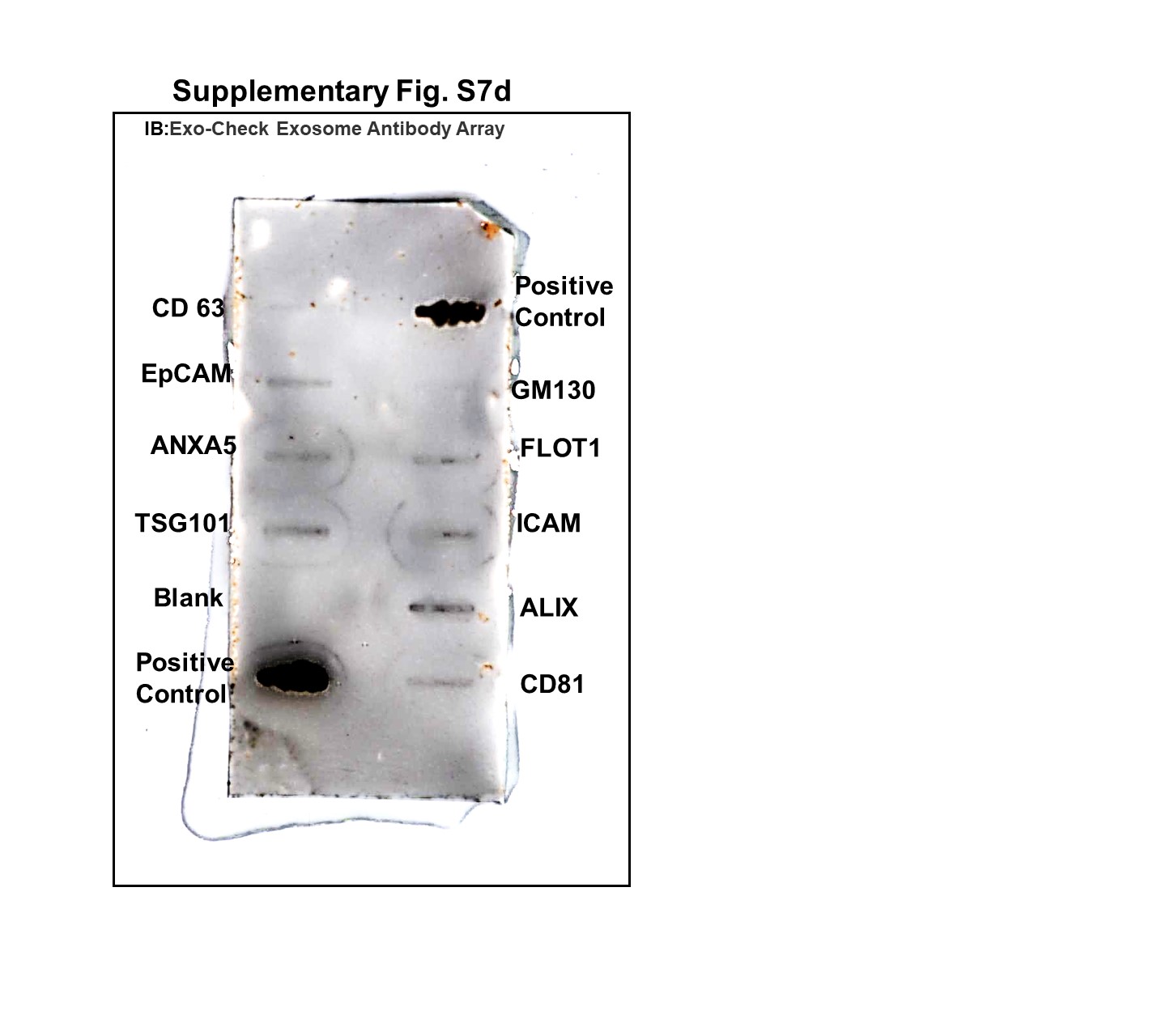

Supplement: Supplementary file 10 — Original and uncropped western blots [file 41392_2025_2559_MOESM10_ESM.docx]
